# Aging-Related Impairments to M Cells in Peyer’s Patches Coincide With Disturbances to Paneth Cells

**DOI:** 10.3389/fimmu.2021.761949

**Published:** 2021-12-06

**Authors:** David S. Donaldson, Barbara B. Shih, Neil A. Mabbott

**Affiliations:** The Roslin Institute & Royal (Dick) School of Veterinary Studies, University of Edinburgh, Easter Bush, United Kingdom

**Keywords:** aging, M cells, follicle-associated epithelium, Paneth cells, ACE2, transcriptomics

## Abstract

The decline in mucosal immunity during aging increases susceptibility, morbidity and mortality to infections acquired *via* the gastrointestinal and respiratory tracts in the elderly. We previously showed that this immunosenescence includes a reduction in the functional maturation of M cells in the follicle-associated epithelia (FAE) covering the Peyer’s patches, diminishing the ability to sample of antigens and pathogens from the gut lumen. Here, co-expression analysis of mRNA-seq data sets revealed a general down-regulation of most FAE- and M cell-related genes in Peyer’s patches from aged mice, including key transcription factors known to be essential for M cell differentiation. Conversely, expression of ACE2, the cellular receptor for SARS-Cov-2 virus, was increased in the aged FAE. This raises the possibility that the susceptibility of aged Peyer’s patches to infection with the SARS-Cov-2 virus is increased. Expression of key Paneth cell-related genes was also reduced in the ileum of aged mice, consistent with the adverse effects of aging on their function. However, the increased expression of these genes in the villous epithelium of aged mice suggested a disturbed distribution of Paneth cells in the aged intestine. Aging effects on Paneth cells negatively impact on the regenerative ability of the gut epithelium and could indirectly impede M cell differentiation. Thus, restoring Paneth cell function may represent a novel means to improve M cell differentiation in the aging intestine and increase mucosal vaccination efficacy in the elderly.

## Introduction

The epithelial cell lining in the intestine provides a tightly-sealed protective barrier against the commensal and pathogenic microorganisms in the lumenal contents. However, a specialized subset of phagocytic epithelial cells known as M cells is present in the follicle-associated epithelia (FAE) that cover the gut-associated lymphoid tissues (GALT) such as the Peyer’s patches in the small intestine. These unique epithelial cells actively sample particulate antigens present in the gut lumenal contents and transcytose them into the GALT (1) where they are subsequently sampled by mononuclear phagocytes and B cells present in the M cell basolateral pocket (2, 3). This M cell-mediated immunosurveillance of the intestinal contents is essential for maintaining germinal centre responses in Peyer’s patches (3), the induction of antigen-specific mucosal immune responses against certain pathogenic bacteria (4–6), as well as the effective development of IgA antibody responses against commensal bacteria (7).

The mucosal immune system is adversely affected by aging and this functional decline is known as immunosenescence (8). As a consequence, infections acquired *via* the gastrointestinal and respiratory tracts are more common in the elderly and are associated with increased morbidity and mortality. Using aged mice (≥18 months old) we have shown that this immunosenescence in the mucosal immune system impairs the functional maturation of M cells (9, 10). The density of M cells in the FAE of aged mice is much lower than that observed in young mice and negatively affects the ability to sample pathogenic bacteria such as *Salmonella enterica* subsp. Typhimurium in the gut lumen (9, 10). Interestingly, *Citrobacter rodentium* infection causes a more severe pathology in the intestines of M cell-deficient mice, mirroring the increased severity of intestinal infections observed in elderly humans (11). The reduced ability of M cells in aged mice to sample the intestinal contents also impedes the induction of IgA responses against orally applied antigens (10).

Little is known of the factors responsible for this aging-associated decline in M cell functional maturation. However, we showed that this deficit is reversible as exposure of aged mice to microbial stimuli such as flagellin or a commensal microbiota from young mice can restore M cell maturation and function in their Peyer’s patches (10). Gut epithelial cells, including M cells, derive from Lgr5+ intestinal stem cells (ISC) situated in the base of intestinal crypts (12, 13). The intestinal crypts are similarly affected by aging (10, 14–16). Since the microbial stimulation of aged mice was accompanied by increased intestinal crypt function (10), it is plausible that the impaired functional maturation of M cells was a consequence of an aging-related decline in intestinal crypt function. A greater understanding of how aging negatively impacts on M cell differentiation and function will help identify novel methods to reverse this aging-related decline and increase the efficacy of mucosal vaccines against gastrointestinal and respiratory pathogens. Therefore in the current study we used a graph based co-expression analysis approach to investigate the effects of aging on the transcriptional profile in the FAE, the conventional villous epithelium (VE) and ileum.

## Materials and Methods

### Mice

C57BL/6J mice were purchased from Charles River (Margate, UK). RANK^ΔIEC^ and RANK^F/F^ mice (7), also on a C57BL/6J background, were bred and maintained at the University of Edinburgh, UK. Mice were maintained in-house under specific pathogen-free conditions to the ages required. Young mice were used at 6-16 weeks old, aged mice were used at 24-28 months old. All the experiments described in this study were first approved by The Roslin Institute’s Ethical Review Committee, and were conducted under the authority of a UK Home Office project license in full compliance with the Animals (Scientific Procedures) Act 1986.

### FAE Sheet and VE Preparation

Isolated FAE sheets were prepared as described previously (17–19). Briefly, portions of intestine containing individual Peyer’s patches were removed and immersed in Hank’s balanced salt solution (HBSS) containing 30 mM EDTA at 4°C for 15 min. Peyer’s patches domes were micro-dissected and the FAE layer then carefully teased away from the underlying tissue using fine needles under stereomicroscopy and trimmed of villi. Sheets of conventional VE were prepared in the same manner from 5 mm portions of ileum (free of Peyer’s patches) adjacent to each dissected Peyer’s patch. Isolated FAE and VE sheets as well as 5 mm sections of whole ileum from each mouse were stored at -80°C until RNA extraction.

### mRNA-Seq Analysis

Total RNA was isolated from FAE, VE sheets and ileum samples using the RNeasy Plus Kit (Qiagen, Manchester, UK) with a gDNA Eliminator column. mRNA-seq libraries were then prepared using the TruSeq stranded total RNA-seq library preparation kit (Illumina, San Diego, USA) with one round of RiboZero Gold treatment. After quality control for RNA integrity and concentration, 24 individual libraries were retained for RNA-seq: 3 x young FAE; 3 x aged FAE; 4 x young VE; 6 x aged VE; 4 x young ileum; 4 x aged ileum. The libraries were then pooled and sequenced with 150 base paired end reads at a depth of 290M paired end reads/sample across four lanes of an Illumina HiSeq 4000 sequencing platform (Illumina). Reads were trimmed using Cutadapt (version cutadapt-1.9.dev2) (20). Reads were trimmed for quality at the 3’ end using a quality threshold of 30 and for adapter sequences of the TruSeq stranded mRNA kit (AGATCGGAAGAGC). After trimming, reads were required to have a minimum length of 50. Reads were aligned to the *Mus musculus* (GRCh38) genome in Ensembl using STAR (version 2.5.2b) (21) specifying paired-ends and option –outSAMtype BAM Unsorted. All other parameters were set at default. Reads were assigned to features of type ‘exon’ in the input annotation grouped by gene_id in the reference genome using featureCounts (version 1.5.1) (22). Strandness was set to ‘reverse’ and a minimum alignment quality of 10 was specified. A matrix of fragments per kilobase of transcript per million mapped reads (FPKM) values was generated, using the rpkm() function of edgeR (version 3.16.5) (23) and normalized effective library sizes. Gene lengths for the FPKM calculation were the number of bases in the exons of each gene (only counting bases once where they occur in multiple exon annotations). The mRNA-seq data sets are available *via* the following accession code in the Gene Expression Omnibus data base (GEO; www.ncbi.nlm.nih.gov/geo): GSE182252.

### Co-Expression Analysis

First, an expression matrix containing all 24 annotated, non-log transformed, mRNA-seq FPKM data sets was created. This was then imported into the bioinformatics tool Biolayout Express3D (www.biolayout.org) (24–26) and a sample-to-sample Pearson correlation matrix calculated containing an all vs. all comparison of the expression profile of each gene represented. A graph was then plotted using all sample-to-sample relationships ≥ 0.86 with the nodes representing each data set and the edges (connections between them) representing Pearson correlation coefficients of *r* ≥ 0.86.

Then, a pairwise gene-to-gene Pearson correlation matrix was calculated that compared the expression profile of each gene across all 24 data sets. This was used to create a network graph with a Pearson correlation coefficient cut-off threshold of *r* ≥ 0.93. Here the nodes represent individual genes with the edges between them representing Pearson correlation coefficients *r* ≥ 0.93. The Markov clustering algorithm with an inflation value set to 2.2 (which controls the granularity of clustering) was then used to cluster the graph into groups of genes that shared similar profiles across the 24 data sets. Details of the genes within each cluster are provided in **Table S1** and the mean expression profiles of the top 50 largest clusters are provided in **Figure S1**.

Genes in clusters of interest were then assessed for cellular functions and activities using a combination of literature review and bioinformatics. Gene enrichment analyses (significantly over-represented gene ontologies (GO) and significantly enriched transcription factor binding site motifs) in clusters of interest were determined using g:Profiler [https://biit.cs.ut.ee/gprofiler/gost; (27)].

### Immunostaining

To detect M cells by whole-mount immunostaining Peyer’s patches were first fixed using BD Cytofix/Cytoperm (BD Biosciences), and then immunostained with rat anti-mouse GP2 monoclonal antibody (mAb) (MBL International, Woburn, MA). Peyer’s patches were then stained with Alexa Fluor 488-conjugated anti-rat IgG antibodies (Ab) and Alexa Fluor 647-conjugated phalloidin to detect F-actin (both Thermo Fisher Scientific).

For analysis of sections, Peyer’s patches and small intestines were also snap-frozen at the temperature of liquid nitrogen, and 6 μm serial frozen sections cut using a cryostat. To detect B cells the acetone-fixed frozen sections were stained with Alexa-Fluor 488 (green)-conjugated rat anti-mouse CD45R (B220) mAb (clone RA3-6B2; Thermo Fisher Scientific, Loughborough, UK). To detect MPTX1/2, acetone-fixed frozen sections were stained with rabbit anti-MPTX+MPTX2 mAb (clone EPR20920-19; Abcam, Cambridge, UK). SOX8, ACE2 and lysozyme were detected in paraformaldehyde-fixed frozen sections using polyclonal guinea pig anti-SOX8 (19), polyclonal rabbit anti-ACE2 (ab15348; Abcam) and polyclonal rabbit anti-lysozyme (A0099; Agilent, Stockport, UK). To detect Spi-B, paraformaldehyde-fixed frozen sections were treated with citrate buffer before immunostaining with sheep anti-mouse Spi-B polyclonal Ab (R&D Systems, Abingdon, UK).

Where relevant, the sections were co-immunostained with species-specific secondary Ab coupled to Alexa Fluor 488, 594 or 574 dyes (Thermo Fisher Scientific). Cell nuclei were detected using DAPI. Sections were mounted in fluorescent mounting medium (Agilent) prior to imaging on a Zeiss LSM710 confocal microscope (Zeiss, Cambourne, UK).

### Image Analysis

For GP2 analysis, the GP2+ cells were counted in Z-stacks of 4 FAE/mouse (from 2 Peyer’s patches) and the mean GP2+ cell density/mouse determined (n=3-4 mice). For Spi-B and SOX8 analysis, positively immunostained nuclei in the FAE were counted in 4-11 images of FAE from 2-3 Peyer’s patches/mouse (3 mice per group, n=20-29).

Morphometric analysis of microscopy images of MPTX+MPTX2, lysozyme and ACE2 immunostaining was performed using ImageJ software as described previously (28, 29). Background intensity thresholds were first applied using an ImageJ macro which measures pixel intensity across all immunostained and non-stained areas of the images. The obtained pixel intensity threshold value was then applied in all subsequent analyses. Next, the number of pixels in each channel were automatically counted and presented as a proportion of the total number of pixels in each area under analysis. The mean area of MPTX+MPTX2-positive or lysozyme-positive immunostaining was analyzed in three separate images (containing between 4-6 crypts) per mouse (3-4 mice/group, n=9-12/group). For analysis of ACE2-positive immunostaining, between 1-14 images of FAE were analyzed/mouse (3-4 mice/group, n=21-30/group). To compare the height of lysozyme-positive immunostaining in the crypts, the distance from the nuclei at the base of the crypt to the highest observable lysozyme staining was measured in 10-16 crypts/mouse across 3 images and the mean staining height per mouse determined (n=3-4 mice/group).

### Fecal Lysozyme Activity

Fecal lysozyme activity was measured using an EnzChek Lysozyme assay kit (ThermoFisher Scientific) according to the manufacturer’s instructions. Briefly, fecal homogenates were prepared in PBS (10% wt/vol) and centrifuged at 10,000 x *g* for 10 min. The supernatants were removed and serially diluted in 1x Reaction buffer from the assay kit. Samples and serially diluted lysozyme standards were mixed 1:2 with a FITC (fluorescein isothiocyanate)-conjugated lysozyme substrate and incubated for 30 min at 37°C, after which the fluorescence intensity was measured.

### Statistics

Details of all sample sizes and statistical analyses used in addition to the co-expression analysis are provided in the figure legends. In instances where there was evidence of non-normality (identified by the D’Agostino & Pearson omnibus, Shapiro-Wilk or Kolmogorov–Smirnov normality test) data were analyzed using appropriate non-parametric tests. Values of *P* < 0.05 were accepted as significant. *, *P* < 0.05; **, *P* < 0.01; ***, *P* < 0.001; ****, *P* < 0.0001.

## Results

### Comparison of Global Gene Expression Profiles in the FAE, VE and Ileum of Young and Aged Mice

**Figure 1:**
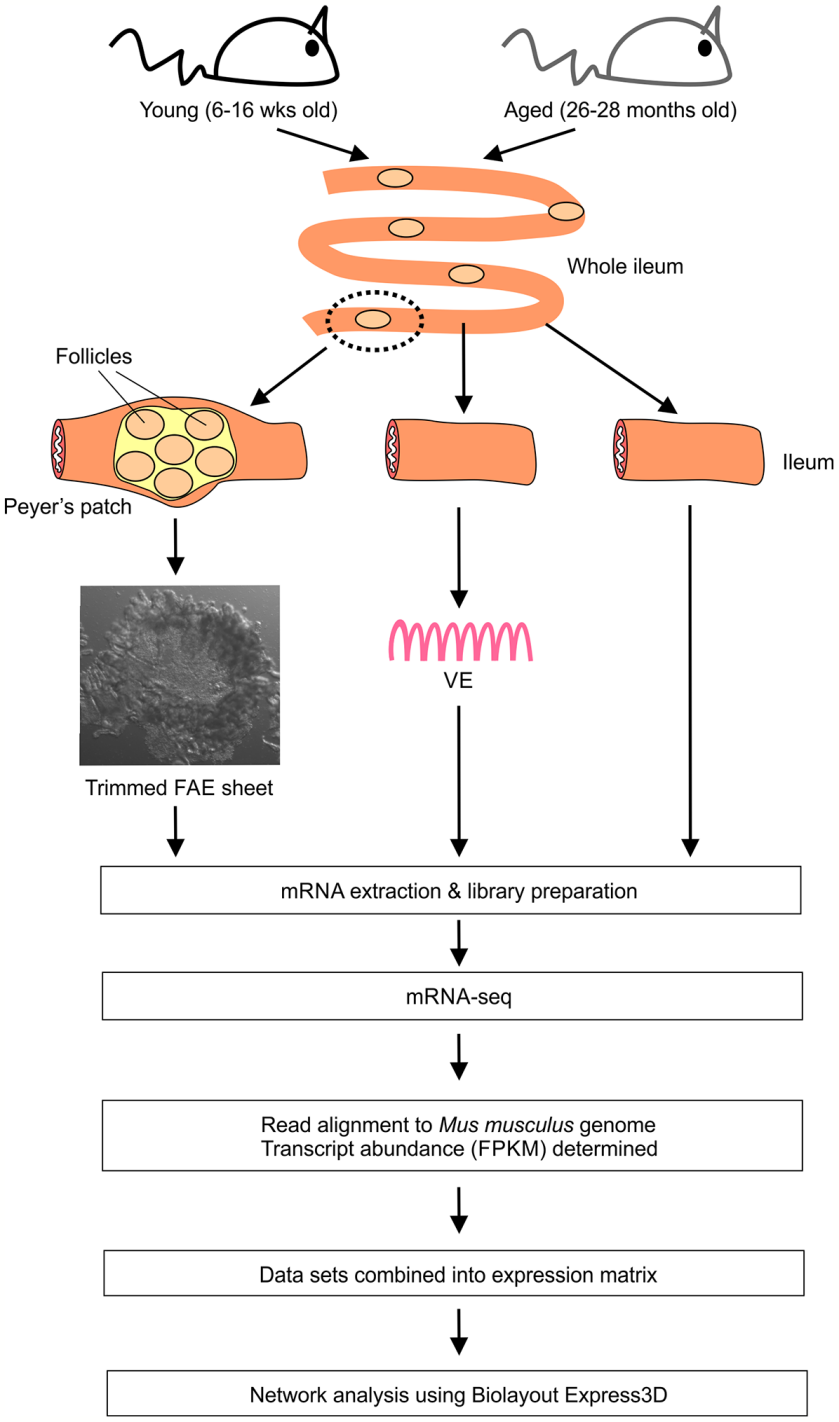
Study design and work-flow.

First we compared the global gene expression correlations across all 24 of the FAE, villous epithelium (VE) and ileum mRNA-seq data sets from young and old mice. Small intestines were removed from groups of young (16 weeks old) and aged (26-28 months old) female C57BL/6J mice, and FAE sheets covering the Peyer’s patches (17–19), conventional VE preparations and 5 mm sections of whole ileum lacking Peyer’s patches collected. Immediately after collection each sample was stored and processed for mRNA-sequencing (**Figure 1**).

**Figure 2:**
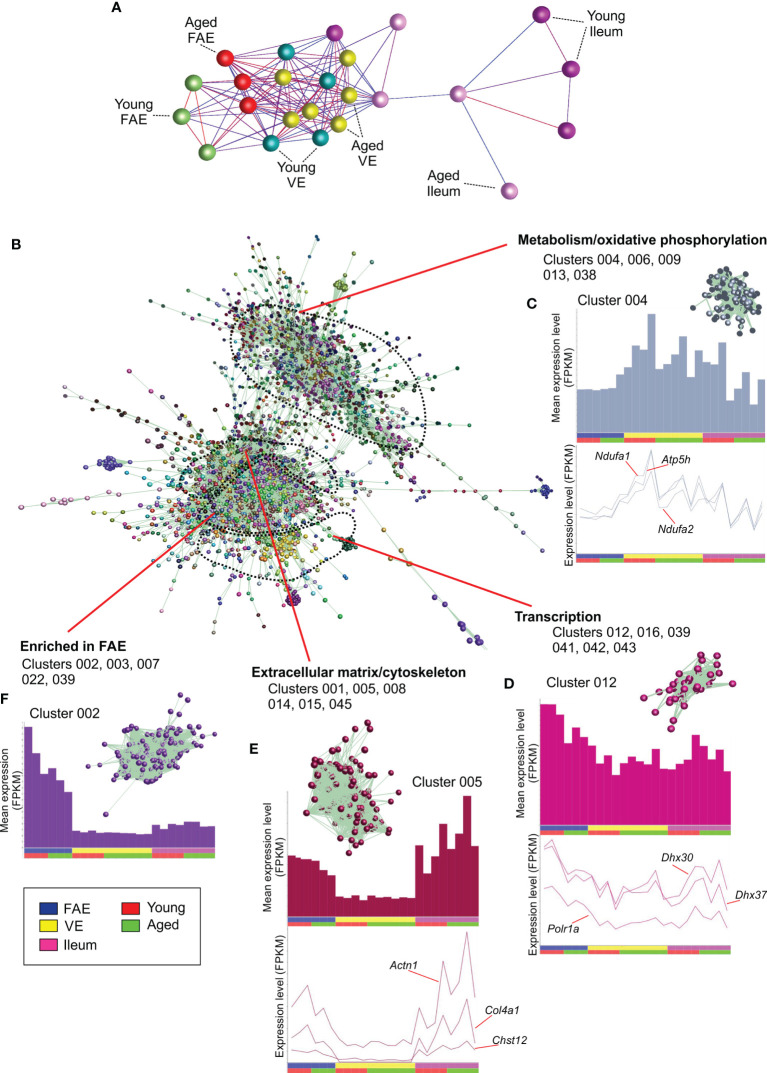
Network co-expression analysis of mRNA-seq data sets from the FAE, VE and ileum from young and aged mice. **(A)** Clustering of samples based on their global gene expression profiles. A graph was created of the sample-to-sample correlations between each data set using Pearson correlation relationships of *r* ≥ 0.86 to define the edges. Nodes represent samples and the edges are colored using a scale based on the strength of the correlation (red, 1.0 - blue, 0.86). **(B)** Main network graph shows gene expression correlations at the transcript (gene) level across each of the 24 data sets using Pearson correlations between genes of *r* ≥ 0.93. Individual gene clusters are indicated by distinct colors, and the locations of specific functional groupings of clusters are indicated. **(C–F)** Upper panels show the average expression profiles (mean FPKM) of the genes in the selected clusters across the 24 data sets, with each bar representing the mean expression level in an individual data set. The insets in the upper panel show the grouping of nodes in the selected cluster from the network graph in **(B)** Lower panels show the expression profiles (FPKM) of selected genes across the 24 data sets.

Next, a mRNA-seq gene expression matrix was created that contained each sample data set, and this was imported into the bioinformatics tool Biolayout *Express*3D (24, 25). A graph was then created of the sample-to-sample correlations between each data set using Pearson correlation relationships of *r* ≥ 0.86 to define the edges. In the sample-to-sample correlation graph the individual mRNA-seq data sets are represented by nodes and the edges show the connections between samples (**Figure 2A**). This analysis showed that the FAE, VE and ileum data sets occupied distinct regions of the graph. Furthermore, within these regions it was evident that the FAE and ileum data sets were generally separated into sub-regions according to age. This implied differences in the global gene expression profiles in the FAE and ileum between young and age mice.

The young and aged VE samples, in contrast, did not display this aged-related separation suggesting that aging did not have a major effect on the global gene expression profile in the VE. This finding is consistent with data from an independent study that reported that aging had only a limited influence on the transcriptome of small intestinal enterocytes (30).

### Identification of Co-Expressed Gene Clusters With Shared Cellular Activities

Next, we compared the gene expression correlations at the gene level across each data set. Genes with very low expression levels of < 0.1 FPKM across all data sets were first removed from the expression matrix. A gene-to-gene Pearson correlation matrix was then calculated for each gene represented across all of the 24 mRNA-seq data sets using a Pearson correlation threshold of *r* ≥ 0.93. The resulting network graph contained 8,995 nodes (genes) connected by 528,285 edges indicating Pearson correlations between genes of *r* ≥ 0.93. After clustering the graph using the MCL algorithm with an inflation value of 2.2, a total of 137 co-expressed gene clusters were obtained containing 8 – 1,423 nodes.

The genes within each co-expression cluster are listed in **Table S1**, and details of the significantly over-represented gene ontologies (GO) and significantly enriched transcription factor (TF) binding site motifs for the genes within the top 50 (largest) clusters are provided in **Table S2**. Each cluster contains groupings of genes that are co-expressed across these data sets in a specific pattern at *r* ≥ 0.93. The mean gene expression profiles of the genes in the top 50 clusters in the network graph are provided in **Figure S1**.

Analysis of the 3D network graph showed that clusters of genes with similar co-expression profiles were generally situated adjacent to each other in distinct regions (**Figure 2B**). Furthermore, analysis of the cluster contents in these regional groupings indicated that the genes within them shared similar cellular activities. Clusters 004, 006, 009, 013 and 038 for example, were situated in the same region of the graph and contained genes that were highly expressed in each data set (**Figure 2C**). The genes in these clusters were significantly enriched in cellular metabolism and oxidative phosphorylation activities (e.g., Cluster 004, GO:0016491 oxidoreductase activity; Cluster 006, GO:0005739 mitochondrion; **Table S2**). Cluster 004 for example contained many genes encoding core components of electron transfer chain complexes (e.g., *Cox7a1*, *Ndufa1*, *Ndufa2*) and mitochondrial ATP synthase (eg: *Atp5h*; **Table S1**). The expression profiles of the individual genes in these clusters matched the cluster mean (**Figure 2C**).

Clusters 012, 016, 039, 041, 042 and 043 were similarly grouped in a distinct region of the graph (**Figure 2D**), and were also expressed highly in each data set. These clusters were significantly enriched in genes involved in transcription (eg: Cluster 012, GO:0003676 nucleic acid binding; Cluster 016, GO:0016070 RNA metabolic process; Cluster 039, GO:0008134 transcription factor binding; **Table S2**), with Cluster 012 containing genes encoding RNA helicases (including *Ddx21*, *Dhx30* & *Dhx37*) and the RNA polymerase I subunit A (*Polr1a*; **Figure 2D**).

Clusters 001, 005, 008, 014, 015 and 045 also occupied a distinct location within the network graph (**Figure 2B**). The mean expression profiles of the genes in these clusters indicated that they were predominantly expressed in the ileum and the FAE data sets (**Figure 2E**). These clusters were significantly enriched in genes encoding components of the cytoskeleton and extracellular matrix (eg: Cluster 001, GO:0005201 extracellular matrix structural constituent; Cluster 005, GO:0008092, cytoskeletal protein binding; Cluster 008, GO:0031012 extracellular matrix). For example, Cluster 005 contained *Actn1*, several chondroitin sulphotransferases (including *Chst12*, *Chst14*, *Chst15*) and collagen encoding genes (eg: *Col4a1*).

Since the genes in the above example co-expression clusters had similar expression profiles and shared similar cellular functions, it is reasonable to hypothesize that each cluster was coordinately regulated by distinct sets of TFs. Indeed, TF binding site motif enrichment analyses in our previous studies of the transcriptomes of distinct immune cell subsets have suggested that the clustering derives from an underlying transcriptional program (26, 31). Here, analysis of significantly enriched TF binding site motifs within the genes of each cluster similarly supported this hypothesis. For example, a large proportion of the 1,424 genes within Cluster 001 were highly significantly enriched in binding site motifs for gut-enriched Krüppel-like factor (GKLF/KLF-4; **Table S2**). This TF plays an important role in maintaining cell homeostasis in intestinal epithelial cells (32, 33).

Comparison of the mean expression profiles of the above clusters suggested similar levels of gene expression in the young and aged data sets. This implied that the expression of the genes encoding these cellular processes was not adversely affected by aging. Furthermore, no cluster profiles were identified in which the mean expression profile in the aged VE data sets was reduced when compared to the young VE data sets. This is consistent with the lack of separation of the young and aged VE data sets in the sample-to-sample graph (**Figure 2B**), and findings from an independent study that reported that aging had only a limited influence on the expression of handful of genes in isolated small intestinal enterocytes (30).

### Reduced Expression of FAE- and M Cell-Specific Genes in Aged Mice

**Figure 3:**
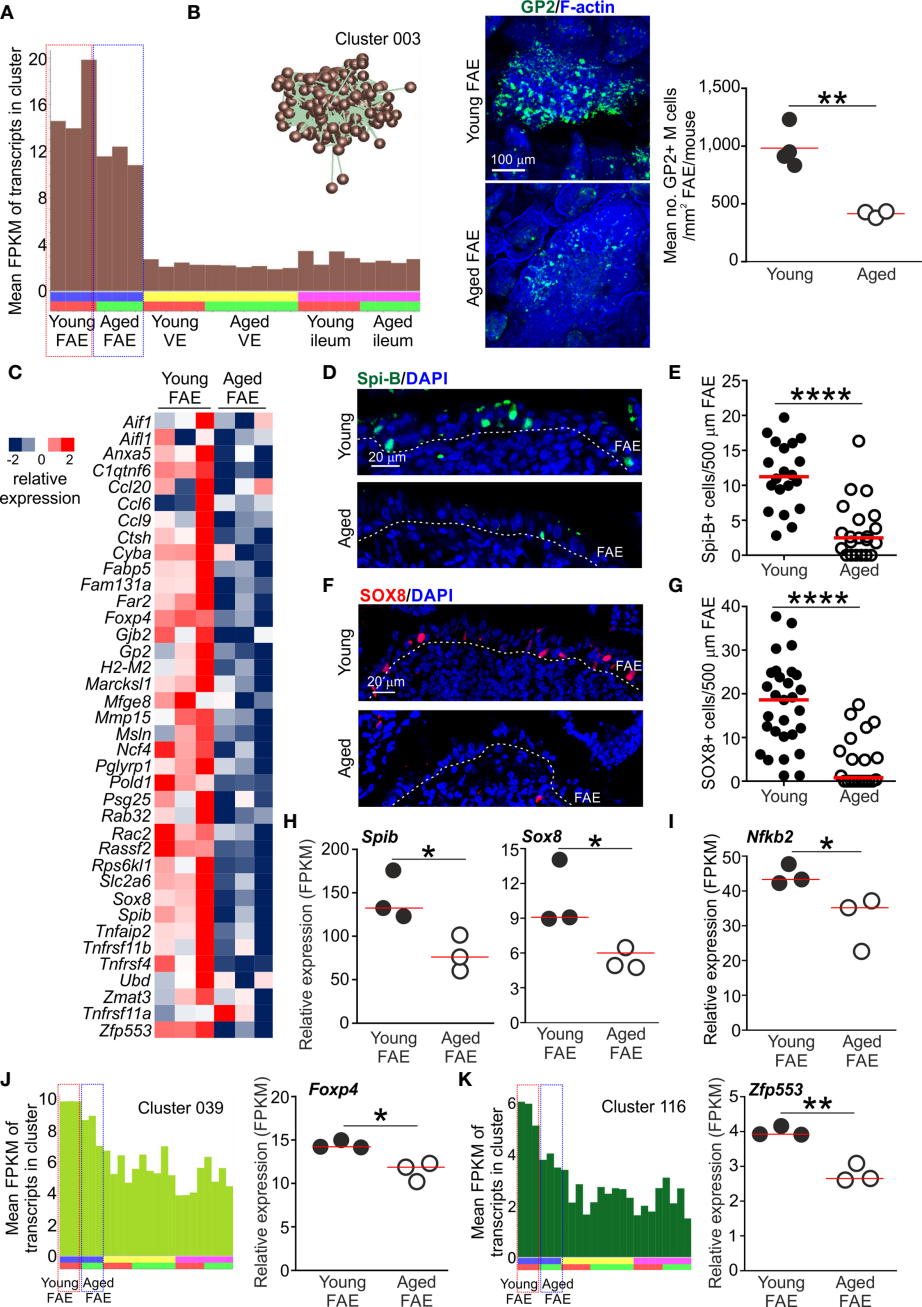
Reduced expression of FAE- and M cell-related genes in the Peyer’s patches of aged mice. **(A)** Mean expression profile (mean FPKM) of the genes in Cluster 003 across the 24 data sets. Inset shows the grouping of nodes in this cluster in the network graph shown in **Figure 2B**. **(B)** Reduced abundance of GP2+ M cells in the FAE of aged mice. Left-hand panels, whole-mount immunostaining of GP2+ M cells (green) in the FAE of young and aged mice. F-actin, blue. Right-hand panel shows mean abundance of GP2+ M cells/mouse (n=3-4/group). Horizontal bar, median. Statistical differences determined by t test. **(C)** Heat map shows relative expression of selected M cell-related genes in the FAE of young and aged mice. **(D)** IHC detection of Spi-B+ cell nuclei in the FAE of Peyer’s patches from young and aged mice. Cell nuclei counterstained with DAPI, blue. Dotted line, basal boundary of the FAE. **(E)** Comparison of frequency of Spi-B+ cell nuclei in the FAE. N=20-21 FAE/group. Horizontal bar, median. Statistical differences determined by Mann-Whitney U test. **(F)** IHC detection of SOX8+ cell nuclei in the FAE of Peyer’s patches from young and aged mice. Cell nuclei counterstained with DAPI, blue. Dotted line, basal boundary of the FAE. **(G)** Comparison of frequency of SOX8+ cell nuclei in the FAE. N=20-29 FAE/group. Horizontal bar, median. Statistical differences determined by Mann-Whitney U test. **(H–I)** Relative expression level (FPKM) of *Spib*, *Sox8* and *Nfkb2*, respectively, in individual FAE mRNA-seq data sets from young and aged mice. **(J)** Left-hand panel shows the mean expression profile (mean FPKM) of the genes in Cluster 039 across the 24 data sets. Right-hand panel shows the relative expression level (FPKM) of *Foxp4* in individual FAE mRNA-seq data sets from young and aged mice. **(K)** Left-hand panel shows the mean expression profiles (mean FPKM) of the genes in Cluster 116 across the 24 data sets. Right-hand panel shows the relative expression level (FPKM) of *Zfp553* in individual FAE mRNA-seq data sets from young and aged mice. **(H–K)** Horizontal bars, median. Statistical differences determined by t test. *P < 0.05; **P < 0.01; ****P < 0.0001.

The mean expression profile of Cluster 003 indicated that the 431 genes within it were selectively expressed in the FAE when compared to the VE and ileum (**Figures 2F** and **3A**). Analysis of the cluster’s contents revealed that it contained many FAE-specific genes such as *Ccl20*, *Kcnj15*, *Mfge8* and *Ubd*. This cluster also contained many genes known to be specifically expressed by M cells in Peyer’s patches, including the differentiating/immature M cell markers *Anxa5* and *Marckls1*, as well as the mature M cell markers *Gp2* (encoding glycoprotein 2, GP2)*, Scg5* (also known as Sgne-1) and *Tnfaip2* (also known as M-Sec) (5, 34–36). A list of all the genes in Cluster 003 is provided in **Table S1**. Many of the genes within this cluster were significantly enriched in functions associated with responses to external stimuli (GO:0009605; **Table S2**), consistent with the role that M cells play in providing immunosurveillance in the intestine (1).

The mean expression profile of the genes in Cluster 003 suggested a general down-regulation of these FAE- and M cell-related genes in the Peyer’s patches of aged mice when compared to young mice (**Figure 3A**). This is consistent with our demonstration that the functional differentiation of M cells is compromised by aging (9, 10). Indeed, immunostaining confirmed a significant reduction in GP2+ mature M cells in aged Peyer’s patches (**Figure 3B**). We therefore compared the expression levels of 37 M cell- and FAE-specific genes based on those identified in series of independent studies (5, 35, 36). This analysis similarly revealed a general down-regulation of most of these genes in the FAE of aged mice when compared to young (**Figure 3C**).

In Peyer’s patches a specific subset of mesenchymal stromal cells situated immediately beneath the FAE produces the cytokine receptor activator of nuclear factor-κB ligand (RANKL) (37, 38). The differentiation and maturation of FAE-associated enterocytes into M cells is critically dependent on RANKL-mediated stimulation from these sub-epithelial mesenchymal stromal cells (7, 37, 38). RANKL-stimulation in enterocytes induces the expression of specific TFs including SOX8 and the E26-transformation-specific (ETS) TF Spi-B. The intrinsic expression of these TFs in RANKL-stimulated enterocytes is essential for the induction and regulation of M cell differentiation (5, 19). Both *Spib* and *Sox8* were represented in Cluster 003 (**Table S1**) and there was significant enrichment for genes containing binding motifs for ETS family TFs in this cluster (**Table S2**). The expression of these TFs was reduced in the FAE of aged mice when compared to young (**Figures 3D–H**), consistent with the reduced abundance of GP2+ mature M cells (**Figure 3B**).

RANKL-RANK signaling is known to activate certain nuclear factor-κB (NF-κB) family TFs which in-turn modulate the expression of multiple target genes (39). There are five members in NF-κB family of TFs: NF-κB1, NF-κB2, RelA, RelB and c-Rel. Amongst these, the RANKL-mediated activation of the non-canonical NF-κB pathway TF family members NF-κB2 and RelB, and canonical NF-κB pathway TF family members NF-κB1 and RelA, is essential for M cell differentiation (18, 40). Activation of c-Rel, in contrast, is dispensable for M cell-differentiation in Peyer’s patches (41). As anticipated, many of the genes in Cluster 003 were significantly enriched with NF-κB family TF binding site motifs, including RelA and RelB (**Table S2**). Furthermore, the gene encoding NF-κB2 (*Nfkb2*) was represented in Cluster 003 and its expression was reduced in the aged FAE when compared to young (**Figure 3I**).

A previous study suggested that the FOXP4 and ZFP553 TFs were specific to M cells in the gut epithelium (36). These TFs were present in separate smaller clusters: *Foxp4*, Cluster 039, 29 genes, GO:0008134 transcription factor binding; *Zfp553*, Cluster 116, 9 genes, GO:0004512 inositol-3-phosphate synthase activity (**Table S1**). The expression of *Foxp4* and *Zfp553* were similarly down-regulated in the aged FAE data sets, consistent with the mean expression profiles of the these clusters (**Figures 3J, K**). Roles for these TFs in M cell-differentiation and function have not been described. However, in the intestine FOXP4 is restricted to epithelial cells during development (42) and its loss in the lung was associated with reduced epithelial cell regeneration and increased goblet cell differentiation (43). Whether loss of FOXP4 similarly affects cell fate in the FAE of Peyer’s patches is not known.

Together, these data show that aging was associated with a general down-regulation in the expression of most FAE-related and M cell-related genes in the Peyer’s patches of aged mice.

### Detection of a B Cell-Related Transcriptomic Signature Within FAE Data Sets

**Figure 4:**
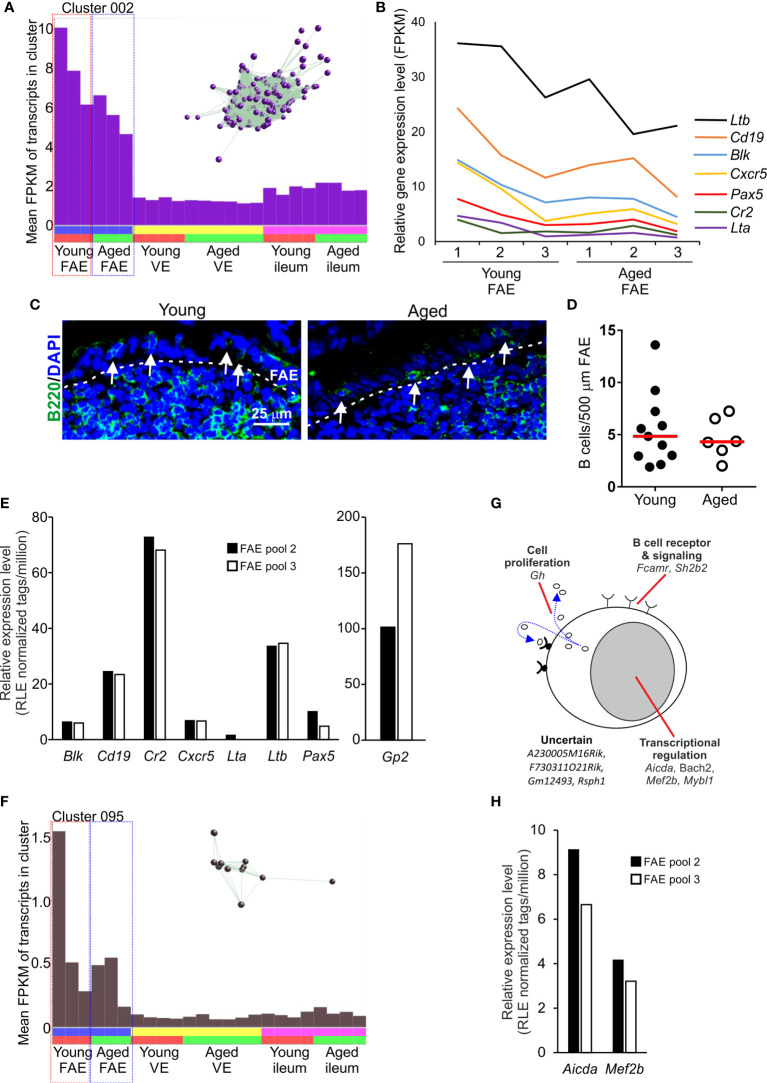
Detection of B cell-related transcriptional signatures in FAE mRNA-seq data sets. **(A)** Mean expression profile (mean FPKM) of the genes in Cluster 002 across the 24 data sets. Inset shows the grouping of nodes in this cluster in the network graph shown in **Figure 2B**. **(B)** Relative expression level (FPKM) of individual B cell-related genes in individual FAE mRNA-seq data sets from young and aged mice. **(C)** IHC detection of B220+ B cells (green, arrows) in the FAE. Cell nuclei counterstained with DAPI, blue. Dotted line indicates the boundary of the FAE. **(D)** Comparison of frequency of B cells (B200+ cells) in the FAE. N=6-12 FAE/group. Horizontal bar, median. Statistical differences determined by t test. **(E)** Expression of mRNA encoding B cell-related genes in independent mRNA deep CAGE sequence data sets derived from FAE sheet preparations (44). **(F)** Mean expression profile (mean FPKM) of the genes in Cluster 095 across the 24 data sets. Inset shows the grouping of nodes in this cluster in the network graph shown in **Figure 2B**. **(G)** Cartoon describing the putative function in B cells of the genes represented in Cluster 095. **(H)** Expression of *Aicda* and *Mef2b* mRNA in independent deep CAGE sequence data sets derived from FAE sheet preparations (44).

The mean expression profile of Cluster 002 also indicated that it contained genes that were predominantly expressed in the FAE data sets (**Figure 4A**). The genes in this cluster were highly significantly enriched in lymphocyte-associated genes (GO:0046649, lymphocyte activation), especially B cells (GO:0042113, B lymphocyte activation). Accordingly, Cluster 002 contained many typical B cell-associated genes including *Blk*, *Cd19*, *Cr2*, *Cxcr5*, *Lta*, *Ltb* and *Pax5* (**Table S1** and **Figure 4B**) implying the presence of small numbers of B cells in the micro-dissected FAE samples. A similar level of expression of these genes was detected across each of the data sets suggesting an equivalent abundance of B cells in FAE samples from young and aged mice (**Figures 4A, B**). These data were consistent with the detection of a similar density of B cells directly within the FAE of young and aged mice by IHC (**Figures 4C, D**). Retrospective analysis of an independent collection of deep CAGE sequence data sets (44) also revealed the presence of B cell-related genes in mRNA from similar FAE sheet preparations (**Figure 4E**).

Consistent with the hypothesis that the genes in these clusters may be coordinately regulated by distinct TFs it was noteworthy that this cluster contained *Pax5*, an important regulator of B cell differentiation (45) (**Figure 4B** and **Table S1**). The ETS TFs PU.1 (encoded by *Spi1*) and Spi-B (encoded by *Spib*) also have essential roles in regulating B cell differentiation (46). Accordingly, there was a highly significant enrichment of genes containing binding site motifs for the ETS, PU.1/Spi1 and Spi-B TFs in this cluster (**Table S2**).

Two studies have described how the close association of B cells with the FAE has consequences for cell differentiation within it, and the induction of intestinal IgA responses. A specific population of FAE-associated B cells are recruited to the FAE in response to production of the chemokine CCL20 and these appear to provide additional factors that stimulate M cell-differentiation (47). Antigen-specific B cells also closely associate with M cells in the FAE of Peyer’s patches. After acquiring antigens from the M cells these B cells rapidly migrate into the Peyer’s patch germinal centre (GC) (3). This M cell to antigen-specific B-cell transporting pathway is important for maintaining GC responses in Peyer’s patches and the induction of intestinal IgA responses. In support of this, Cluster 095 comprised 11 genes that were also predominantly expressed in the FAE data sets and had functions related to B cell activation and function, especially in GC B cells (**Table S1** and **Figures 4F, G**). For example, this cluster contained genes encoding TFs essential for immunoglobulin class-switch recombination and somatic hyper-mutation (*Aicda*, *Bach2*), and gene regulation in GC B cells (*Mef2c*, *Mybl1*). As above, expression of *Aicda* and *Mef2b* was also detected in independent deep CAGE sequence data sets derived from similar FAE sheet preparations (44) (**Figure 4H**).

The detection of these B cell-related transcriptomic signatures in FAE sheet data sets supports the close-association of antigen-specific B cells with M cells (3). These data also highlight the power of this unbiased gene co-expression clustering analysis to identify cell-specific transcriptomic signatures in mRNA data sets from complex tissue samples that contain multiple cell populations (31, 48, 49).

### Reduced Expression of Paneth Cell-Related Genes in the Ileum of Aged Mice

**Figure 5:**
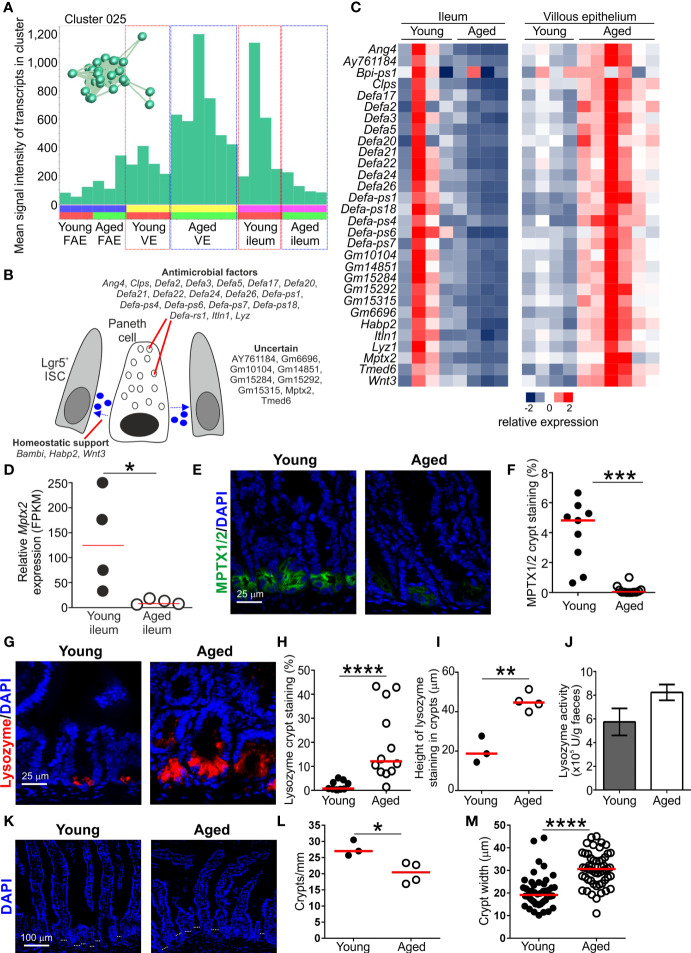
Effect of aging on Paneth cell-related gene expression in the aged intestine. **(A)** Mean expression profile (mean FPKM) of the genes in Cluster 025 across the 24 data sets. Inset shows the grouping of nodes in this cluster in the network graph shown in **Figure 2B**. **(B)** Cartoon describing the putative function in Paneth cells of the genes represented in Cluster 025. **(C)** Heat map shows the relative expression of the Paneth cell-related genes in Cluster 025 in the ileum and villous epithelium mRNA-data sets from young and aged mice. **(D)** Relative expression level (FPKM) of *Mptx2* in individual ileum mRNA-seq data sets from young and aged mice. Horizontal bar, median. Statistical difference determined by t test. **(E)** Comparison of MPTX1/2 immunostaining (green) in Paneth cells in the crypts of young and aged mice. Sections counterstained with DAPI to detect cell nuclei, blue. **(F)** Reduced abundance of MPTX1/2+ immunostaining in the crypts of aged mice. N=9-12 images/group. Horizontal bar, median. Statistical differences determined by Mann-Whitney U test. **(G)** Lysozyme+ immunostaining (red) in Paneth cells in the crypts of young and aged mice. Sections counterstained with DAPI to detect cell nuclei, blue. **(H)** Increased abundance of lysozyme+ immunostaining in the crypts of aged mice. N=9-12 images/group. Horizontal bar, median. Statistical differences determined by Mann-Whitney U test. **(I)** Comparison of the magnitude of the height of the lysozyme+ immunostaining in the crypts of young and aged mice. Data represent the mean of 10-16 crypts/mouse across 3 images/mouse. Horizontal bar, median. Statistical differences determined by t test. **(J)** Equivalent levels of lysozyme activity in feces from young and aged mice. N=5 mice/group. Statistical differences determined by t test. **(K)** Representative sections of small intestines from young and aged mice. Sections stained with DAPI to detect cell nuclei, blue. Dotted lines highlight approximate crypt width. **(L)** Reduced abundance of crypts in the small intestines of aged mice. Data represent the mean number of crypts/mm for each mouse from images containing 4-7 mm intestine. Horizontal bar, median. Statistical differences determined by t test. **(M)** Increased crypt width in the small intestines of aged mice. N=48-53 crypts/group. Horizontal bar, median. Statistical differences determined by t test. *P < 0.05; **P < 0.01; ***P < 0.001; ****P < 0.0001.

Cluster 025 comprised 29 genes that in epithelium of the small intestine are predominantly expressed by Paneth cells at the base of the crypts (**Table S1** and **Figures 5A, B**) (36). This included many genes encoding antimicrobial factors such as angiotensin 4 (*Ang4*), alpha defensins (eg: *Defa2*, *Defa3* etc.), interlectin-1 (*Itln1*) (50) and lysozyme (*Lyz1*). This is consistent with the role of Paneth cells in secreting proteins that help to protect the crypt ISC niche from bacterial infection (51). The *Clps* gene, encoding a pancreatic colipase, was also represented in Cluster 025. High levels of colipase mRNA expression have been detected in Paneth cells by *in situ* hybridisation (52). The function of colipase in Paneth cells has not been determined, but it too may have antimicrobial properties. For example, T cells deficient in colipase have reduced cytotoxicity, and in the pancreas it may be associated with zymogen granule trafficking (52).

Paneth cells also produce homeostatic factors such as Wnt-signaling molecules that provide essential support to Lgr5+ ISC in the crypts (53). The presence of *Wnt3* in this cluster is consistent with Paneth cells providing this homeostatic support. Signaling from bone morphogenic protein (BMP) is required for cell differentiation in the gut epithelium, but also negatively regulates the self-renewal of Lgr5+ ISC. To counter this activity, the expression of BMP antagonists increases towards the crypt ISC niche to help maintain Wnt-signaling in Lgr5+ ISC and inhibit cell differentiation (54). The gene encoding the BMP antagonist BAMBI (BMP and activin membrane‐bound inhibitor) was represented in Cluster 025. Expression of *Bambi* in Paneth cells has not been reported but its production may similarly help to maintain the self-renewal of Lgr5+ ISC. Signaling from hyaluronic acid, in contrast, helps maintain Paneth cell homeostasis in the adult small intestine (55). The presence of *Hapb2* in Cluster 025 encoding a hyaluronic acid binding protein 2 is consistent with this requirement.

Transcription factor binding site motif analysis similarly suggested that the genes expressed within Cluster 025 were coordinately regulated. There was a highly significant enrichment in genes containing binding site motifs for the basic helix–loop–helix (bHLH) TF atonal homolog 1 (Atoh1/Math1; **Table S2**). Expression of Atoh1 is essential for both secretory cell commitment in the small intestine (56), and Paneth cell differentiation (57). There was also significant enrichment of binding site motifs for TCF4 in the genes in Cluster 025 (**Table S2**). Stimulation *via* Wnts in the gut epithelium is transduced through β-catenin and the TF TCF4 (58). This TF plays an important role in regulating gene expression in Paneth cells and the expression of alpha defensins in the embryonic small intestine (59).

Paneth cell function, including the effective provision of homeostatic support to Lgr5+ ISC, is adversely affected by aging and impacts the regenerative ability of the gut epithelium (10, 14, 16). Since M cells derive from Lgr5+ ISC (13), a reduction in provision of Paneth cell-derived homeostatic support in the ISC niche indirectly impedes M cell-differentiation in Peyer’s patches (60). Here, the expression of the Paneth cell-related genes in Cluster 025 was reduced in the ileum of aged mice when compared to young mice (**Figures 5A, C**). For example, Cluster 025 contained *Mptx2* encoding a mucosal pentraxin (61). The function of MPTX2 in Paneth cells is uncertain, but independent single-cell mRNA-sequencing data has suggested this gene is specific to Paneth cells in the epithelium of the small intestine (36). Expression of *Mptx2* was reduced in the ileum of aged mice (**Figure 5D**), and immunostaining showed that the abundance of MPTX1/2 was reduced in the crypts of aged mice when compared to young mice (**Figures 5E, F**).

However, expression of these Paneth cell-related genes was increased in the VE of aged mice when compared to young (**Figures 5A, C**). Thus, although these data support the view that Paneth cell function is reduced in the ileum during aging (14), the increased expression of Paneth cell-related genes in the VE of aged mice implied the displacement of some Paneth cells within the crypts. These data are consistent with findings from independent studies suggesting that aging adversely affects the function and positioning of Paneth cells in the small intestine of aged mice (10, 14, 16). Consistent with this, immunostaining revealed a greater abundance and distribution of Paneth cell-associated lysozyme throughout the crypts of aged mice (**Figures 5G–I**). Despite this, similar levels of lysozyme were detected in the feces of young and aged mice (**Figure 5J**). This implied that ability of Paneth cells to secrete lysozyme was adversely affected in the ileum of aged mice.

To determine how the expression of Paneth cell-related genes could be both reduced in the whole ileum and conversely increased within the epithelium, the number of crypts/mm of small intestine and crypt width was determined (**Figure 5K**). A significant reduction in number of crypts/mm was observed in aged mice (**Figure 5L**), supporting previous studies (14). A concomitant significant increase in crypt width was also observed in aged mice (**Figure 5M**). Therefore although Paneth cell-related gene expression is increased within the epithelium during aging, this is insufficient to offset the changes to the architecture of the aging crypts resulting in reduced total Paneth cell-related gene expression in the ileum. A population of CD90+ fibroblasts has also been identified in the ISC niche that secretes factors that support epithelial cell growth in the small and large intestines (62). Cluster 062 comprised a small cluster of 16 genes associated with the extracellular matrix (GO:0031012, extracellular matrix; **Table S3**), and 7 of these genes were amongst the top 100 genes enriched in CD90+ fibroblasts (62). This implied that the genes in Cluster 062 were expressed in CD90+ fibroblasts (**Table S3**). The expression levels of the genes in cluster 062 and those in the top 100 genes enriched in CD90+ fibroblasts were similar in the ileum of young and aged mice (**Table S3**). The factors produced by CD90+ fibroblasts that support the ISC include the class 3 semaphorins. The expression levels of *Sema3a*, *Sema3b*, *Sema3c* and *Sema3d* were also similar in the ileum of young and aged mice (**Table S3**). These data suggest that aging did not affect the function of CD90+ fibroblasts in the ileum.

### Increased Expression of the SARS-Cov-2 Receptor ACE2 in the Aged FAE

**Figure 6:**
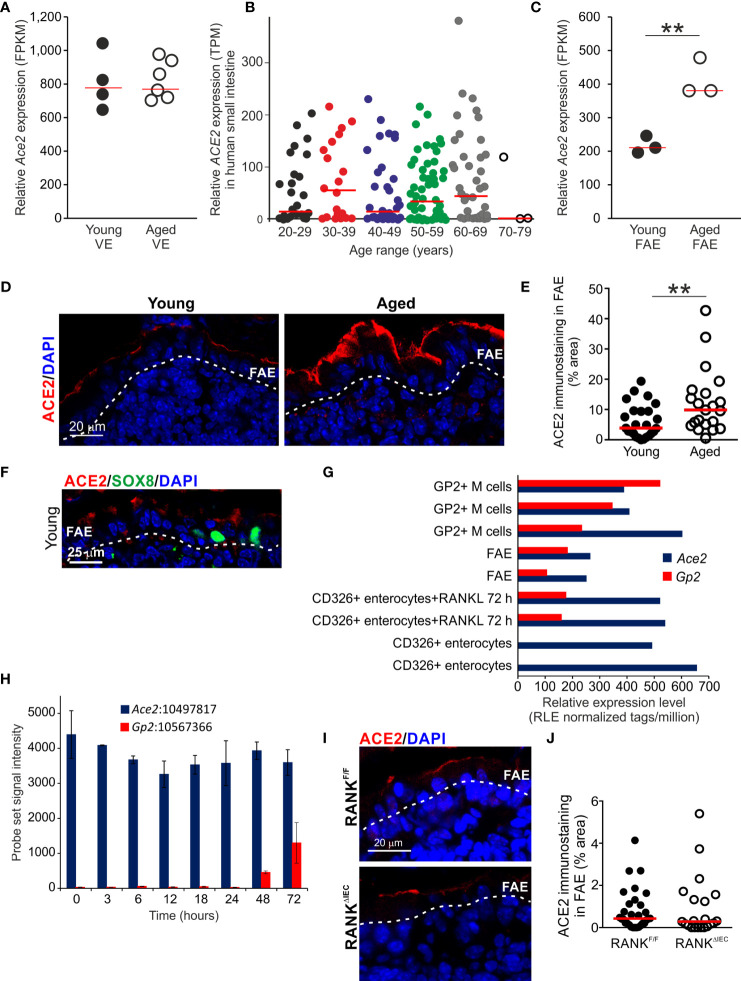
Increased ACE2 expression in the aged FAE. **(A)** Equivalent levels of *Ace2* mRNA expression (FPKM) in the VE of young and aged mice. Each point represents an individual mRNA data set. N=4-6/group. Horizontal bar, median. **(B)** Aging does not affect *ACE2* mRNA expression in the human small intestine. Relative level of *ACE2* expression (TPM) in a large collection of mRNA-seq data sets from human small intestine in the GTEx project (www.gtexportal.org). Each point represents an individual data set. Horizontal bars, median. **(C)** Increased expression of *Ace2* in the FAE of aged mice. Each point represents an individual mRNA-seq data set. N=3/group. Horizontal bar, median. Statistical differences determined by t test. **(D)** |Increased abundance of ACE2+ immunostaining (red) in the FAE of aged mice. Sections counterstained with DAPI to detect cell nuclei, blue. Dotted line indicates the basal boundary of the FAE. **(E)** Analysis of the abundance of ACE+ immunostaining in the FAE of young and aged mice. N=21-24 FAE/group. Horizontal bar, median. Statistical differences determined by Mann-Whitney U test. **(F)** Immunostaining shows ACE2 (red) is expressed on cells throughout the FAE including SOX8+ M cells (green). Sections counterstained with DAPI to detect cell nuclei, blue. Dotted line indicates the basal boundary of the FAE. **(G)** Analysis of published deep CAGE sequence data sets (44) shows that *Ace2* mRNA expression in enterocytes is not dependent on RANKL-stimulation. **(H)** Analysis of published mRNA microarray data sets (5) similarly shows that *Ace2* mRNA expression in enterocytes is not dependent on RANKL-stimulation. (**I)** IHC comparison of ACE2+ immunostaining (red) in the FAE of RANK^ΔIEC^ mice (lower panel) and RANK^F/F^ control mice (upper panel). Sections counterstained with DAPI to detect cell nuclei, blue. Dotted line indicates the basal boundary of the FAE. **(J)** Similar abundance of ACE+ immunostaining in the FAE of RANK^ΔIEC^ mice and RANK^F/F^ control mice. N=21-30 FAE/group. Horizontal bar, median. Statistical differences determined by Mann-Whitney U test, **P < 0.01.

The major receptor for the severe acute respiratory syndrome-coronavirus 2 (SARS-Cov-2) virus is angiotensin-converting enzyme 2 (ACE2) and is expressed in cells throughout the gut epithelium (63). Here, equivalent levels of *Ace2* expression were detected in the VE datasets from young and aged mice (**Figure 6A**). We also compared *ACE2* expression in a large collection of mRNA-seq data sets from human small intestine in the Genotype-Tissue Expression (GTEx) project (www.gtexportal.org). This analysis indicated that age also did not influence *ACE2* expression in the epithelium of the human small intestine (**Figure 6B**, **Table S4**).

However, whereas similar levels of *Ace2* expression were detected in the VE of young and aged mice, its expression was increased approximately two-fold in the FAE of aged mice when compared to young mice (**Figure 6C**). Immunostaining similarly demonstrated that ACE2 protein expression was increased on the apical surfaces of cells throughout the FAE of aged mice (**Figures 6D, E**).

In young mice ACE2 protein was expressed on cells throughout the FAE including SOX8+ M cells (**Figure 6F**). However, although RANKL-stimulation is essential for inducing the differentiation of mature GP2-expressing M cells and the expression of FAE-specific genes such as *Ccl20* (7, 37, 38), analysis of published deep CAGE sequence data sets (**Figure 6G**) (44) and mRNA microarray data sets (**Figure 6H**) (5) showed that *Ace2* expression in the small intestine was not dependent on RANKL-stimulation. Furthermore, IHC analysis showed that the specific ablation of *Tnfsfr11a* (which encodes RANK) in the gut epithelium in RANK^ΔIEC^ mice (7) did not affect ACE2 expression in the FAE when compared to RANK^F/F^ control mice (**Figures 6I, J**). Consistent with the demonstration that RANKL-stimulation is dispensable for *Ace2* expression in the small intestinal epithelium (**Figures 6G–J**), analysis of a published data set of significantly differentially-expressed genes in the FAE of mice deficient in the RANKL-induced TFs SOX8 or Spi-B (19) similarly showed these TFs do not regulate *Ace2* expression in the FAE (**Table S5**). Therefore, increased ACE2 expression in the aged FAE is unlikely to be related to alterations to M cells or RANKL-stimulation.

## Discussion

**Figure 7:**
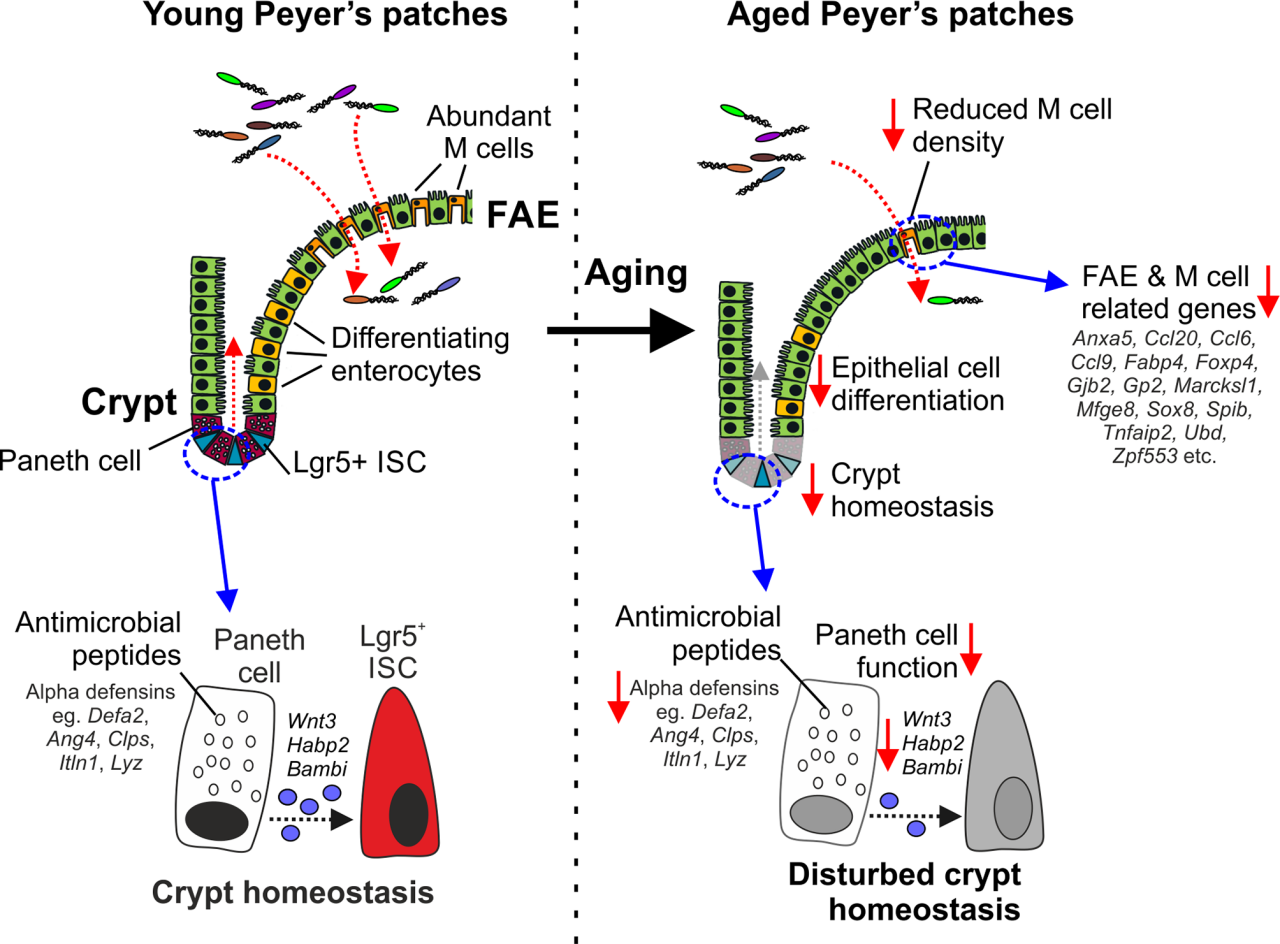
Cartoon describing the impact of aging on Paneth cells and M cells. Paneth cells in the young intestine secrete antimicrobial peptides that help to protect the crypts from bacterial infection. Paneth cells also provide homeostatic support to Lgr5+ intestinal stem cells (ISC). In the aged intestine, the expression of genes encoding antimicrobial peptides and homeostatic support factors is reduced. The disturbed crypt homeostasis in the aged intestine affects epithelial cell differentiation and coincides with reduced M cell differentiation in the FAE, and reduced expression of FAE- and M cell-related genes.

Here we used gene co-expression analysis of mRNA-seq data sets from young and age mice to investigate the effects of aging on epithelial cells in the small intestine and FAE of Peyer’s patches. For example, our analysis identified a cluster of co-expressed genes related to Paneth cells. Expression of these Paneth cell-related genes was reduced in the ileum of aged mice when compared to young mice, consistent with the suggestion that aging adversely affects their function (10, 14, 16) (**Figure 7**). This reduction appeared to be primarily due to changes in the architecture of the intestinal crypts with aging, resulting in fewer crypts, and thus fewer total Paneth cells. However, Paneth cells were increased per crypt as the expression of Paneth cell-related genes was increased in the VE of aged mice, confirmed by lysozyme staining. This increase per crypt was insufficient to offset the reduction in total Paneth cells. These Paneth cells also had an altered distribution with increased numbers higher in the crypt than was found in young mice. Whereas most differentiating/differentiated epithelial cells migrate from the intestinal crypts towards the villous tips. Paneth cells instead home towards the crypt base in response to sources of Wnt signals (59). In mice lacking these positional cues, such as those with intestine-specific Wnt-signaling deficiency, the positioning of Paneth cells is similarly disturbed with some detected higher up in the crypt (59). The gene encoding Wnt3 was represented in this cluster of Paneth cell-related genes, and has similarly been shown to be reduced in aged Paneth cells (14). Thus, the effects of aging on Wnt signals such as *Wnt3* may contribute to their disturbed displacement in the aging crypts of the small intestine.

Aging also adversely affects the maturation and function of M cells in Peyer’s patches (9, 10). We show here that this deficit in aged Peyer’s patches is accompanied by a general down-regulation in the expression of most FAE- and M cell-related genes. This included the reduced expression of key TFs including SOX8, Spi-B and NF-κB2 known to be essential for M cell differentiation (5, 18, 40). M cells, like other gut epithelial cells, derive from Lgr5+ ISC (64). We have shown that disturbances to Lgr5+ ISC caused by reduced provision of homeostatic support from Paneth cells can indirectly affect the functional maturation of M cells in Peyer’s patches (60). Paneth cell function is also diminished in the intestines of aged mice, negatively affecting Lgr5+ ISC and the regenerative capacity of the gut epithelium (14). These effects of aging on Paneth cells and Lgr5+ ISC similarly appear to impede M cell differentiation (10) (**Figure 7**). However, we have shown that restoration of Paneth cell function may represent a novel means to improve the status of the ISC niche, enhance M cell differentiation in the aging intestine and enhance the efficacy of mucosal vaccinations in the elderly (10).

Infection with the SARS-Cov-2 coronavirus in humans predominantly targets the respiratory tract causing the disease COVID-19 (65). However, enterocytes also express ACE2 which acts as an entry receptor for the SARS-Cov-2 virus (63) and can support virus replication (66, 67). Patients with COVID-19 can present with enteric symptoms in addition to respiratory signs, with 4% of patients in one UK study displaying enteric symptoms only (68). Aging is a substantial risk factor for susceptibility to SARS-Cov-2 infection with the highest rate of severe COVID-19 disease and mortality in individuals ≥ 80 years old (68), with children experiencing much milder symptoms if any, and much reduced mortality. Multiple factors may contribute to this age-related susceptibility to severe COVID-19, especially the impact of immunosenescence on the induction and regulation of effective anti-SARS-Cov-2 virus immune responses. Age related differences in ACE2 expression in target tissues may also contribute to the contrasting susceptibility of children and elderly adults to severe COVID-19 disease. For example, one study suggested that ACE2 expression was higher in the lower respiratory tract epithelium of elderly human males (69). Our finding that ACE2 expression was increased in the FAE of aged mice raises the possibility that this may increase the susceptibility of Peyer’s patches in the elderly to infection with the SARS-Cov-2 virus. An aging-related increase in *ACE2* mRNA expression was not observed in the human small intestine mRNA-seq data sets, but these will have lacked significant FAE content. Analysis of ACE2 immunostaining in Peyer’s patches will be necessary to determine whether its expression is similarly upregulated in the FAE of elderly humans.

A genome-wide association meta-analysis of approximately 49,000 COVID-19 patients identified a significant association between the *FOXP4* locus and severe COVID-19 disease (70). How alterations to *FOXP4* expression may influence disease severity is not known, but it is interesting to note that in our study expression of *Foxp4* was reduced in the FAE of aged mice.

The M cell-mediated transfer of viruses such as norovirus and reovirus into Peyer’s patches has been shown to be essential for efficient infection in mice (71, 72). Intestinal M cells express a range of surface molecules including GP2 (4), cellular prion protein (PrP^C^) (73) and uromodulin (UMOD) (74) that can act as uptake receptors for certain microorganisms. Further research is required to determine whether ACE2 acts an uptake receptor for SARS-Cov-2 in M cells to mediate infection of Peyer’s patches. The use of certain viral proteins to target vaccine antigens to M cells might also represent a novel means to enhance antigen-specific mucosal immunity against certain pathogens (75).

## Data Availability Statement

The datasets generated for this study can be found in the Gene Expression Omnibus data base (GEO; www.ncbi.nlm.nih.gov/geo) *via* the following accession code and link: GSE182252.

## Ethics Statement

The animal study was reviewed and approved by The Roslin Institute’s Ethical Review Committee.

## Author Contributions

NM conceived the study and obtained funding. DD and NM designed the study. DD, BS, and NM performed the experiments. BS contributed to the bioinformatics analyses. DD and NM wrote the manuscript. All authors contributed to the article and approved the submitted version.

## Funding

This work was supported by funding from the Biotechnology and Biological Sciences Research Council (grant numbers BB/M024288/1; BBS/E/D/20002174, BBS/E/D/30002276 & BB/S019294/1) and Medical Research Council (grant number MR/S000763/1).

## Conflict of Interest

The authors declare that the research was conducted in the absence of any commercial or financial relationships that could be construed as a potential conflict of interest.

## Publisher’s Note

All claims expressed in this article are solely those of the authors and do not necessarily represent those of their affiliated organizations, or those of the publisher, the editors and the reviewers. Any product that may be evaluated in this article, or claim that may be made by its manufacturer, is not guaranteed or endorsed by the publisher.

## Supplementary Material

Supplementary Table 1 Contents of the genes all the co-expressed gene clusters derived from the analysis in this study. Cluster functional enrichment analysis at Pearson r = 0.93, MCL 2.2.

Supplementary Table 2 Enrichment analyses of the genes in clusters 1-50 and 116. Each tab contains details of the significantly over-represented gene ontologies (GO) and significantly enriched TF binding site motifs for the genes in the specified cluster.

Supplementary Table 3 Genes represented in Cluster 062 and comparison with the top 100 genes enriched in CD90+ fibroblasts in an independent study (62).

Supplementary Table 4 Statistical comparisons of *ACE2* expression in data sets from human small intestine in the Genotype-Tissue Expression (GTEx) project (www.gtexportal.org).

Supplementary Table 5
*Sox8*-deficiency and *Spib*-deficiency do not affect *Ace2* expression in the FAE. Analysis of differentially-expressed genes in the FAE of mice deficient in *Spib* or *Sox8* in an independent study (19) show deficiency in these TFs does not affect *Ace2* expression in the FAE (19).

Supplementary Figure 1 The mean gene expression profiles of the genes in the top 50 clusters in the network graph.
