# Supplementary material for: Aging-Related Impairments to M Cells in Peyer’s Patches Coincide With Disturbances to Paneth Cells
Source: Front Immunol. 2021 Dec 6;12:761949. doi: 10.3389/fimmu.2021.761949 (PMC8687451; doi:10.3389/fimmu.2021.761949)
Supplement: Supplementary Figure 1 — The mean gene expression profiles of the genes in the top 50 clusters in the network graph. [file DataSheet_1.pdf]

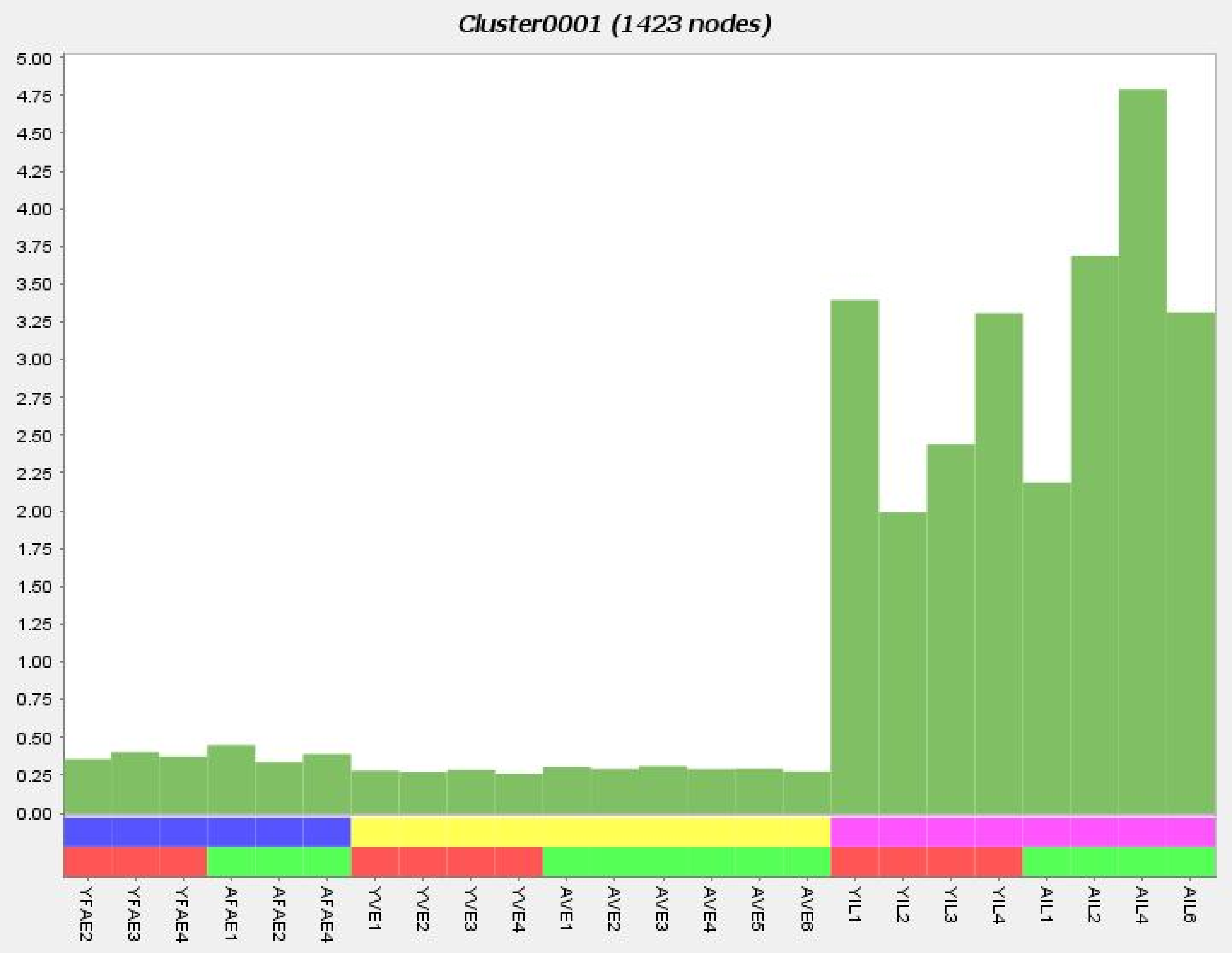
Cluster0001 (1423 nodes)
5.00
4.75
4.50
4.25
4.00
3.75
3.50
3.25
3.00
2.75
2.50
2.25
2.00
1.75
1.50
1.25
1.00
0.75
0.50
0.25
0.00
YFAE2
YFAE3
YFAE4
AFAE1
AFAE2
AFAE4
YVE1
YVE2
YVE3
YVE4
AVE1
AVE2
AVE3
AVE4
AVE5
AVE6
YIL1
YIL2
YIL3
YIL4
AIL1
AIL2
AIL4
AIL6

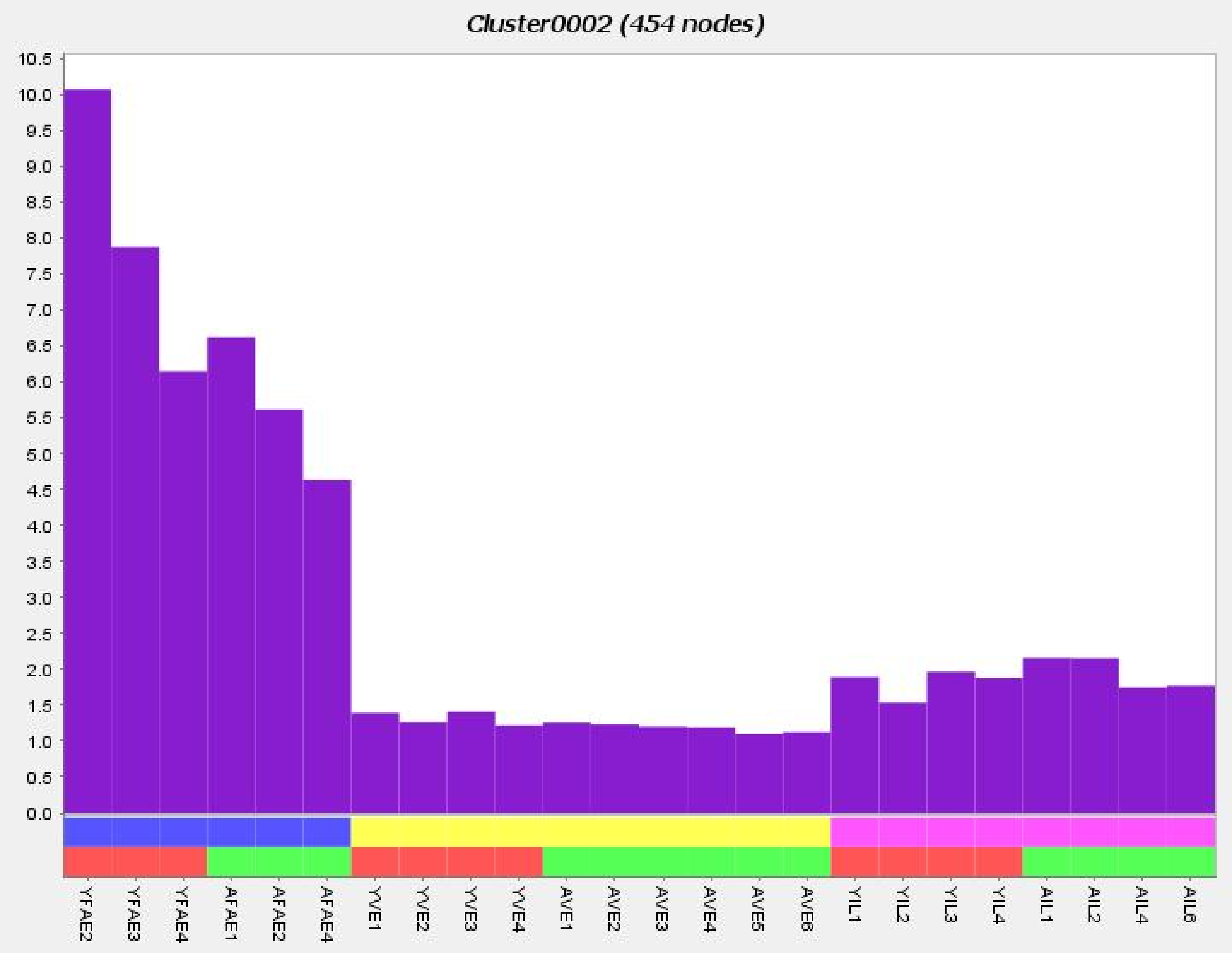
Cluster0002 (454 nodes)
10.5
10.0
9.5
9.0
8.5
8.0
7.5
7.0
6.5
6.0
5.5
5.0
4.5
4.0
3.5
3.0
2.5
2.0
1.5
1.0
0.5
0.0
YFAE2
YFAE3
YFAE4
AFAE1
AFAE2
AFAE4
YVE1
YVE2
YVE3
YVE4
AVE1
AVE2
AVE3
AVE4
AVE5
AVE6
YIL1
YIL2
YIL3
YIL4
AIL1
AIL2
AIL4
AIL6

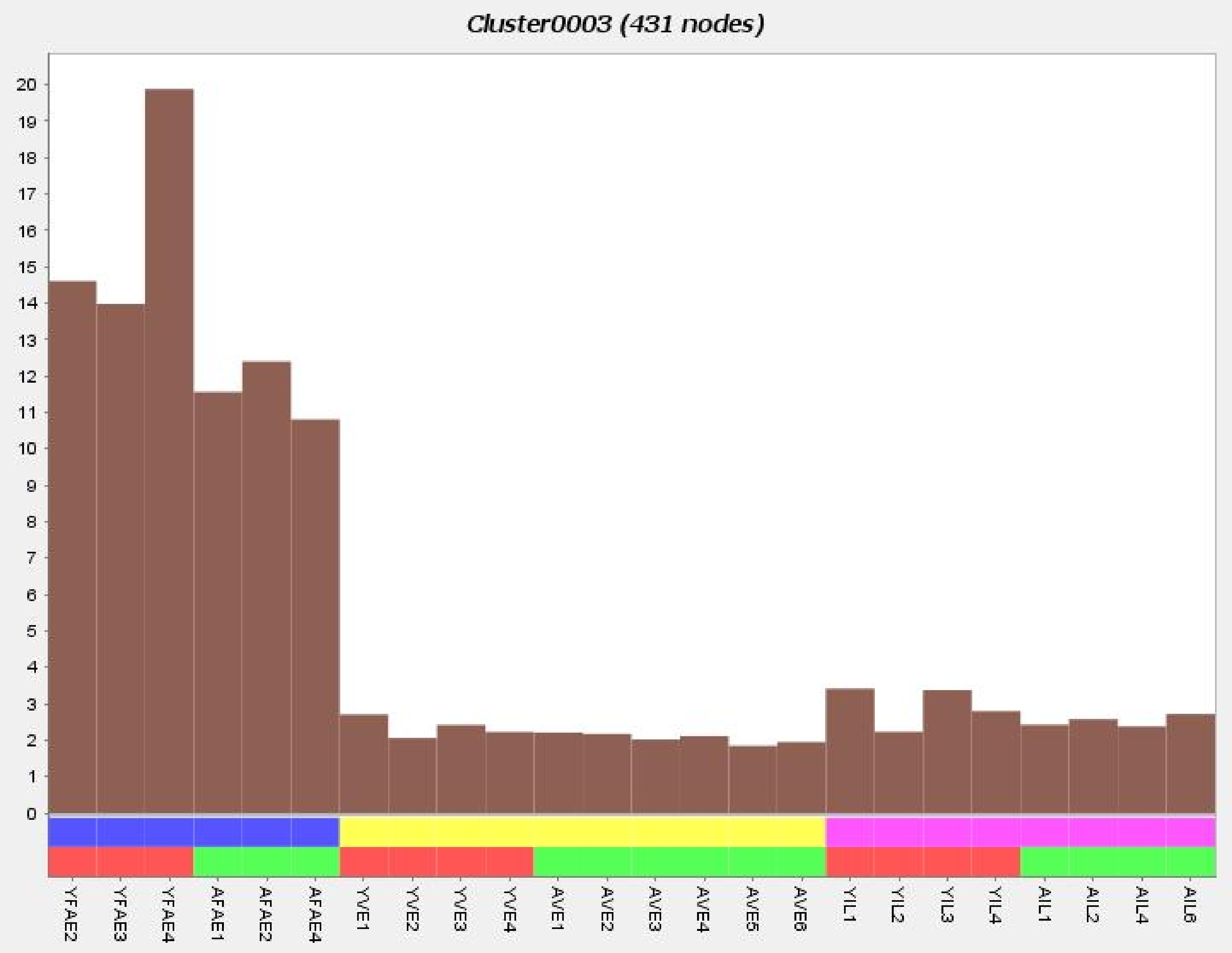

Cluster0003 (431 nodes)
0
1
2
3
4
5
6
7
8
9
10
11
12
13
14
15
16
17
18
19
20
YFAE2
YFAE3
YFAE4
AFAE1
AFAE2
AFAE4
YVE1
YVE2
YVE3
YVE4
AVE1
AVE2
AVE3
AVE4
AVE5
AVE6
YIL1
YIL2
YIL3
YIL4
AIL1
AIL2
AIL4
AIL6

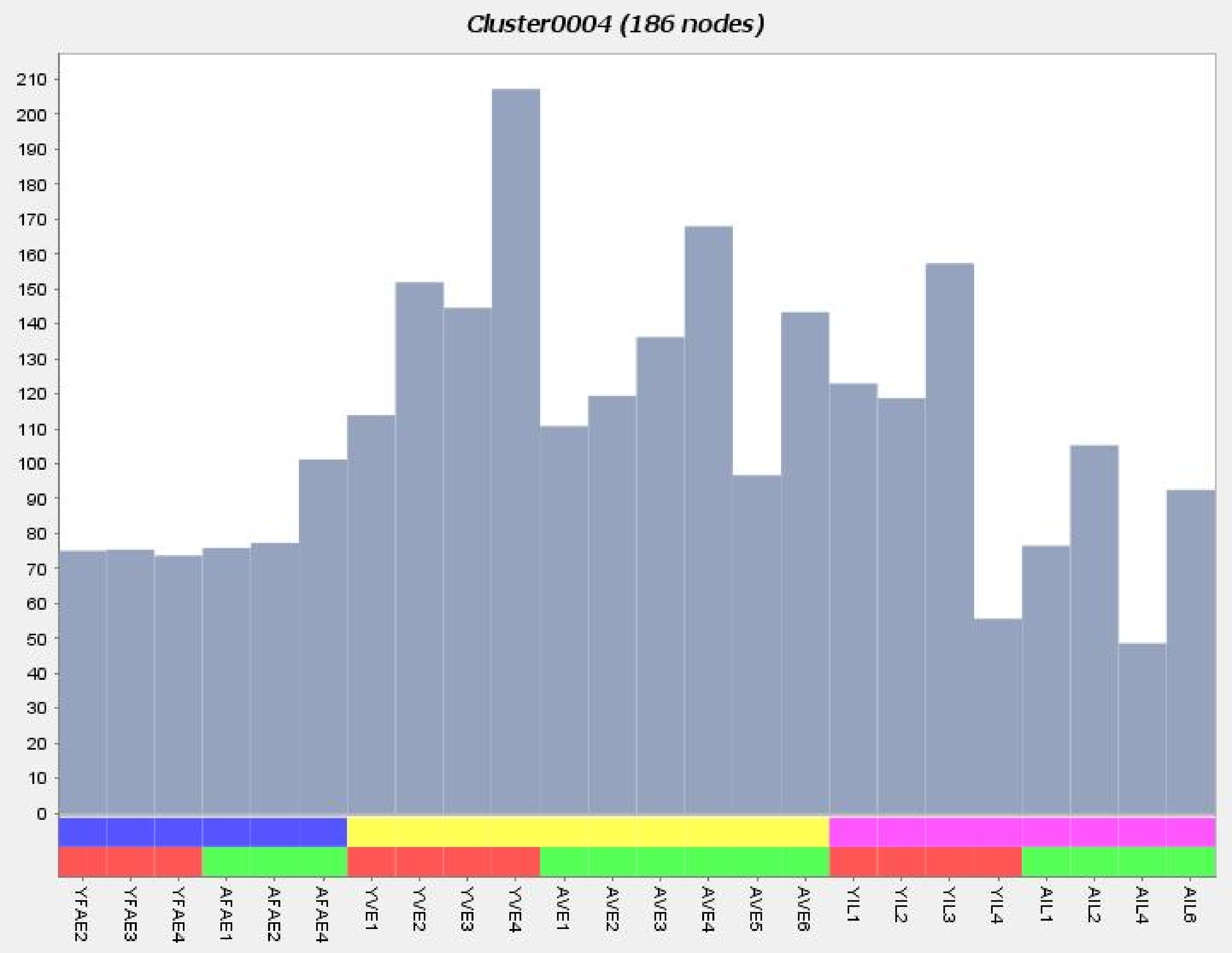

Cluster0004 (186 nodes)
210
200
190
180
170
160
150
140
130
120
110
100
90
80
70
60
50
40
30
20
10
0
YFAE2
YFAE3
YFAE4
AFAE1
AFAE2
AFAE4
YVE1
YVE2
YVE3
YVE4
AVE1
AVE2
AVE3
AVE4
AVE5
AVE6
YIL1
YIL2
YIL3
YIL4
AIL1
AIL2
AIL4
AIL6

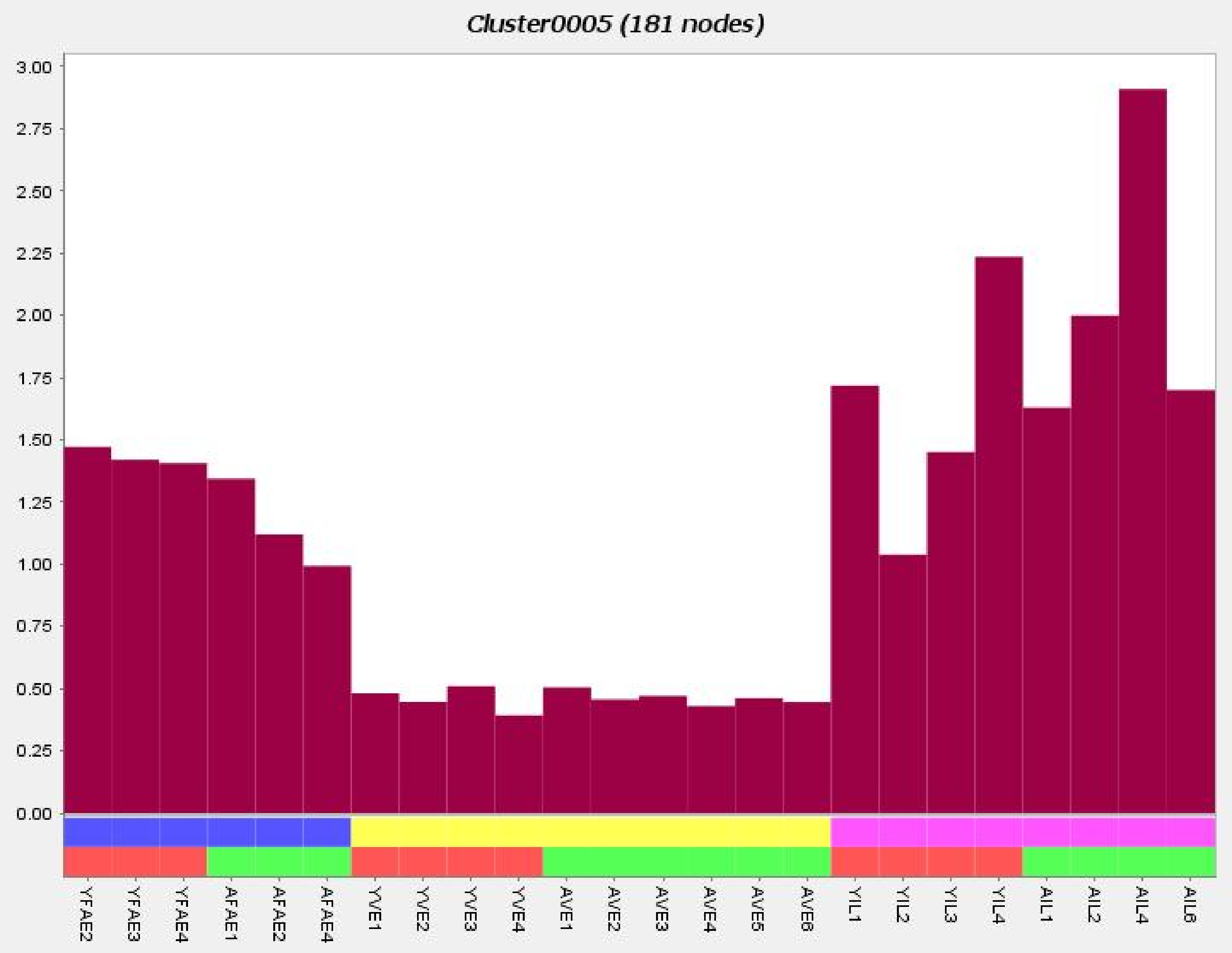
Cluster0005 (181 nodes)
3.00
2.75
2.50
2.25
2.00
1.75
1.50
1.25
1.00
0.75
0.50
0.25
0.00
YFAE2
YFAE3
YFAE4
AFAE1
AFAE2
AFAE4
YVE1
YVE2
YVE3
YVE4
AVE1
AVE2
AVE3
AVE4
AVE5
AVE6
YIL1
YIL2
YIL3
YIL4
AIL1
AIL2
AIL4
AIL6

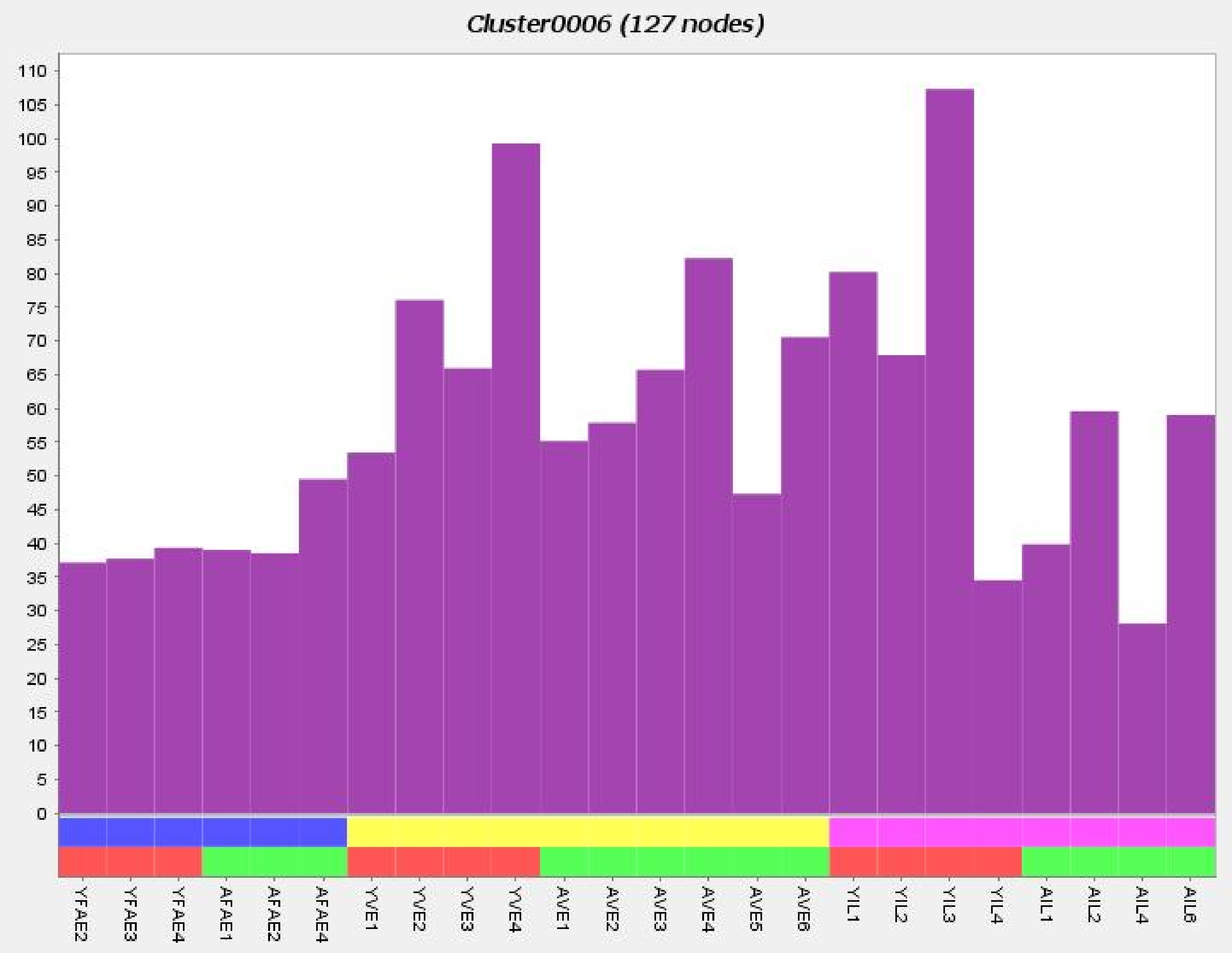
Cluster0006 (127 nodes)
110
105
100
95
90
85
80
75
70
65
60
55
50
45
40
35
30
25
20
15
10
5
0
YFAE2
YFAE3
YFAE4
AFAE1
AFAE2
AFAE4
YVE1
YVE2
YVE3
YVE4
AVE1
AVE2
AVE3
AVE4
AVE5
AVE6
YIL1
YIL2
YIL3
YIL4
AIL1
AIL2
AIL4
AIL6

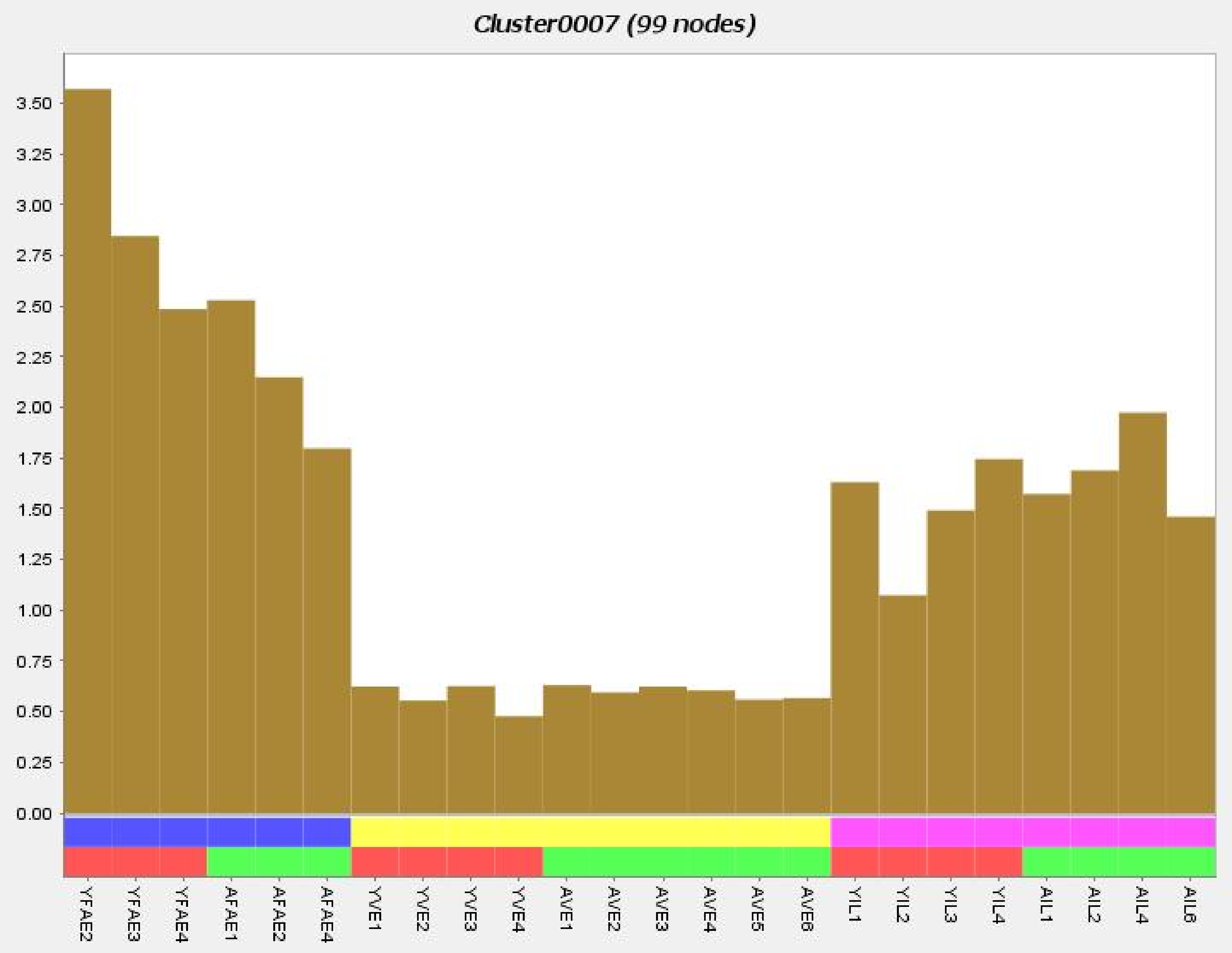

Cluster0007 (99 nodes)
3.50
3.25
3.00
2.75
2.50
2.25
2.00
1.75
1.50
1.25
1.00
0.75
0.50
0.25
0.00
YFAE2
YFAE3
YFAE4
AFAE1
AFAE2
AFAE4
YVE1
YVE2
YVE3
YVE4
AVE1
AVE2
AVE3
AVE4
AVE5
AVE6
YIL1
YIL2
YIL3
YIL4
AIL1
AIL2
AIL4
AIL6

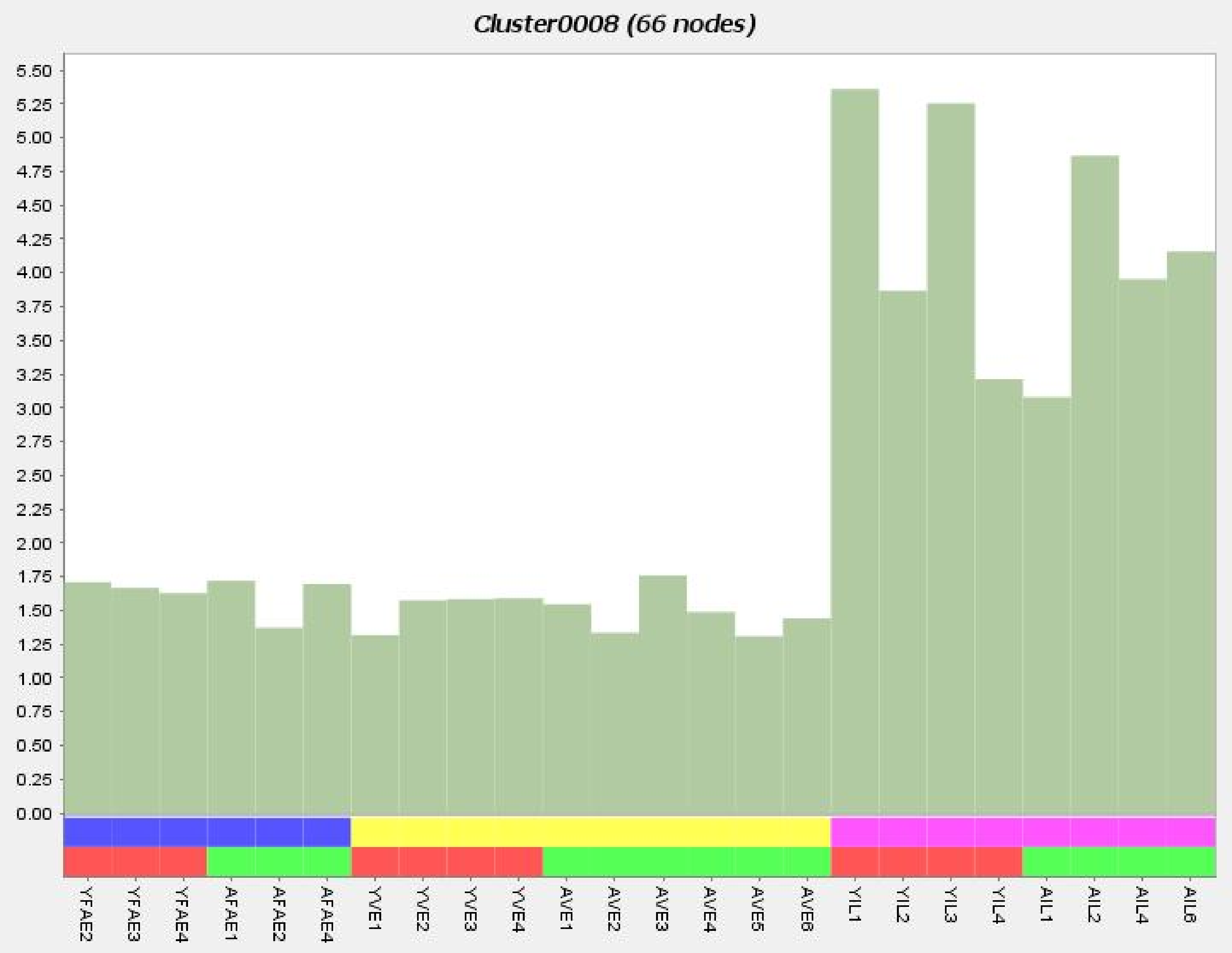
Cluster0008 (66 nodes)
5.50
5.25
5.00
4.75
4.50
4.25
4.00
3.75
3.50
3.25
3.00
2.75
2.50
2.25
2.00
1.75
1.50
1.25
1.00
0.75
0.50
0.25
0.00
YFAE2
YFAE3
YFAE4
AFAE1
AFAE2
AFAE4
YVE1
YVE2
YVE3
YVE4
AVE1
AVE2
AVE3
AVE4
AVE5
AVE6
YIL1
YIL2
YIL3
YIL4
AIL1
AIL2
AIL4
AIL6

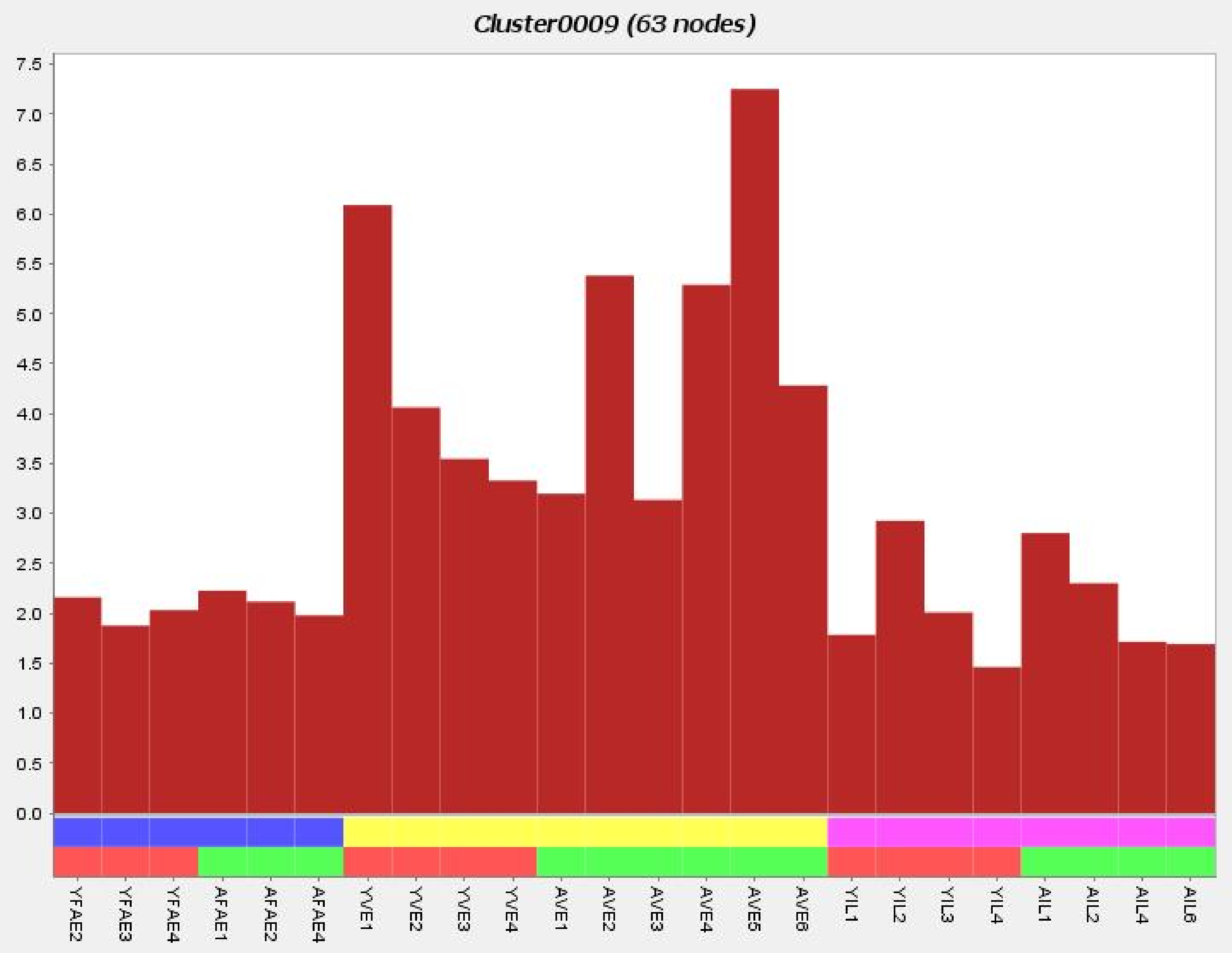
Cluster0009 (63 nodes)
7.5
7.0
6.5
6.0
5.5
5.0
4.5
4.0
3.5
3.0
2.5
2.0
1.5
1.0
0.5
0.0
YFAE2
YFAE3
YFAE4
AFAE1
AFAE2
AFAE4
YVE1
YVE2
YVE3
YVE4
AVE1
AVE2
AVE3
AVE4
AVE5
AVE6
YIL1
YIL2
YIL3
YIL4
AIL1
AIL2
AIL4
AIL6

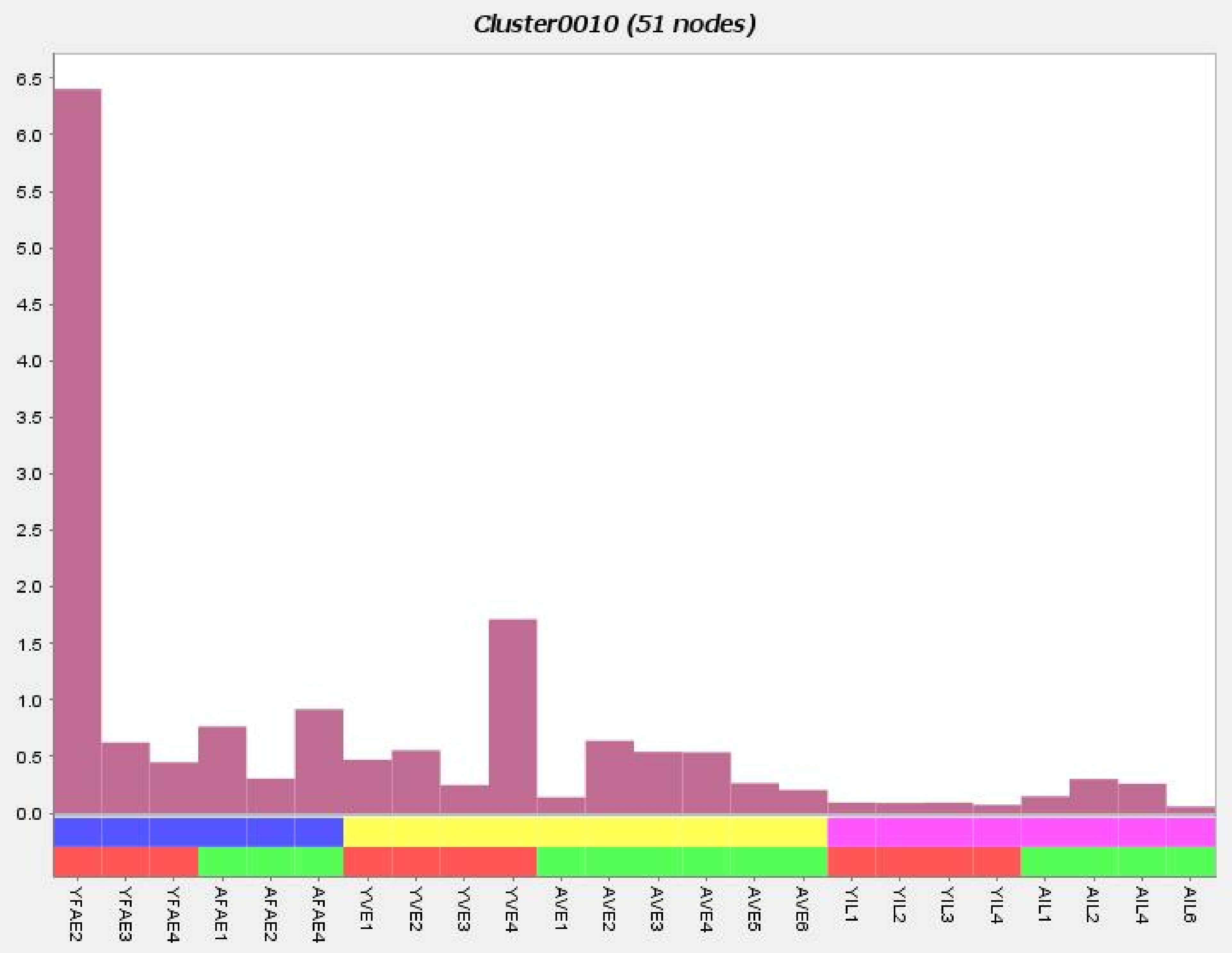

Cluster0010 (51 nodes)
6.5
6.0
5.5
5.0
4.5
4.0
3.5
3.0
2.5
2.0
1.5
1.0
0.5
0.0
YFAE2
YFAE3
YFAE4
AFAE1
AFAE2
AFAE4
YVE1
YVE2
YVE3
YVE4
AVE1
AVE2
AVE3
AVE4
AVE5
AVE6
YIL1
YIL2
YIL3
YIL4
AIL1
AIL2
AIL4
AIL6

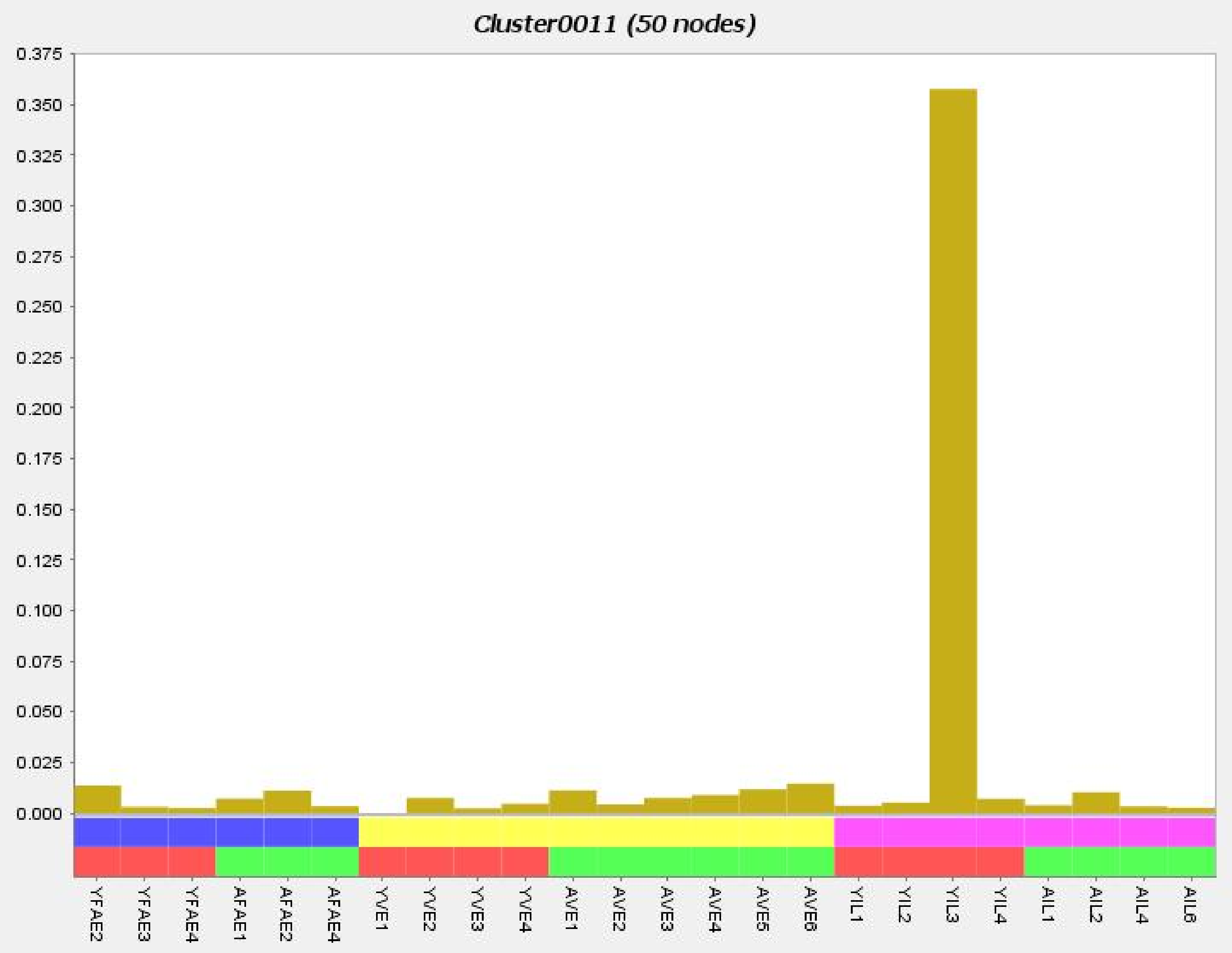
Cluster0011 (50 nodes)
0.375
0.350
0.325
0.300
0.275
0.250
0.225
0.200
0.175
0.150
0.125
0.100
0.075
0.050
0.025
0.000
YFAE2
YFAE3
YFAE4
AFAE1
AFAE2
AFAE4
YVE1
YVE2
YVE3
YVE4
AVE1
AVE2
AVE3
AVE4
AVE5
AVE6
YIL1
YIL2
YIL3
YIL4
AIL1
AIL2
AIL4
AIL6

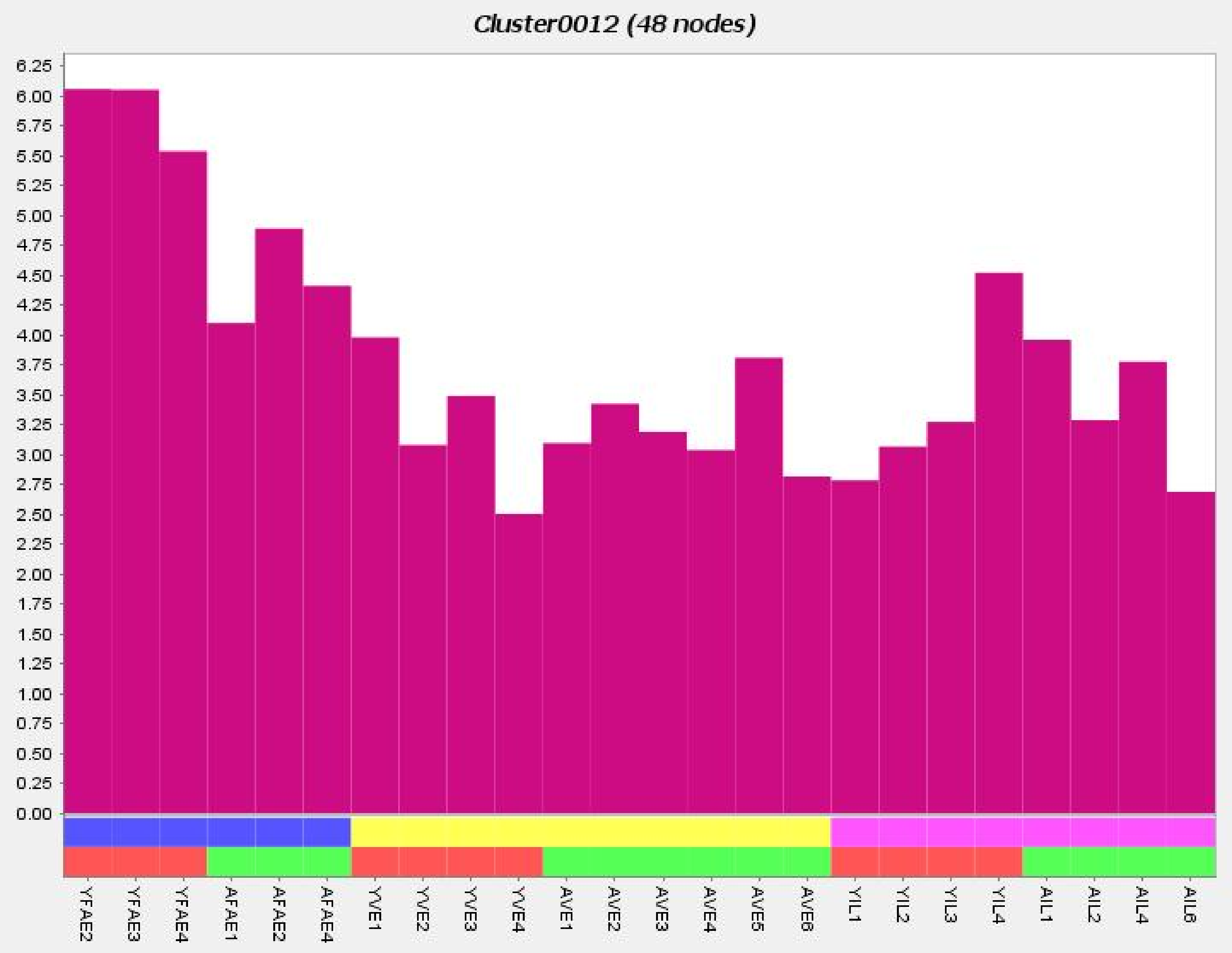
Cluster0012 (48 nodes)
6.25
6.00
5.75
5.50
5.25
5.00
4.75
4.50
4.25
4.00
3.75
3.50
3.25
3.00
2.75
2.50
2.25
2.00
1.75
1.50
1.25
1.00
0.75
0.50
0.25
0.00
YFAE2
YFAE3
YFAE4
AFAE1
AFAE2
AFAE4
YVE1
YVE2
YVE3
YVE4
AVE1
AVE2
AVE3
AVE4
AVE5
AVE6
YIL1
YIL2
YIL3
YIL4
AIL1
AIL2
AIL4
AIL6

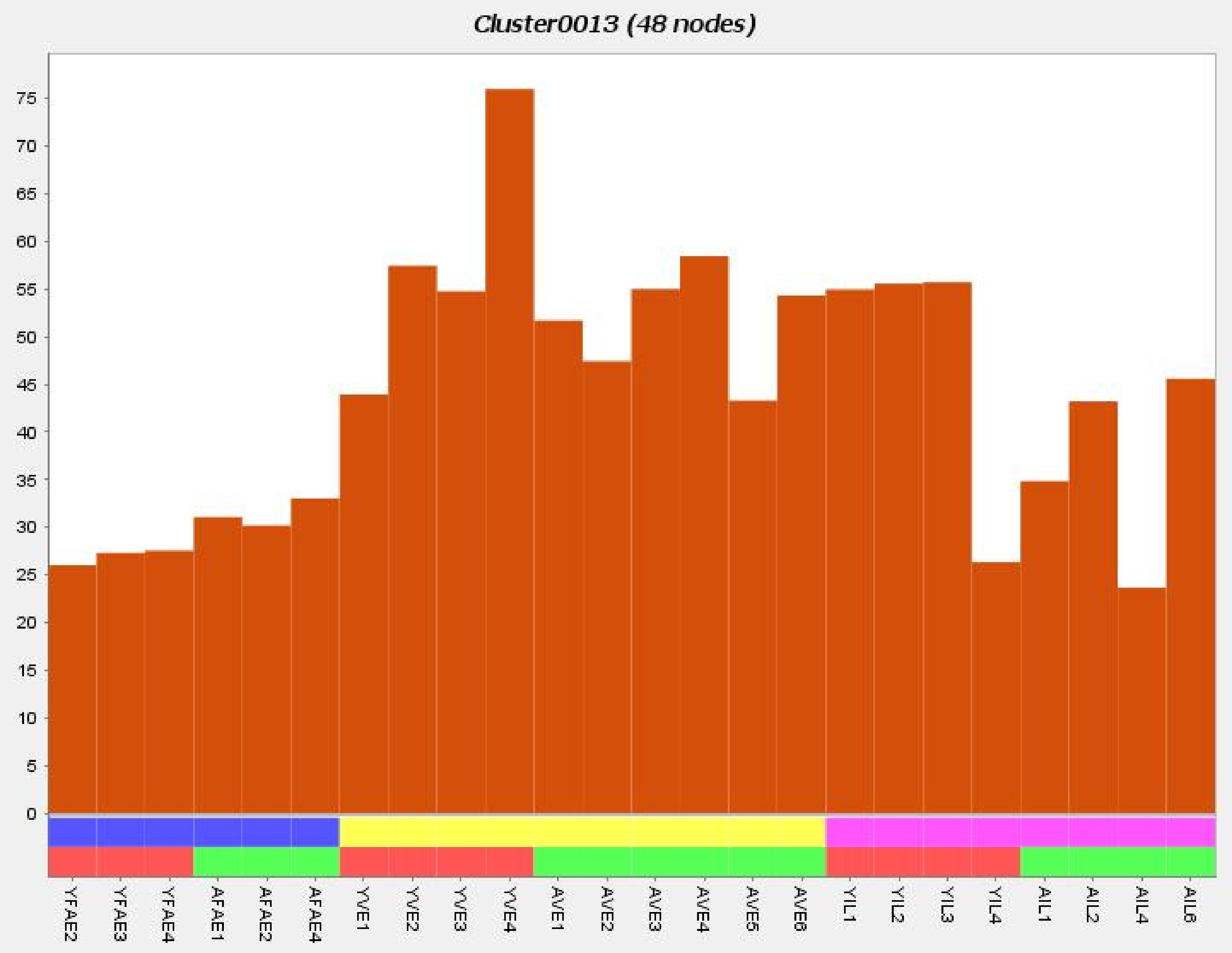
Cluster0013 (48 nodes)
75
70
65
60
55
50
45
40
35
30
25
20
15
10
5
0
YFAE2
YFAE3
YFAE4
AFAE1
AFAE2
AFAE4
YVE1
YVE2
YVE3
YVE4
AVE1
AVE2
AVE3
AVE4
AVE5
AVE6
YIL1
YIL2
YIL3
YIL4
AIL1
AIL2
AIL4
AIL6

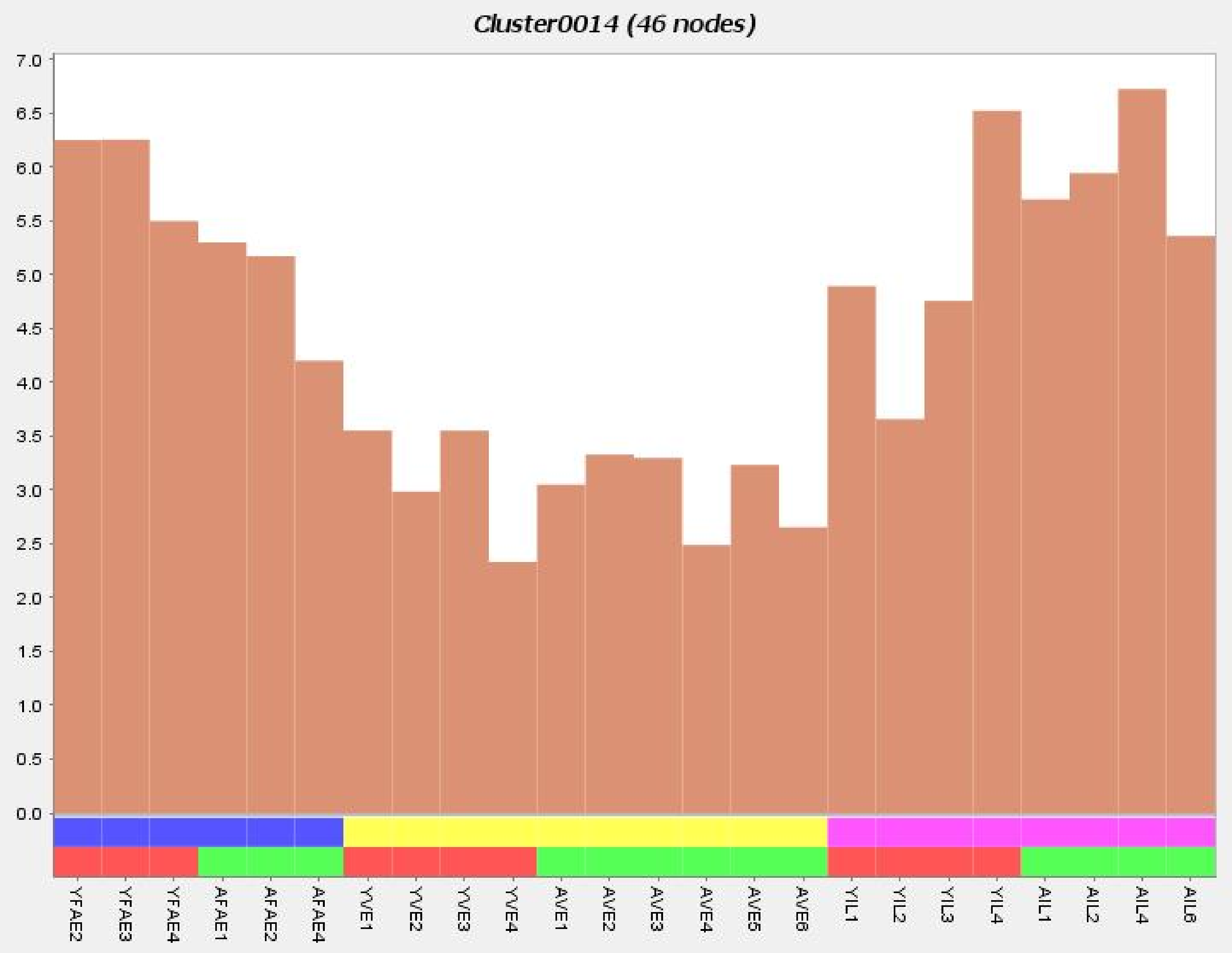
Cluster0014 (46 nodes)
7.0
6.5
6.0
5.5
5.0
4.5
4.0
3.5
3.0
2.5
2.0
1.5
1.0
0.5
0.0
YFAE2
YFAE3
YFAE4
AFAE1
AFAE2
AFAE4
YVE1
YVE2
YVE3
YVE4
AVE1
AVE2
AVE3
AVE4
AVE5
AVE6
YIL1
YIL2
YIL3
YIL4
AIL1
AIL2
AIL4
AIL6

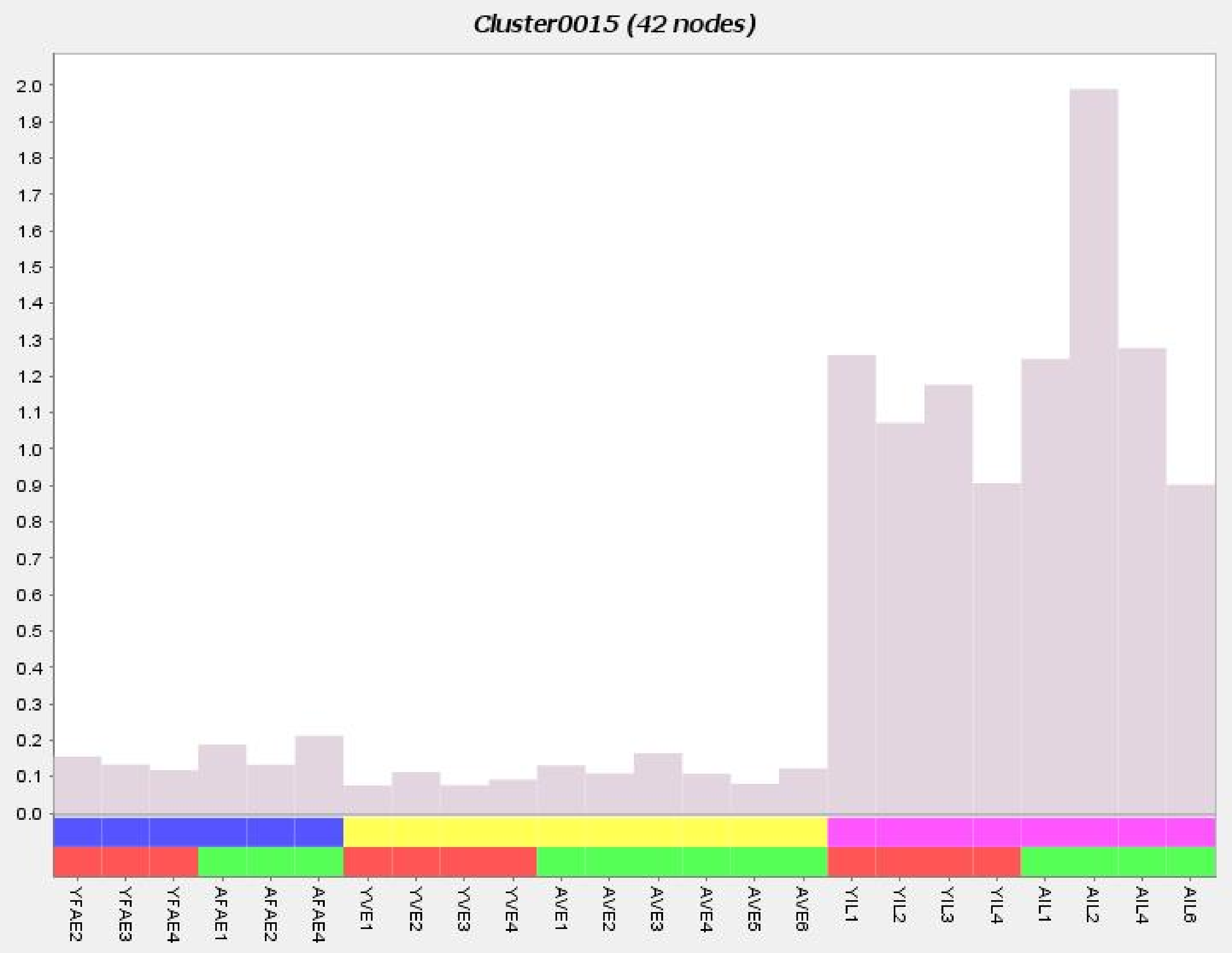

Cluster0015 (42 nodes)
2.0
1.9
1.8
1.7
1.6
1.5
1.4
1.3
1.2
1.1
1.0
0.9
0.8
0.7
0.6
0.5
0.4
0.3
0.2
0.1
0.0
YFAE2
YFAE3
YFAE4
AFAE1
AFAE2
AFAE4
YVE1
YVE2
YVE3
YVE4
AVE1
AVE2
AVE3
AVE4
AVE5
AVE6
YIL1
YIL2
YIL3
YIL4
AIL1
AIL2
AIL4
AIL6

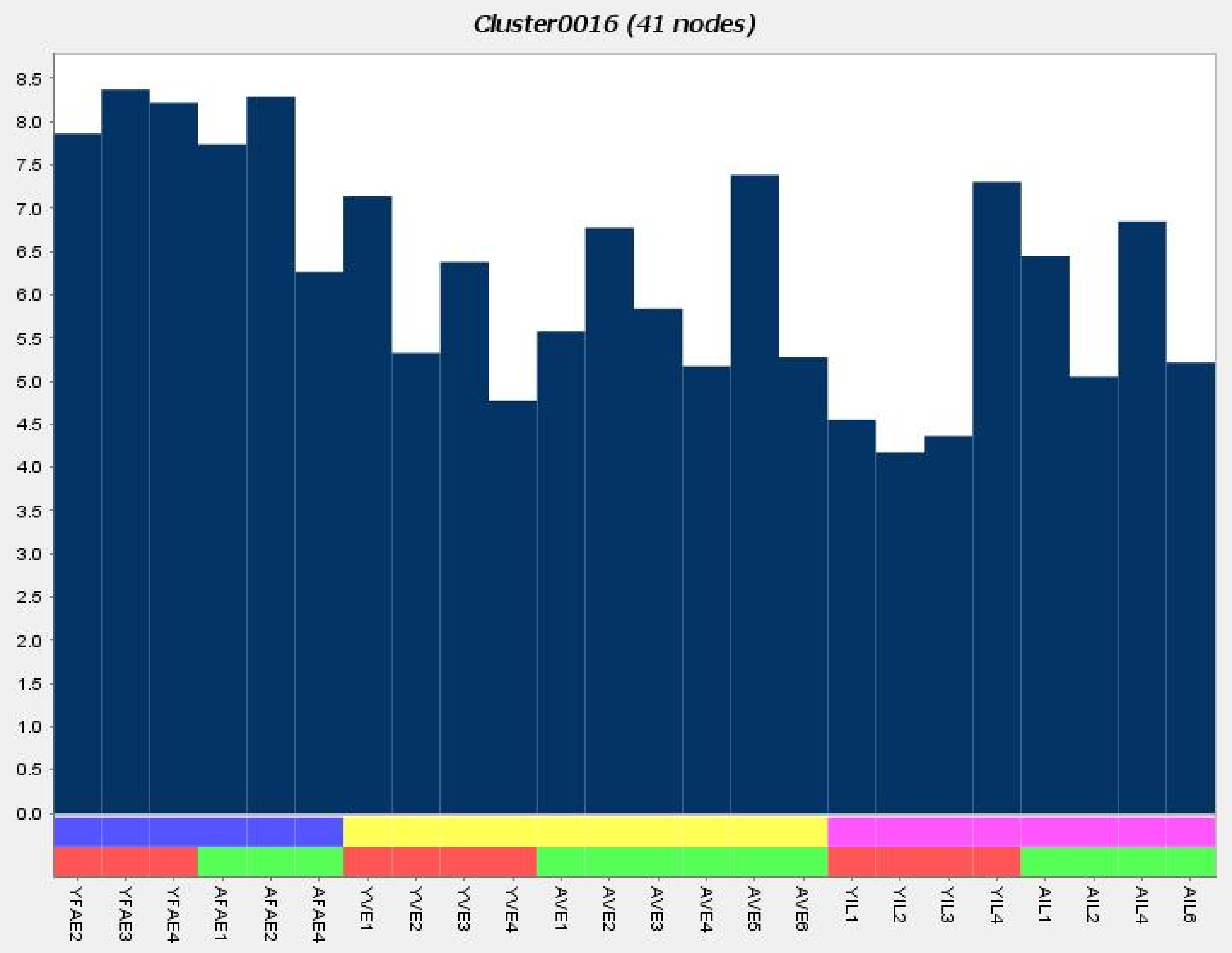

Cluster0016 (41 nodes)
8.5
8.0
7.5
7.0
6.5
6.0
5.5
5.0
4.5
4.0
3.5
3.0
2.5
2.0
1.5
1.0
0.5
0.0
YFAE2
YFAE3
YFAE4
AFAE1
AFAE2
AFAE4
YVE1
YVE2
YVE3
YVE4
AVE1
AVE2
AVE3
AVE4
AVE5
AVE6
YIL1
YIL2
YIL3
YIL4
AIL1
AIL2
AIL4
AIL6

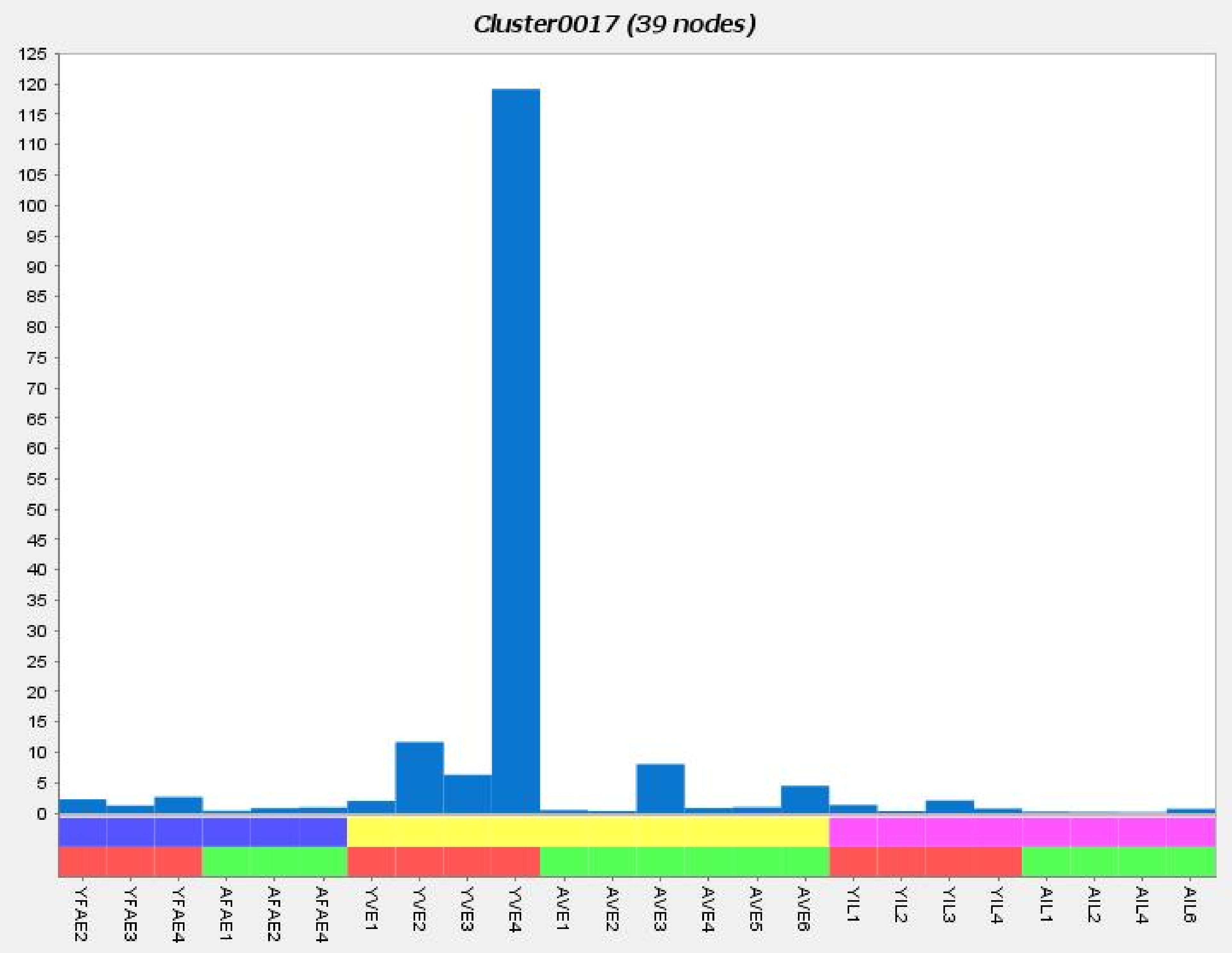
Cluster0017 (39 nodes)
125
120
115
110
105
100
95
90
85
80
75
70
65
60
55
50
45
40
35
30
25
20
15
10
5
0
YFAE2
YFAE3
YFAE4
AFAE1
AFAE2
AFAE4
YVE1
YVE2
YVE3
YVE4
AVE1
AVE2
AVE3
AVE4
AVE5
AVE6
YIL1
YIL2
YIL3
YIL4
AIL1
AIL2
AIL4
AIL6

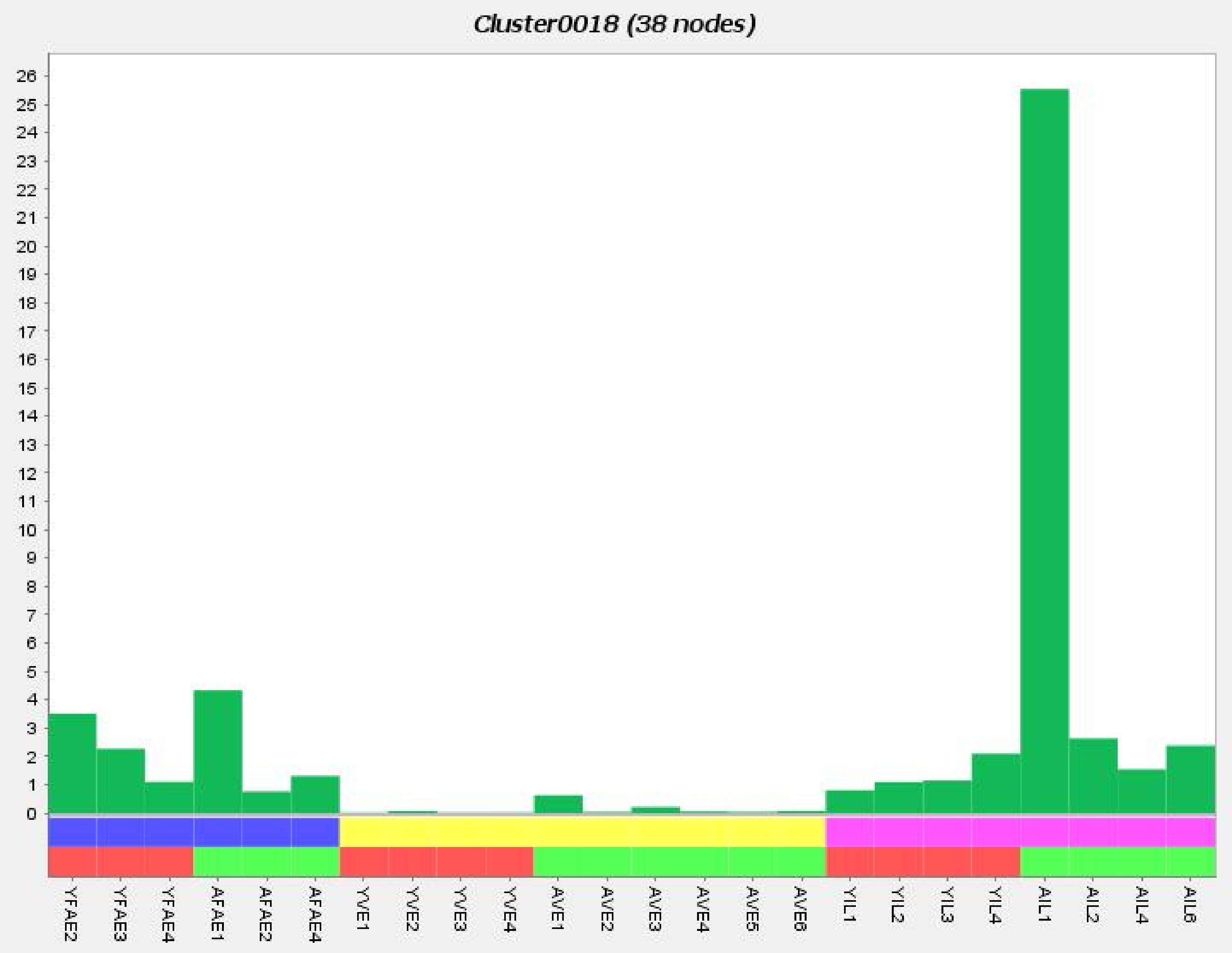

Cluster0018 (38 nodes)
26
25
24
23
22
21
20
19
18
17
16
15
14
13
12
11
10
9
8
7
6
5
4
3
2
1
0
YFAE2
YFAE3
YFAE4
AFAE1
AFAE2
AFAE4
YVE1
YVE2
YVE3
YVE4
AVE1
AVE2
AVE3
AVE4
AVE5
AVE6
YIL1
YIL2
YIL3
YIL4
AIL1
AIL2
AIL4
AIL6

Cluster0019 (38 nodes)

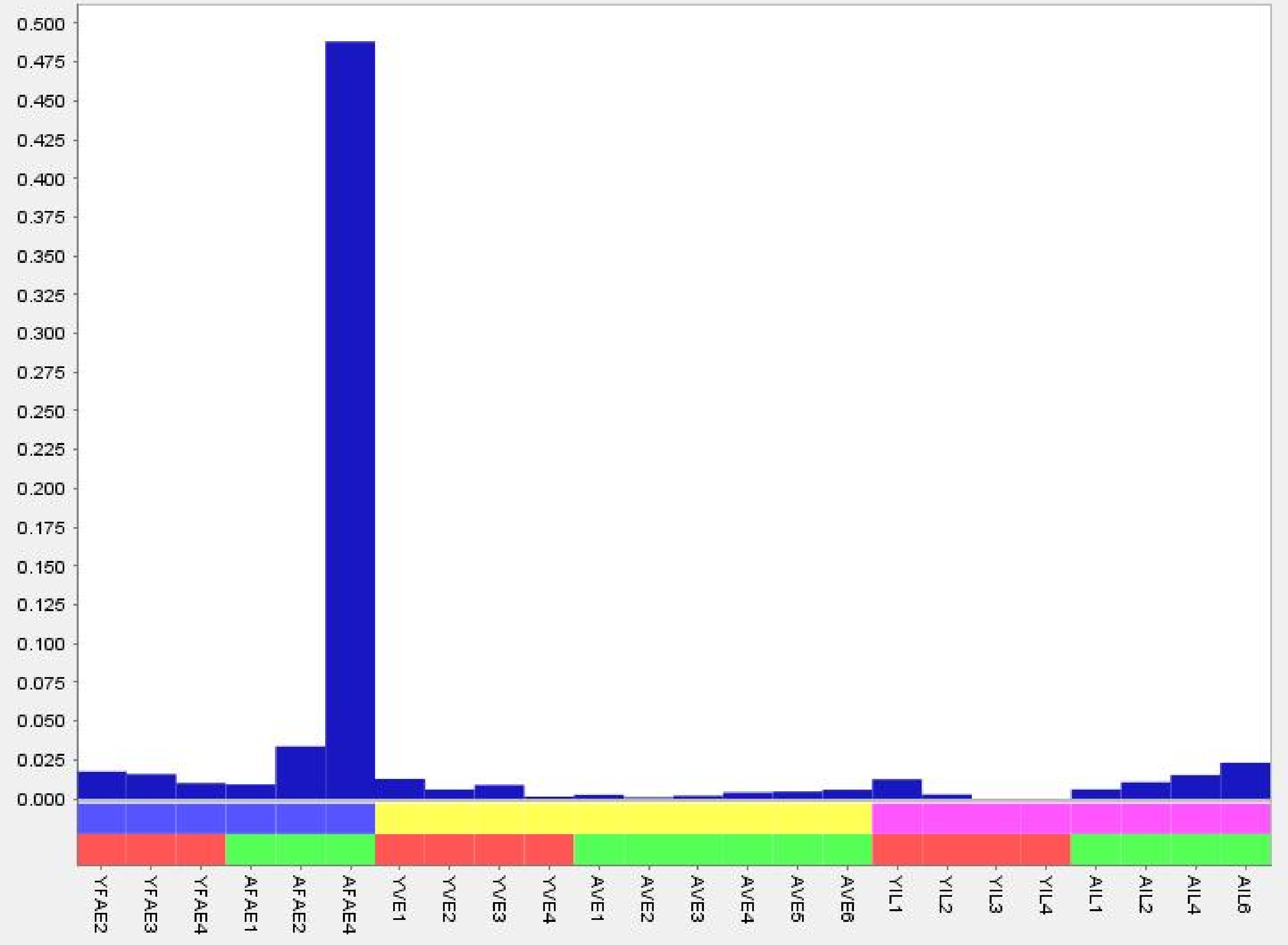

Cluster0020 (37 nodes)

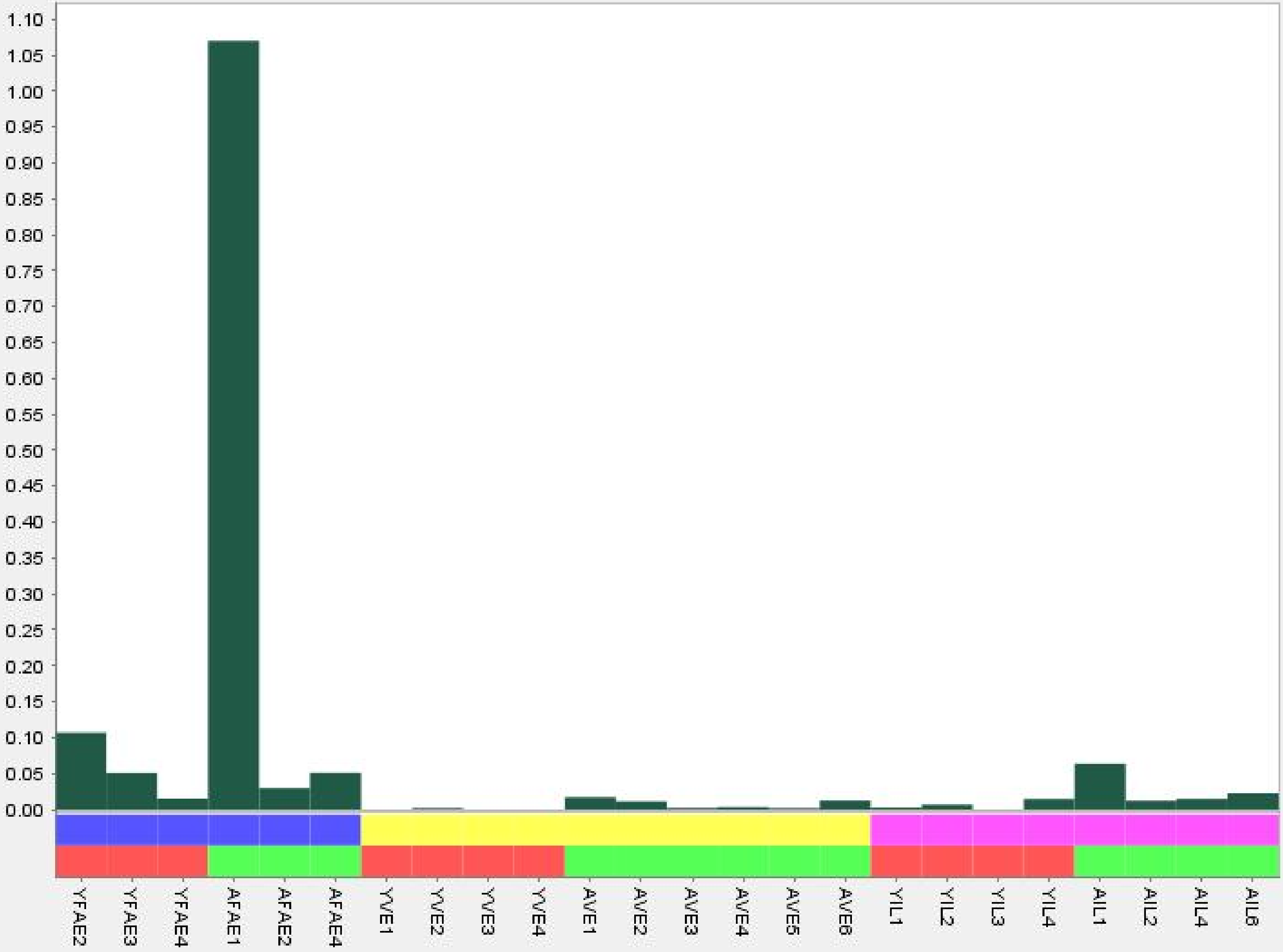

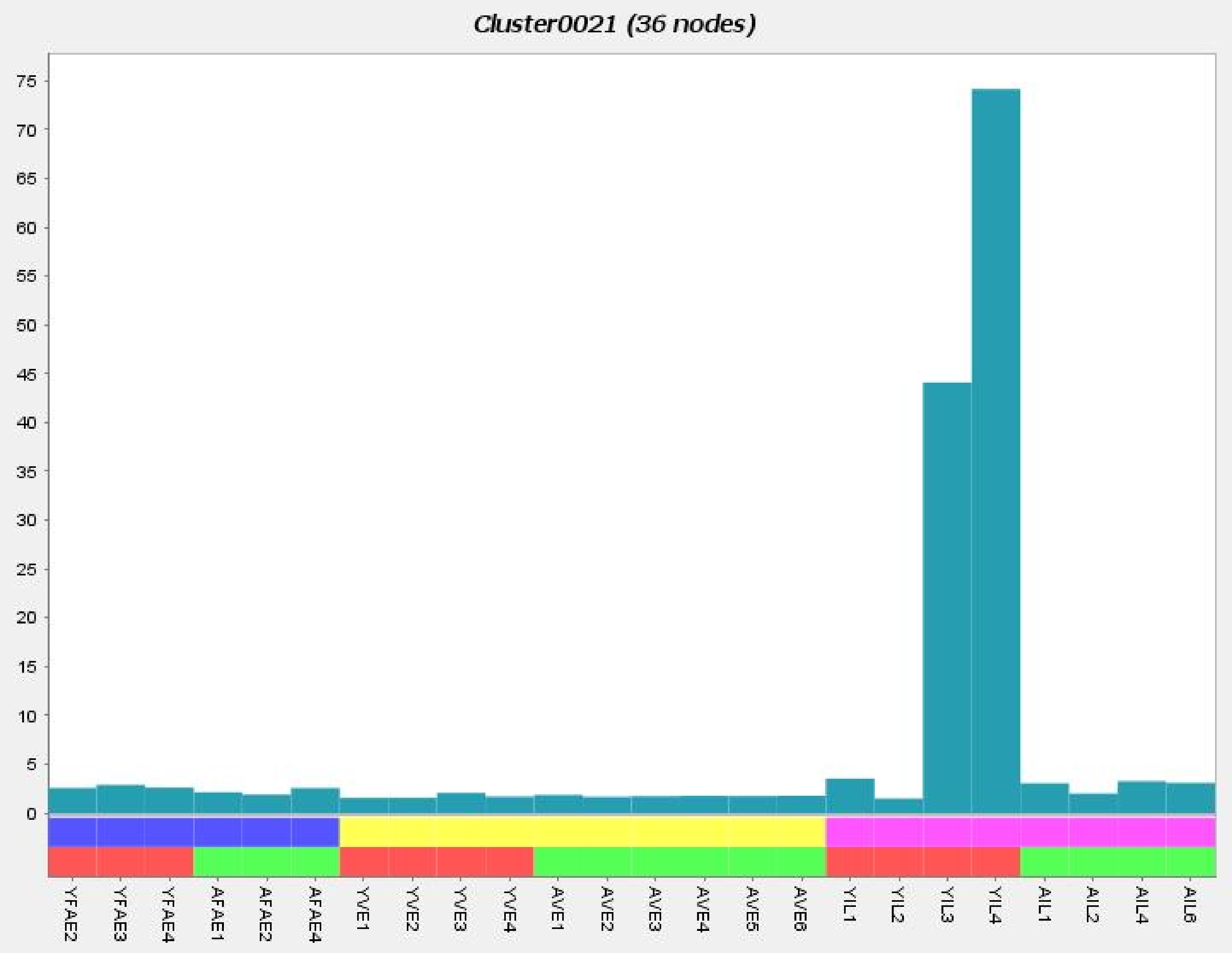

Cluster0021 (36 nodes)
75
70
65
60
55
50
45
40
35
30
25
20
15
10
5
0
YFAE2
YFAE3
YFAE4
AFAE1
AFAE2
AFAE4
YVE1
YVE2
YVE3
YVE4
AVE1
AVE2
AVE3
AVE4
AVE5
AVE6
YIL1
YIL2
YIL3
YIL4
AIL1
AIL2
AIL4
AIL6

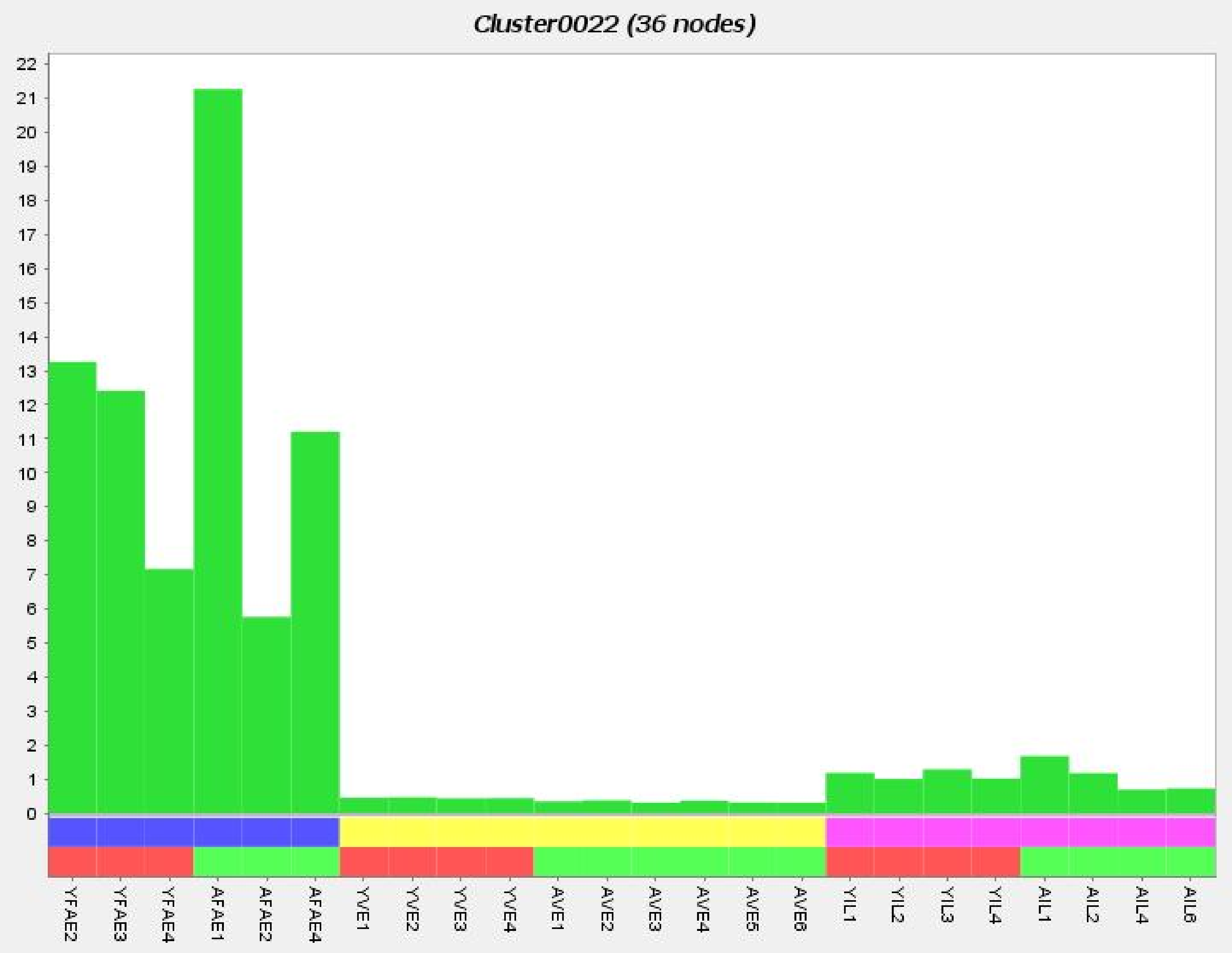

Cluster0022 (36 nodes)
22
21
20
19
18
17
16
15
14
13
12
11
10
9
8
7
6
5
4
3
2
1
0
YFAE2
YFAE3
YFAE4
AFAE1
AFAE2
AFAE4
YVE1
YVE2
YVE3
YVE4
AVE1
AVE2
AVE3
AVE4
AVE5
AVE6
YIL1
YIL2
YIL3
YIL4
AIL1
AIL2
AIL4
AIL6

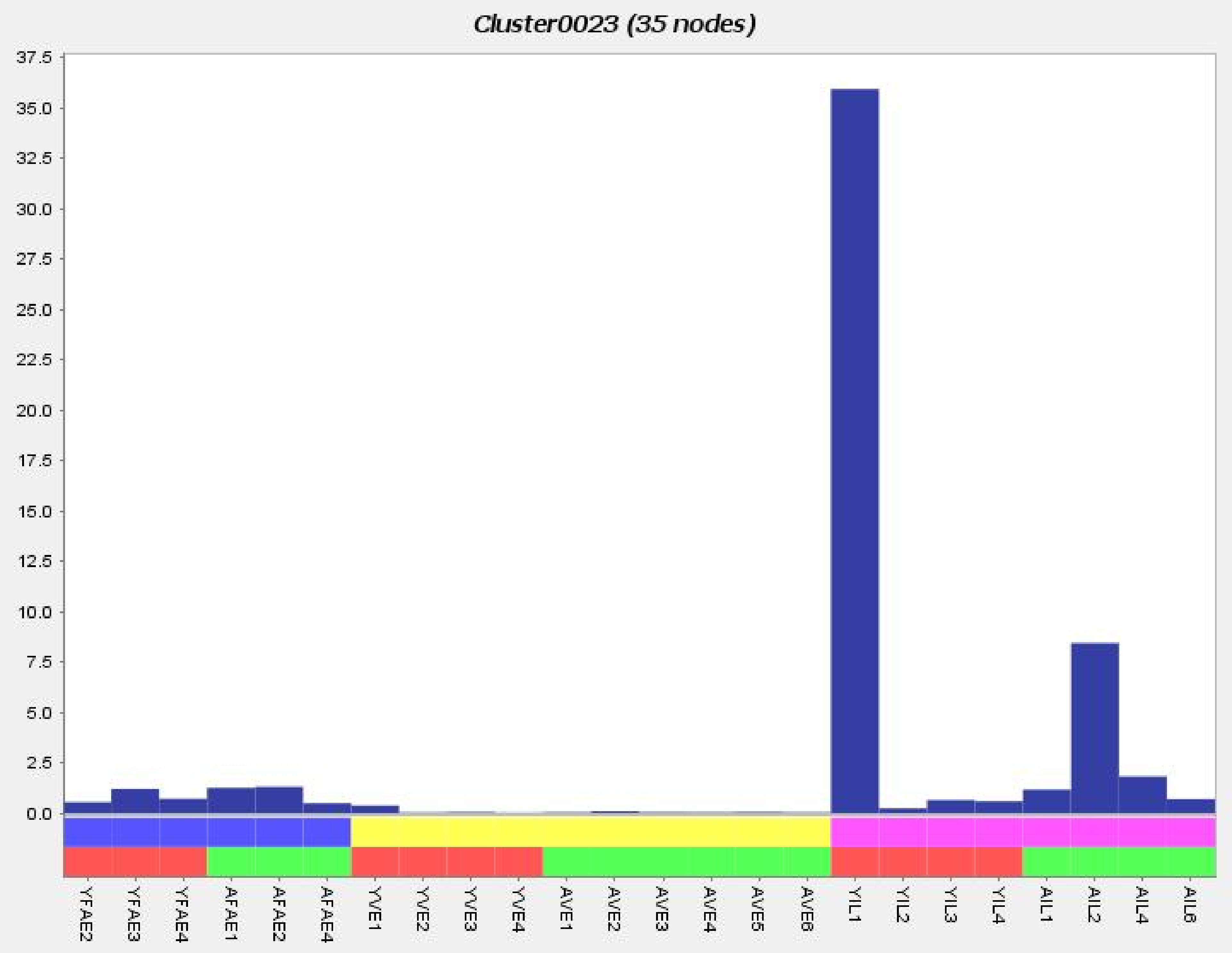
Cluster0023 (35 nodes)
37.5
35.0
32.5
30.0
27.5
25.0
22.5
20.0
17.5
15.0
12.5
10.0
7.5
5.0
2.5
0.0
YFAE2
YFAE3
YFAE4
AFAE1
AFAE2
AFAE4
YVE1
YVE2
YVE3
YVE4
AVE1
AVE2
AVE3
AVE4
AVE5
AVE6
YIL1
YIL2
YIL3
YIL4
AIL1
AIL2
AIL4
AIL6

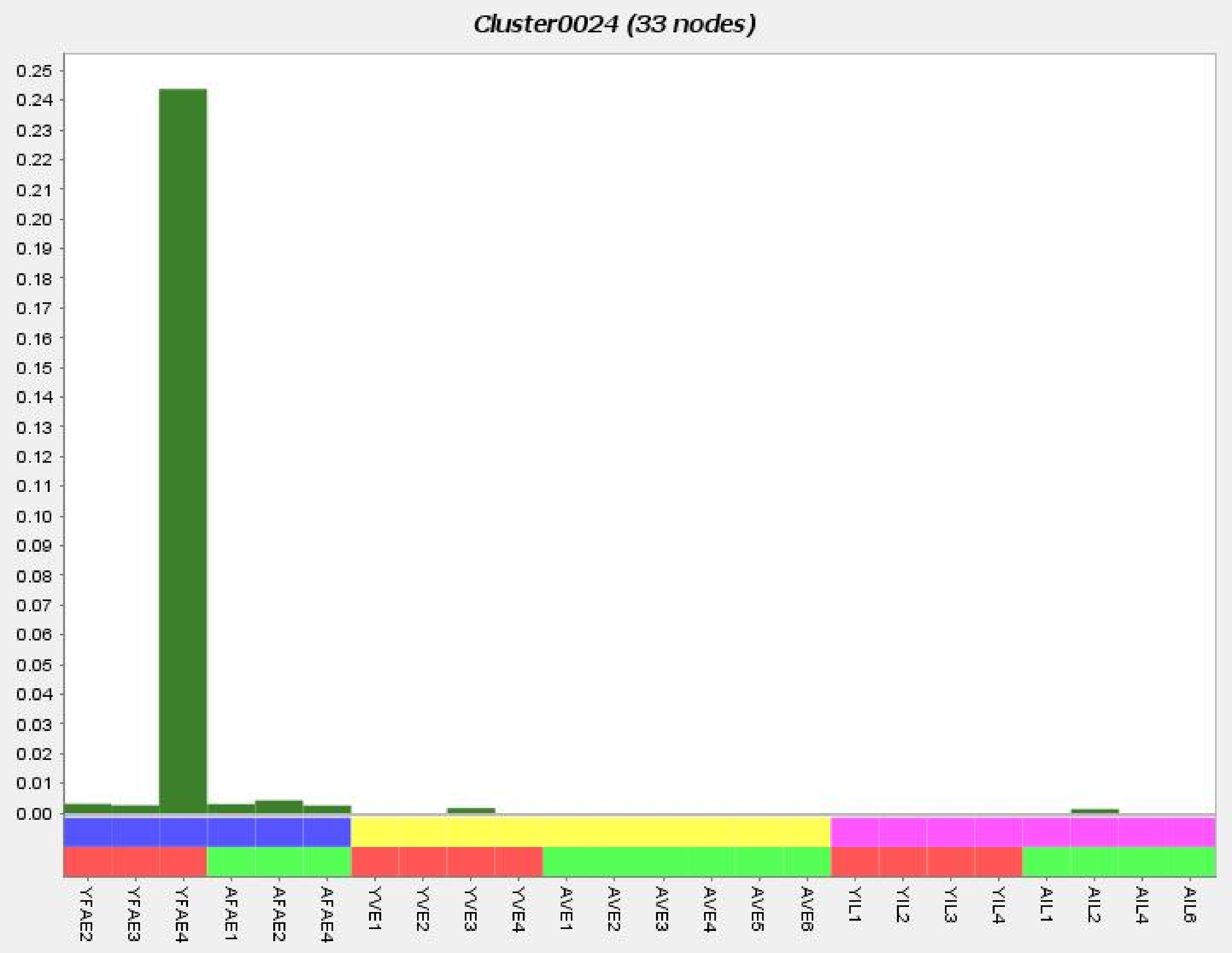
Cluster0024 (33 nodes)
0.25
0.24
0.23
0.22
0.21
0.20
0.19
0.18
0.17
0.16
0.15
0.14
0.13
0.12
0.11
0.10
0.09
0.08
0.07
0.06
0.05
0.04
0.03
0.02
0.01
0.00
YFAE2
YFAE3
YFAE4
AFAE1
AFAE2
AFAE4
YVE1
YVE2
YVE3
YVE4
AVE1
AVE2
AVE3
AVE4
AVE5
AVE6
YIL1
YIL2
YIL3
YIL4
AIL1
AIL2
AIL4
AIL6

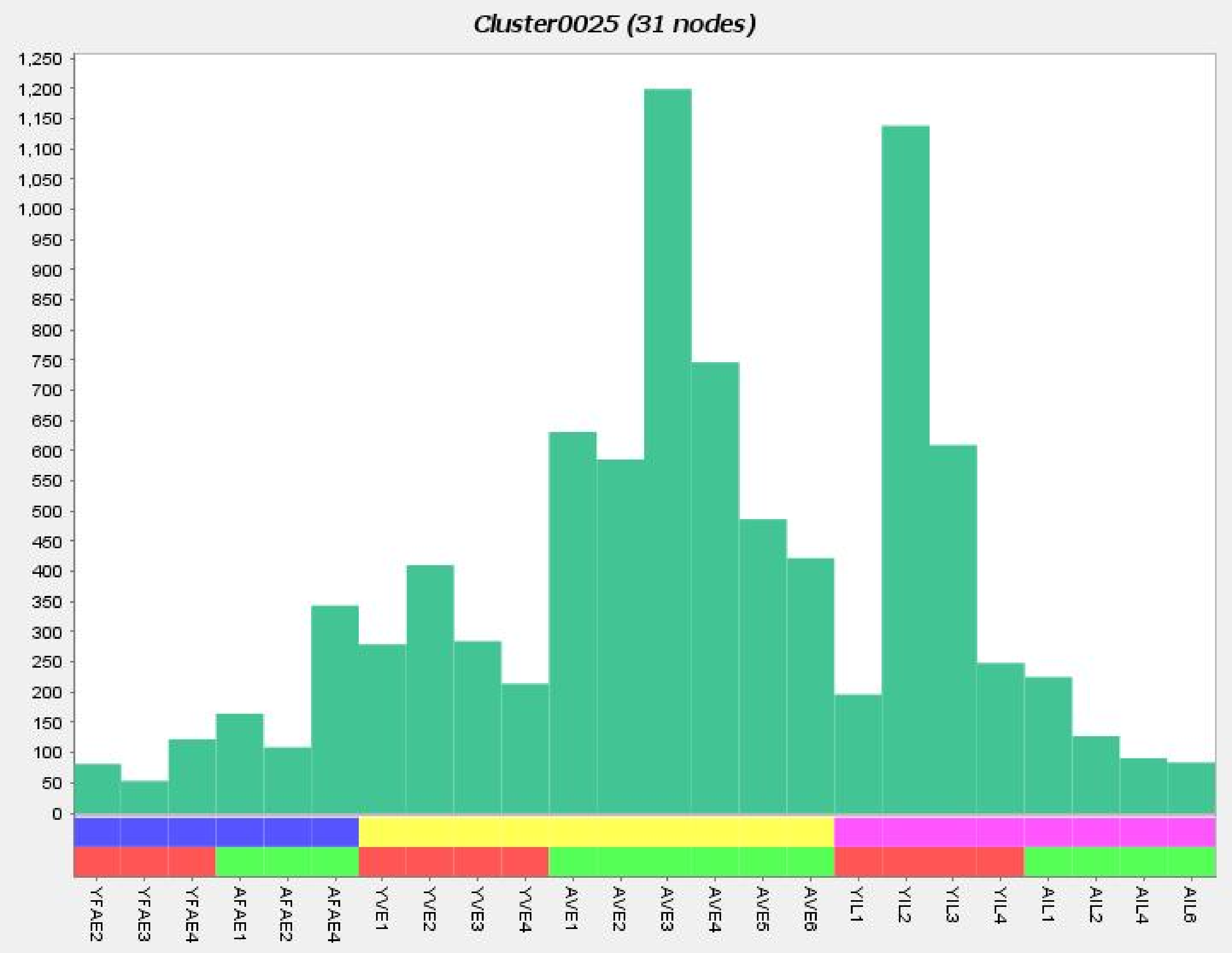
Cluster0025 (31 nodes)
1,250
1,200
1,150
1,100
1,050
1,000
950
900
850
800
750
700
650
600
550
500
450
400
350
300
250
200
150
100
50
0
YFAE2
YFAE3
YFAE4
AFAE1
AFAE2
AFAE4
YVE1
YVE2
YVE3
YVE4
AVE1
AVE2
AVE3
AVE4
AVE5
AVE6
YIL1
YIL2
YIL3
YIL4
AIL1
AIL2
AIL4
AIL6

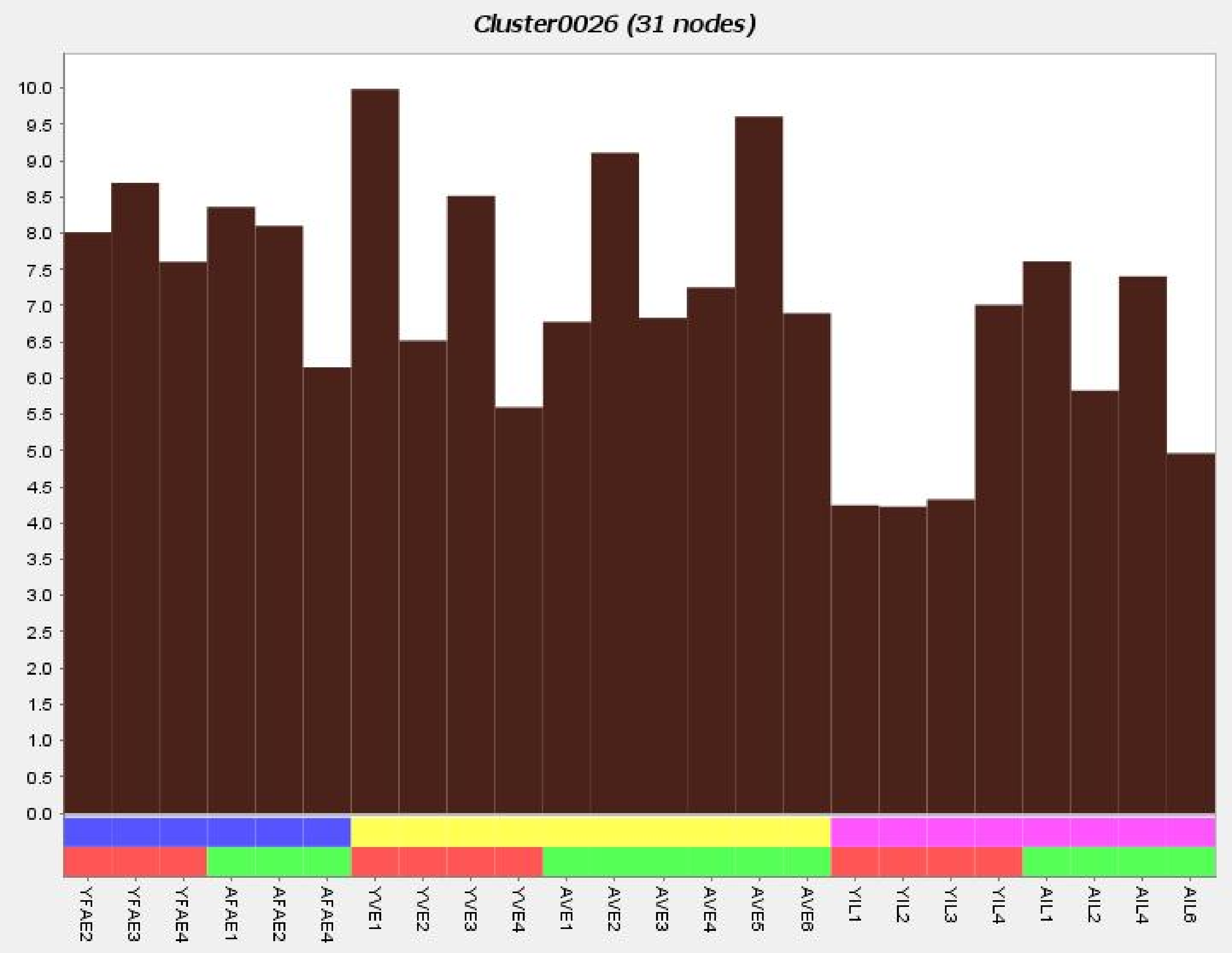
Cluster0026 (31 nodes)
10.0
9.5
9.0
8.5
8.0
7.5
7.0
6.5
6.0
5.5
5.0
4.5
4.0
3.5
3.0
2.5
2.0
1.5
1.0
0.5
0.0
YFAE2
YFAE3
YFAE4
AFAE1
AFAE2
AFAE4
YVE1
YVE2
YVE3
YVE4
AVE1
AVE2
AVE3
AVE4
AVE5
AVE6
YIL1
YIL2
YIL3
YIL4
AIL1
AIL2
AIL4
AIL6

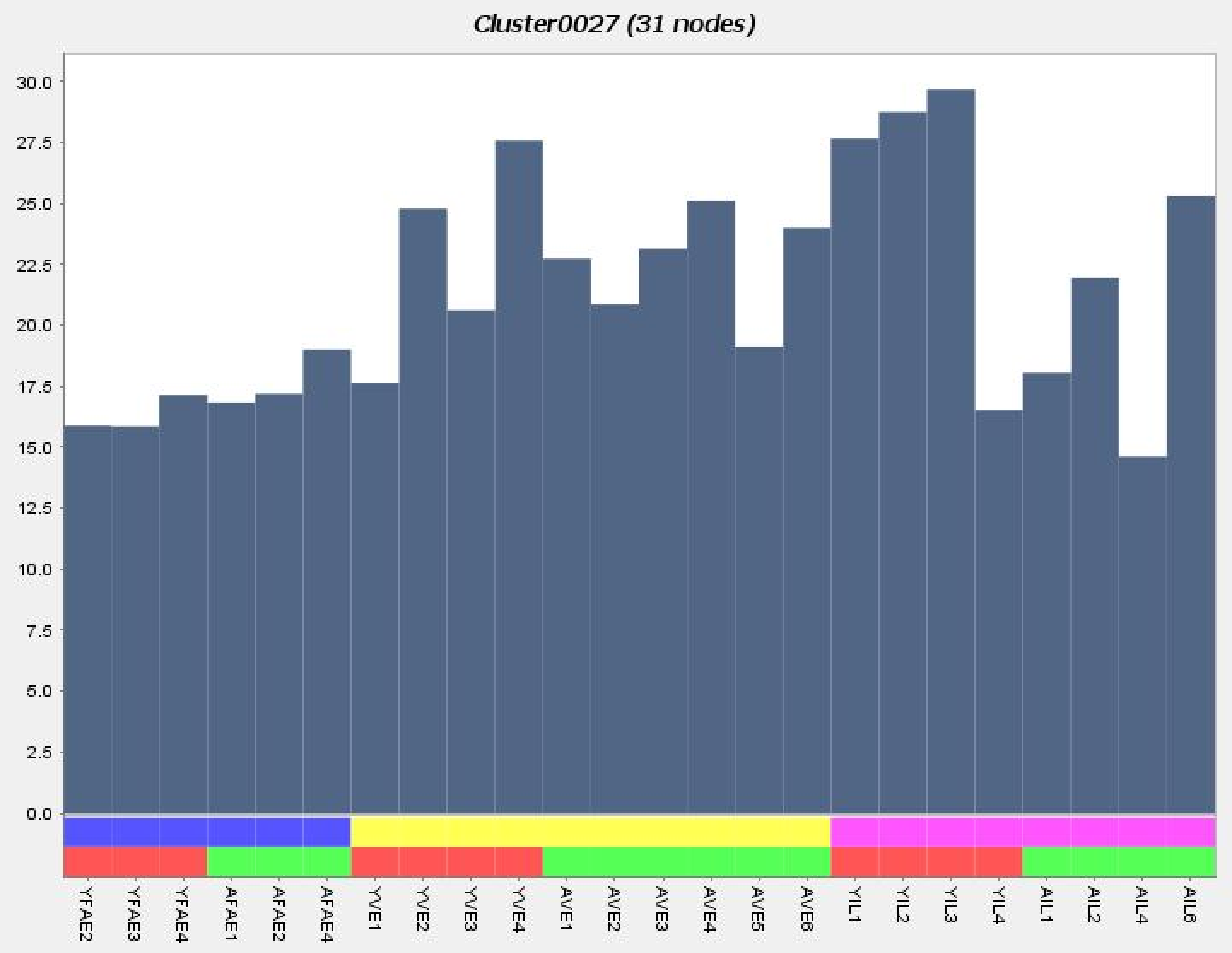

Cluster0027 (31 nodes)
30.0
27.5
25.0
22.5
20.0
17.5
15.0
12.5
10.0
7.5
5.0
2.5
0.0
YFAE2
YFAE3
YFAE4
AFAE1
AFAE2
AFAE4
YVE1
YVE2
YVE3
YVE4
AVE1
AVE2
AVE3
AVE4
AVE5
AVE6
YIL1
YIL2
YIL3
YIL4
AIL1
AIL2
AIL4
AIL6

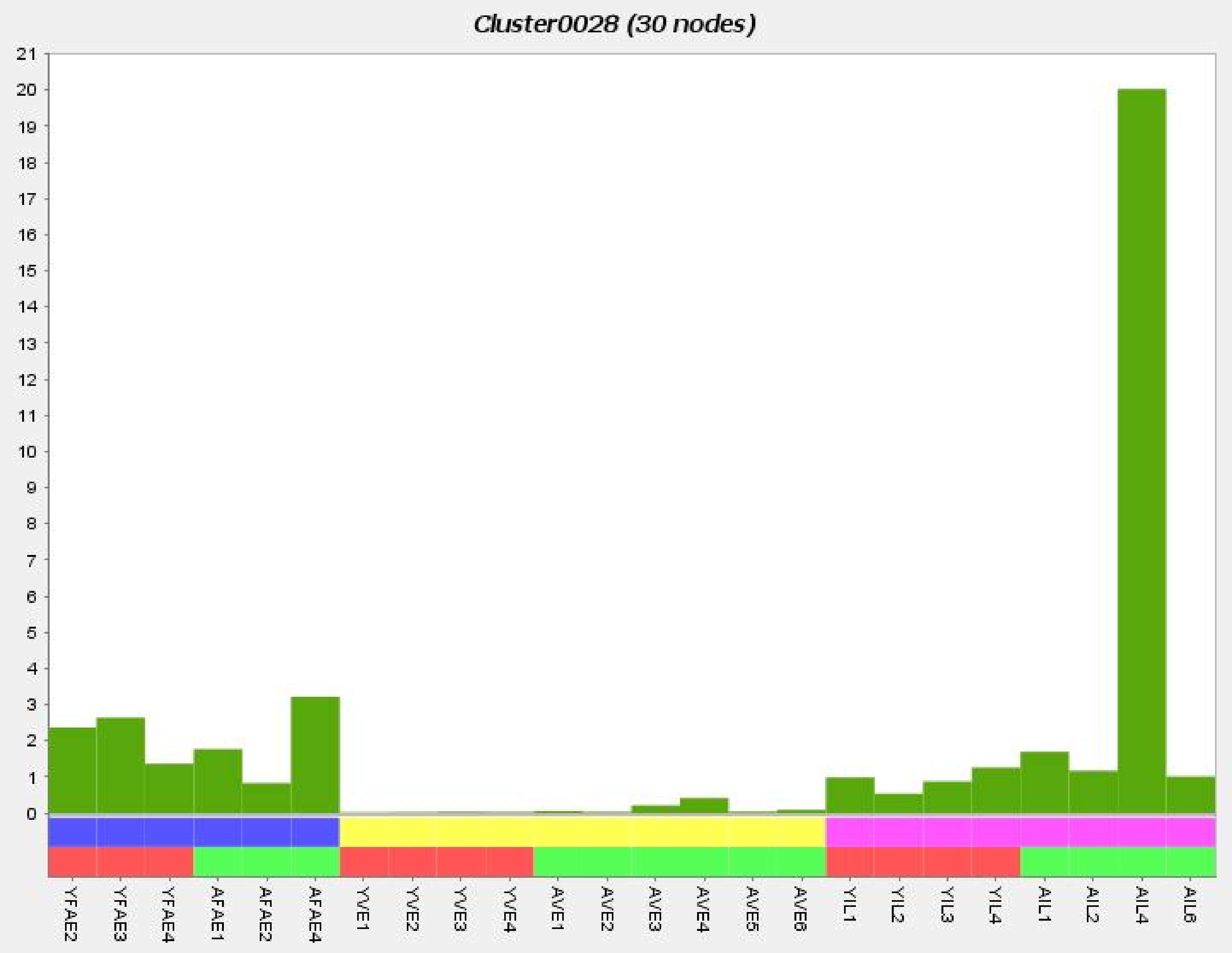

Cluster0028 (30 nodes)
21
20
19
18
17
16
15
14
13
12
11
10
9
8
7
6
5
4
3
2
1
0
YFAE2
YFAE3
YFAE4
AFAE1
AFAE2
AFAE4
YVE1
YVE2
YVE3
YVE4
AVE1
AVE2
AVE3
AVE4
AVE5
AVE6
YIL1
YIL2
YIL3
YIL4
AIL1
AIL2
AIL4
AIL6

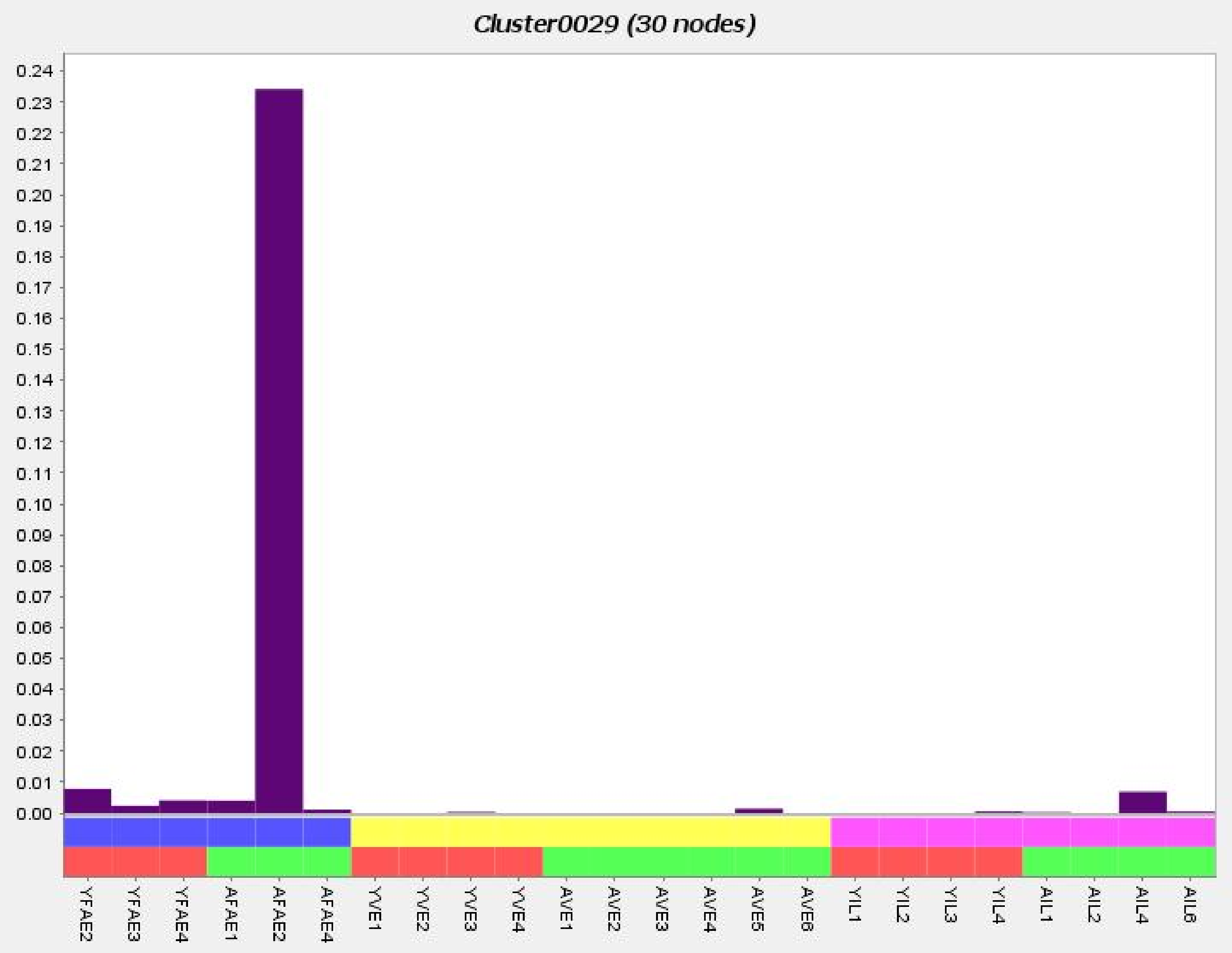

Cluster0029 (30 nodes)
0.24
0.23
0.22
0.21
0.20
0.19
0.18
0.17
0.16
0.15
0.14
0.13
0.12
0.11
0.10
0.09
0.08
0.07
0.06
0.05
0.04
0.03
0.02
0.01
0.00
YFAE2
YFAE3
YFAE4
AFAE1
AFAE2
AFAE4
YVE1
YVE2
YVE3
YVE4
AVE1
AVE2
AVE3
AVE4
AVE5
AVE6
YIL1
YIL2
YIL3
YIL4
AIL1
AIL2
AIL4
AIL6

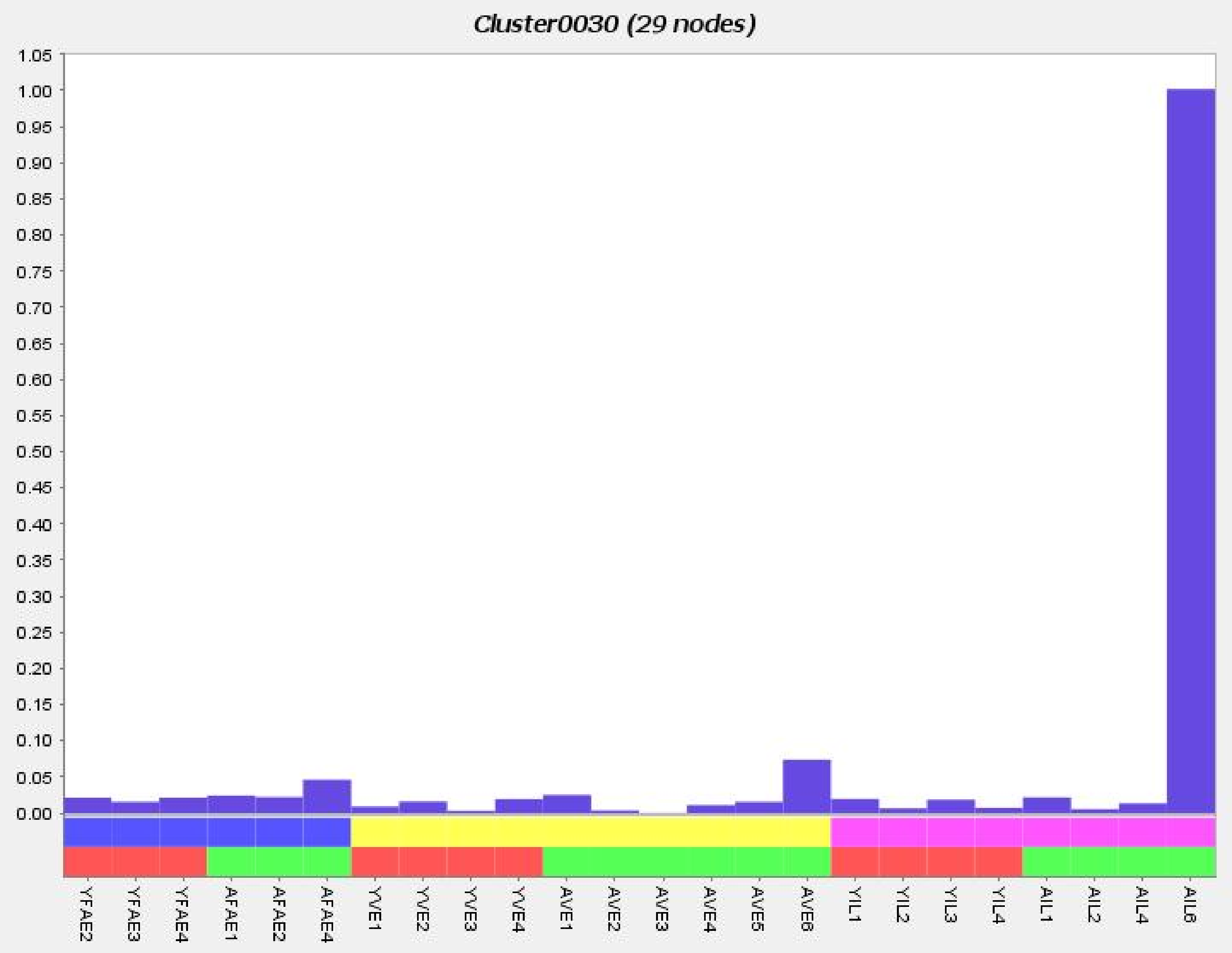

Cluster0030 (29 nodes)
1.05
1.00
0.95
0.90
0.85
0.80
0.75
0.70
0.65
0.60
0.55
0.50
0.45
0.40
0.35
0.30
0.25
0.20
0.15
0.10
0.05
0.00
YFAE2
YFAE3
YFAE4
AFAE1
AFAE2
AFAE4
YVE1
YVE2
YVE3
YVE4
AVE1
AVE2
AVE3
AVE4
AVE5
AVE6
YIL1
YIL2
YIL3
YIL4
AIL1
AIL2
AIL4
AIL6

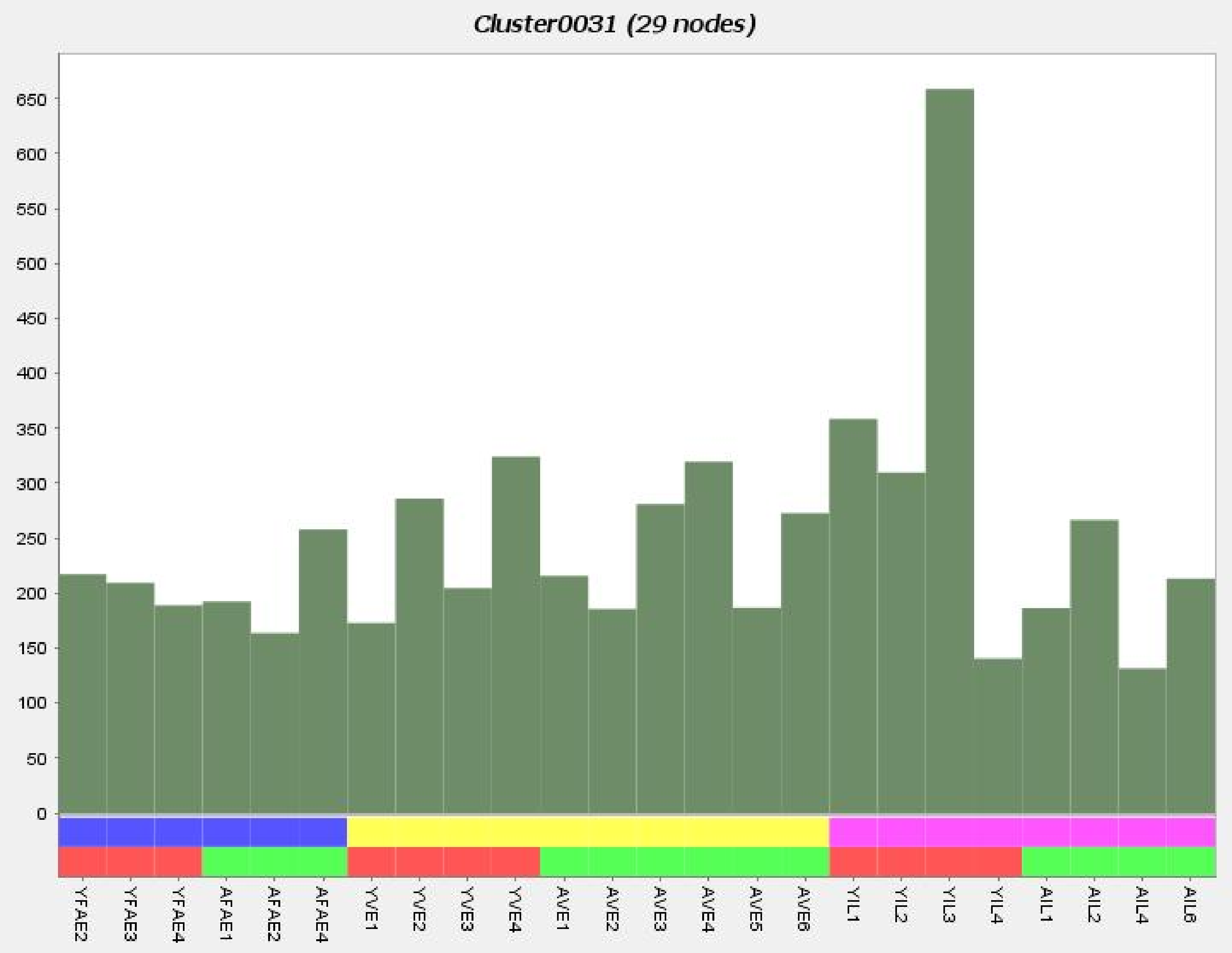
Cluster0031 (29 nodes)
650
600
550
500
450
400
350
300
250
200
150
100
50
0
YFAE2
YFAE3
YFAE4
AFAE1
AFAE2
AFAE4
YVE1
YVE2
YVE3
YVE4
AVE1
AVE2
AVE3
AVE4
AVE5
AVE6
YIL1
YIL2
YIL3
YIL4
AIL1
AIL2
AIL4
AIL6

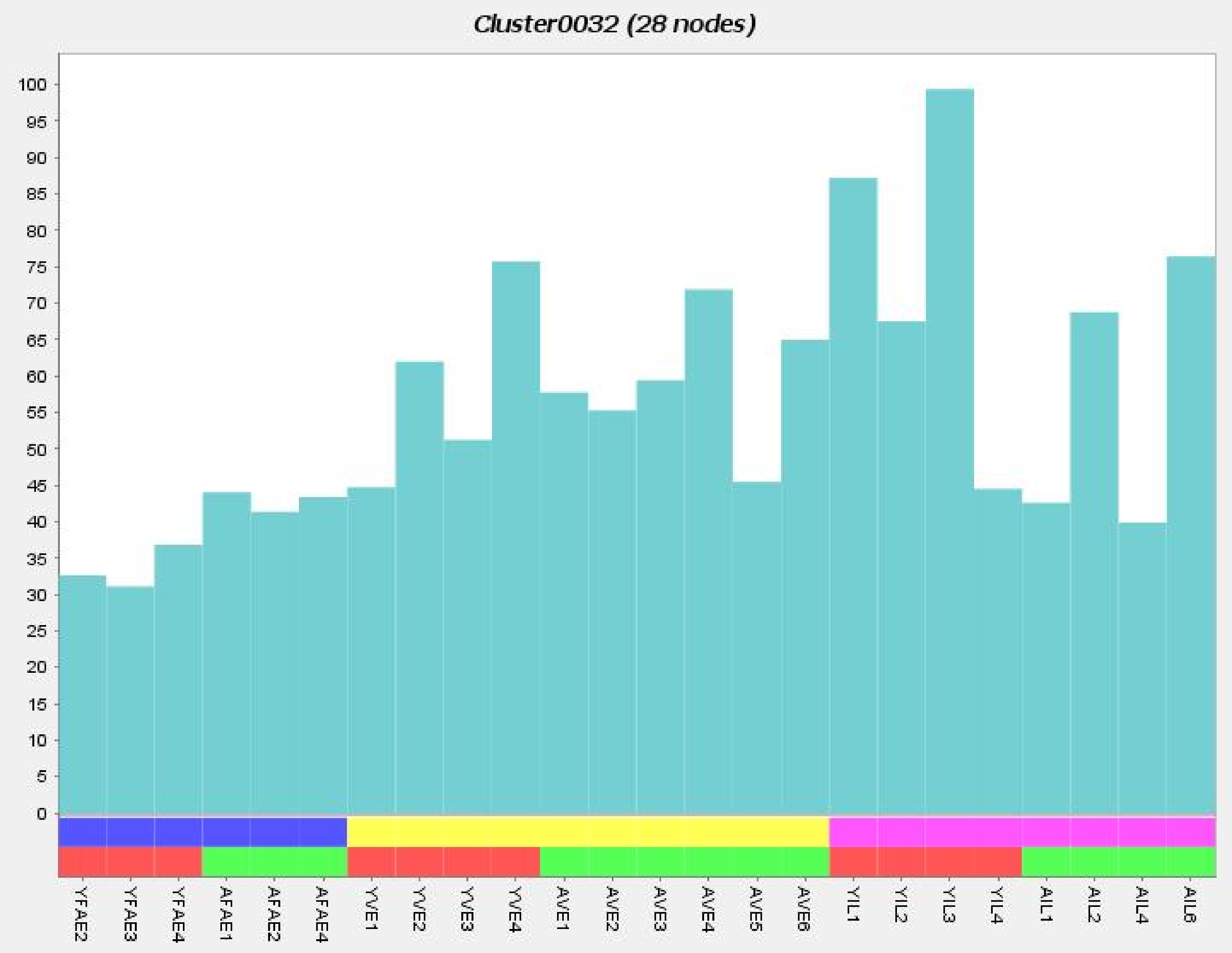

Cluster0032 (28 nodes)
100
95
90
85
80
75
70
65
60
55
50
45
40
35
30
25
20
15
10
5
0
YFAE2
YFAE3
YFAE4
AFAE1
AFAE2
AFAE4
YVE1
YVE2
YVE3
YVE4
AVE1
AVE2
AVE3
AVE4
AVE5
AVE6
YIL1
YIL2
YIL3
YIL4
AIL1
AIL2
AIL4
AIL6

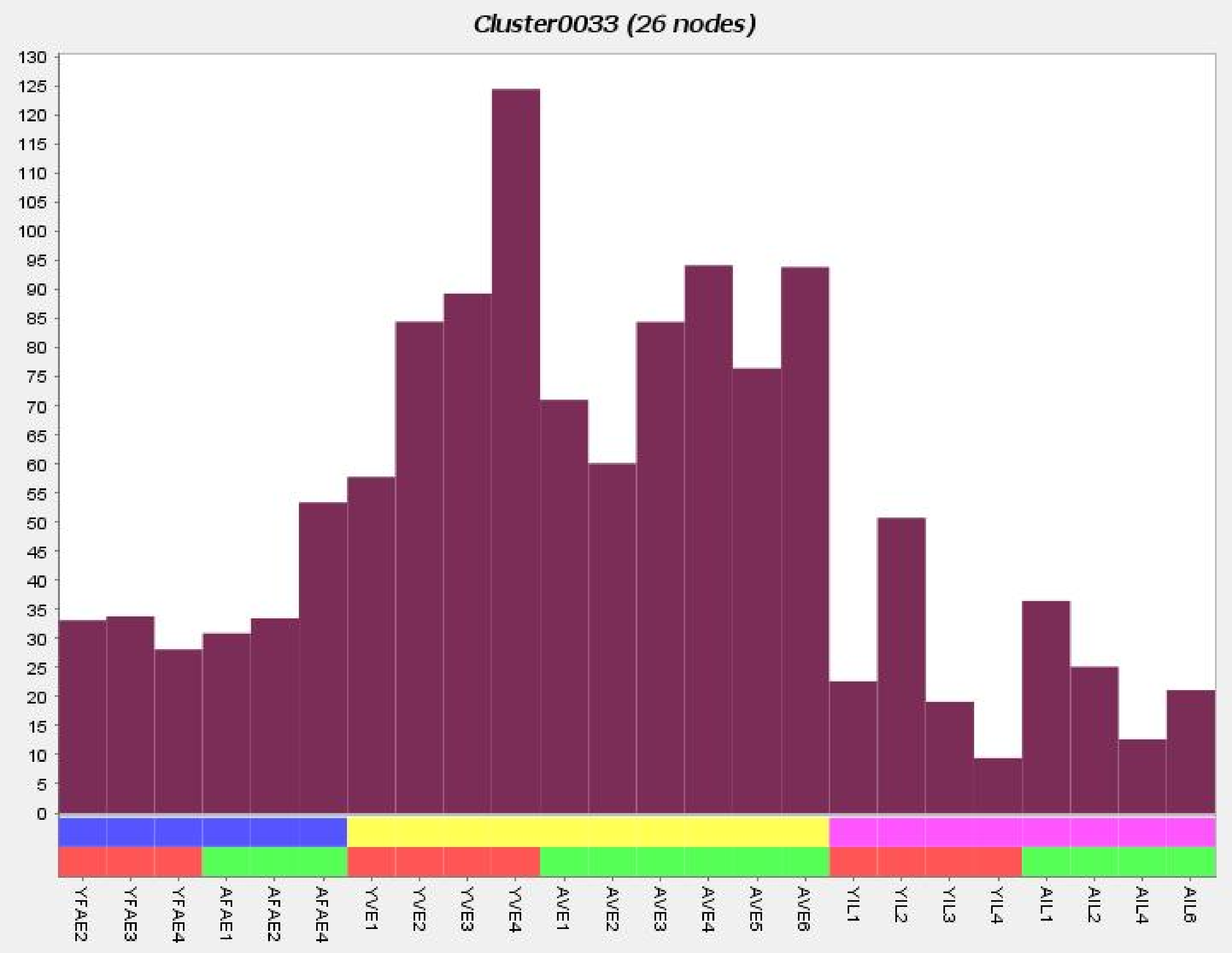
Cluster0033 (26 nodes)
0
5
10
15
20
25
30
35
40
45
50
55
60
65
70
75
80
85
90
95
100
105
110
115
120
125
130
YFAE2
YFAE3
YFAE4
AFAE1
AFAE2
AFAE4
YVE1
YVE2
YVE3
YVE4
AVE1
AVE2
AVE3
AVE4
AVE5
AVE6
YIL1
YIL2
YIL3
YIL4
AIL1
AIL2
AIL4
AIL6

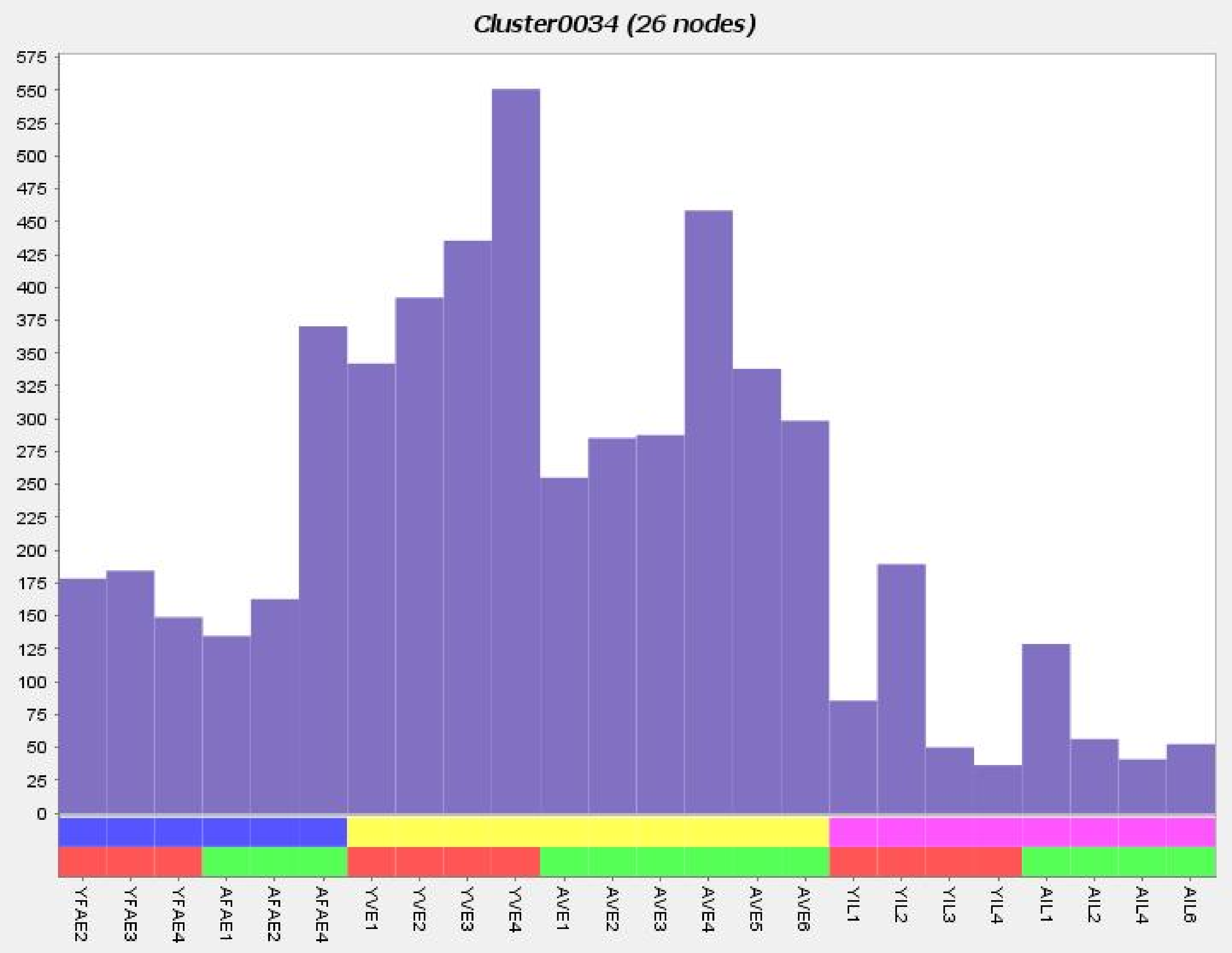

Cluster0034 (26 nodes)
575
550
525
500
475
450
425
400
375
350
325
300
275
250
225
200
175
150
125
100
75
50
25
0
YFAE2
YFAE3
YFAE4
AFAE1
AFAE2
AFAE4
YVE1
YVE2
YVE3
YVE4
AVE1
AVE2
AVE3
AVE4
AVE5
AVE6
YIL1
YIL2
YIL3
YIL4
AIL1
AIL2
AIL4
AIL6

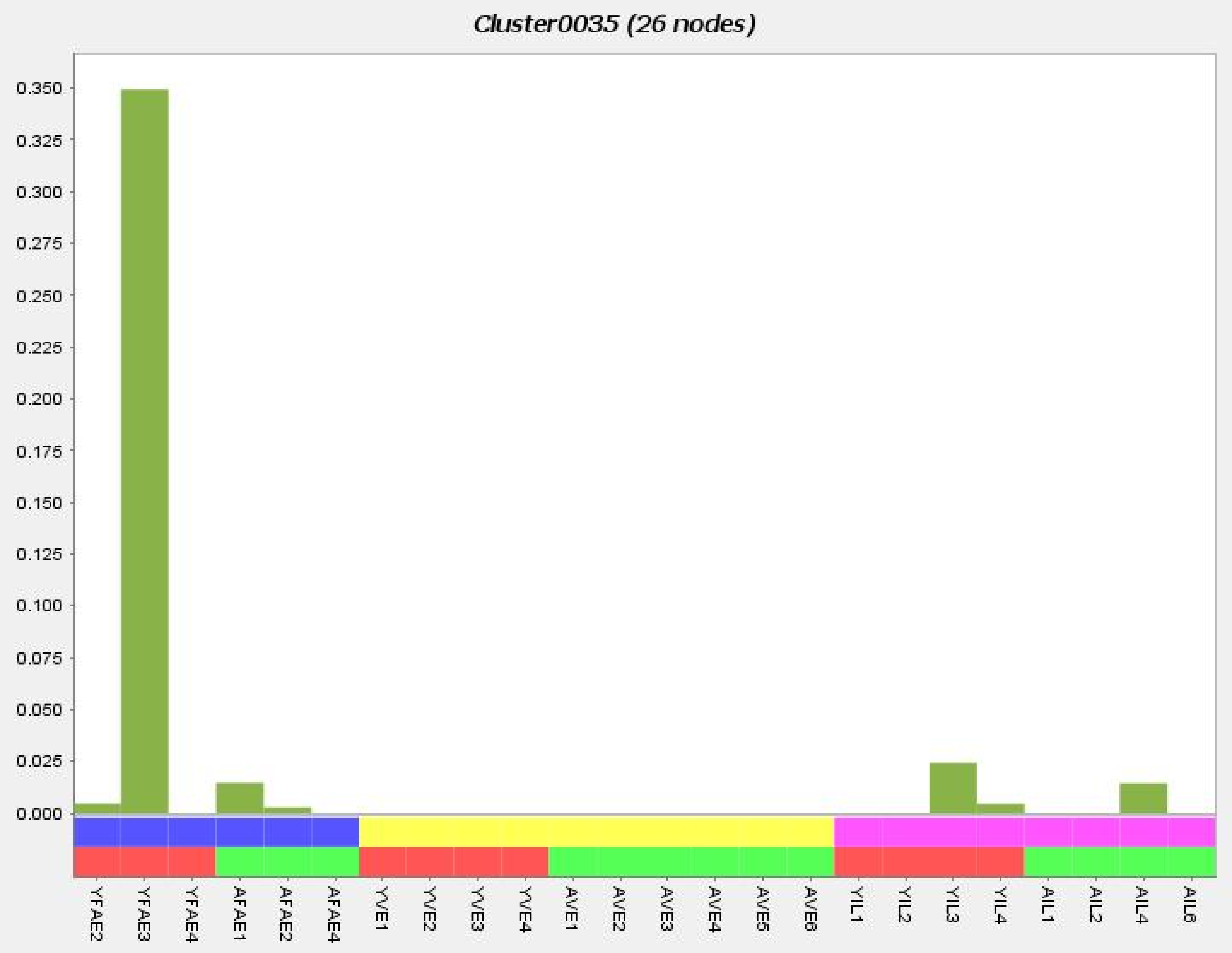
Cluster0035 (26 nodes)
0.350
0.325
0.300
0.275
0.250
0.225
0.200
0.175
0.150
0.125
0.100
0.075
0.050
0.025
0.000
YFAE2
YFAE3
YFAE4
AFAE1
AFAE2
AFAE4
YVE1
YVE2
YVE3
YVE4
AVE1
AVE2
AVE3
AVE4
AVE5
AVE6
YIL1
YIL2
YIL3
YIL4
AIL1
AIL2
AIL4
AIL6

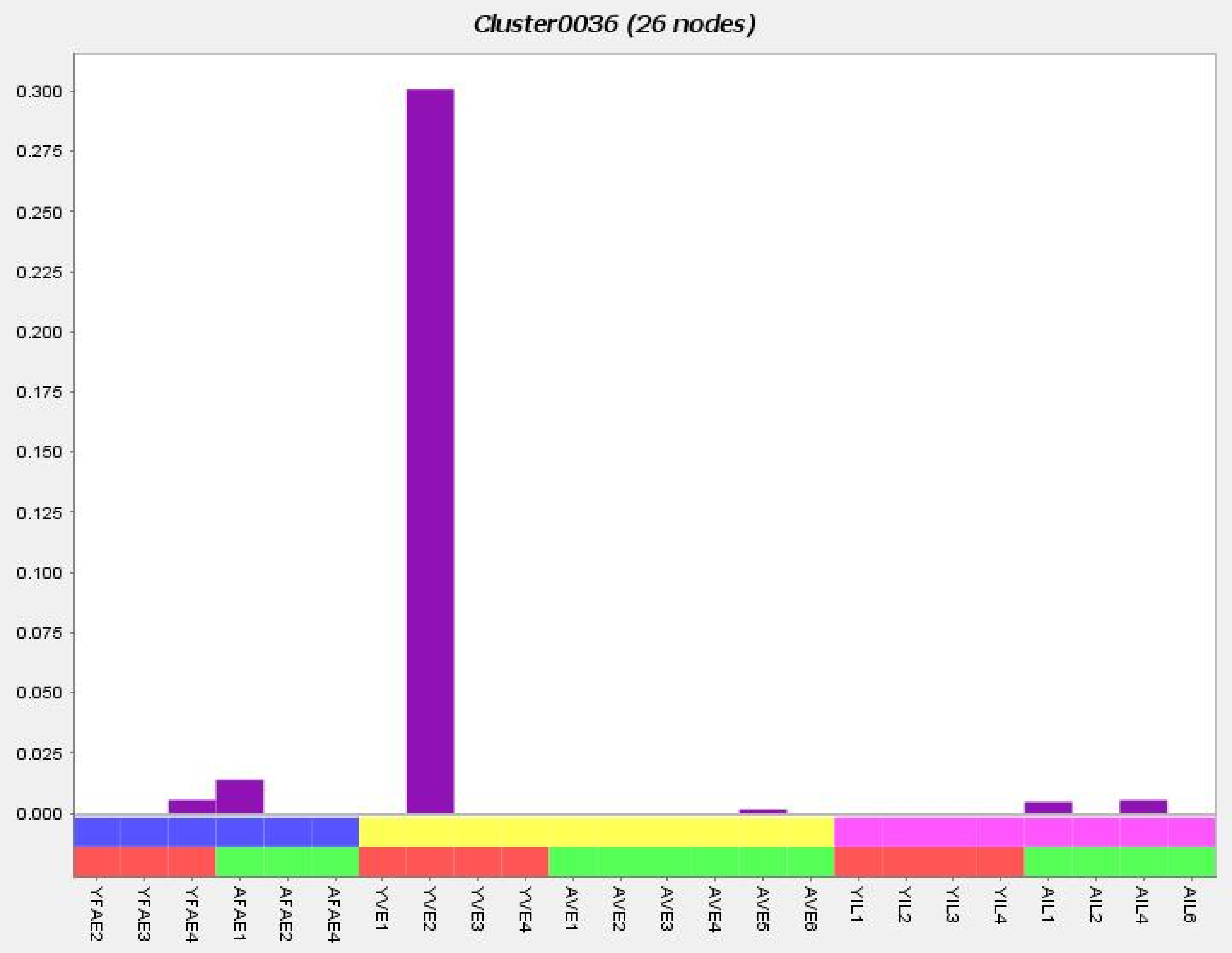
Cluster0036 (26 nodes)
0.300
0.275
0.250
0.225
0.200
0.175
0.150
0.125
0.100
0.075
0.050
0.025
0.000
YFAE2
YFAE3
YFAE4
AFAE1
AFAE2
AFAE4
YVE1
YVE2
YVE3
YVE4
AVE1
AVE2
AVE3
AVE4
AVE5
AVE6
YIL1
YIL2
YIL3
YIL4
AIL1
AIL2
AIL4
AIL6

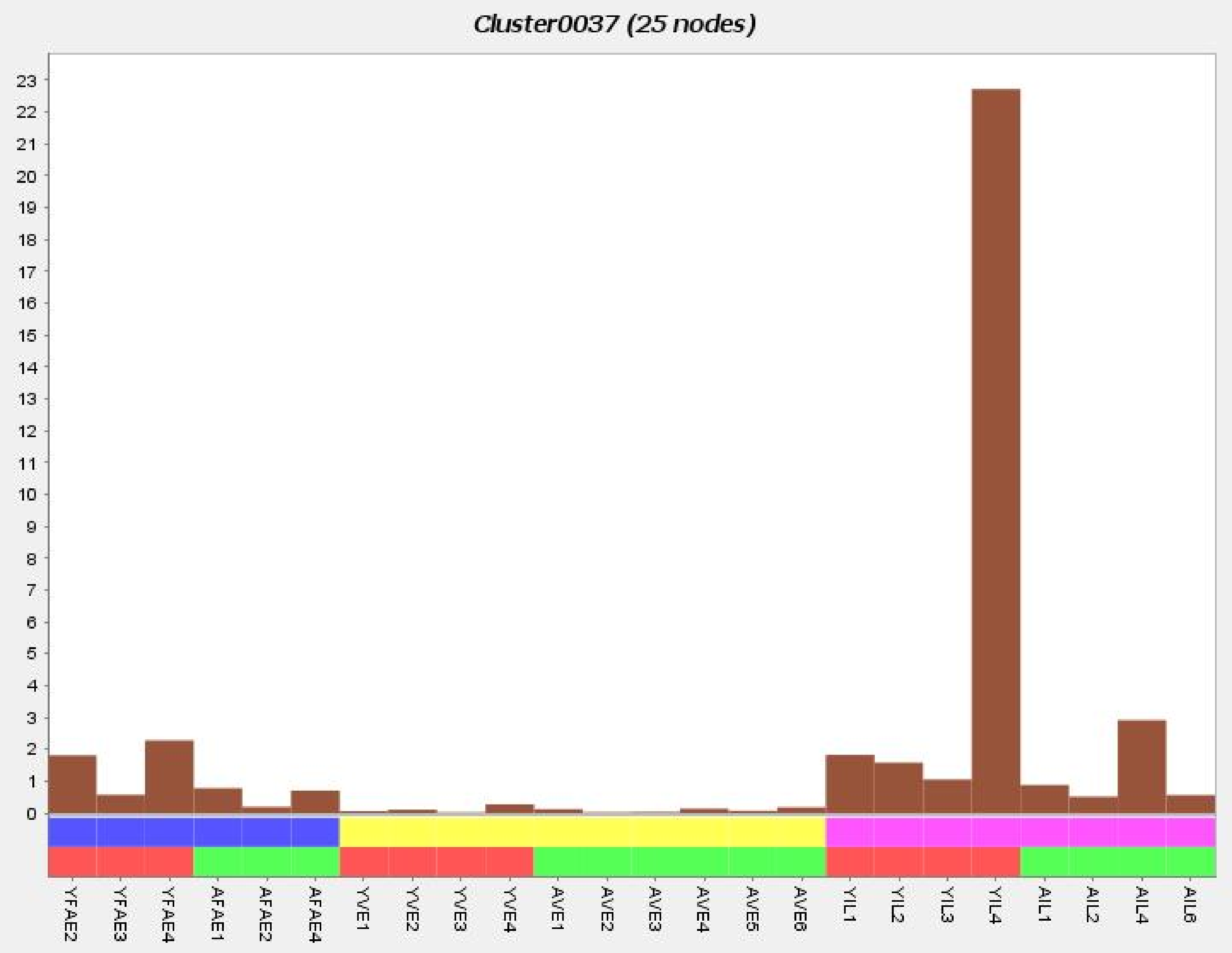
Cluster0037 (25 nodes)
23
22
21
20
19
18
17
16
15
14
13
12
11
10
9
8
7
6
5
4
3
2
1
0
YFAE2
YFAE3
YFAE4
AFAE1
AFAE2
AFAE4
YVE1
YVE2
YVE3
YVE4
AVE1
AVE2
AVE3
AVE4
AVE5
AVE6
YIL1
YIL2
YIL3
YIL4
AIL1
AIL2
AIL4
AIL6

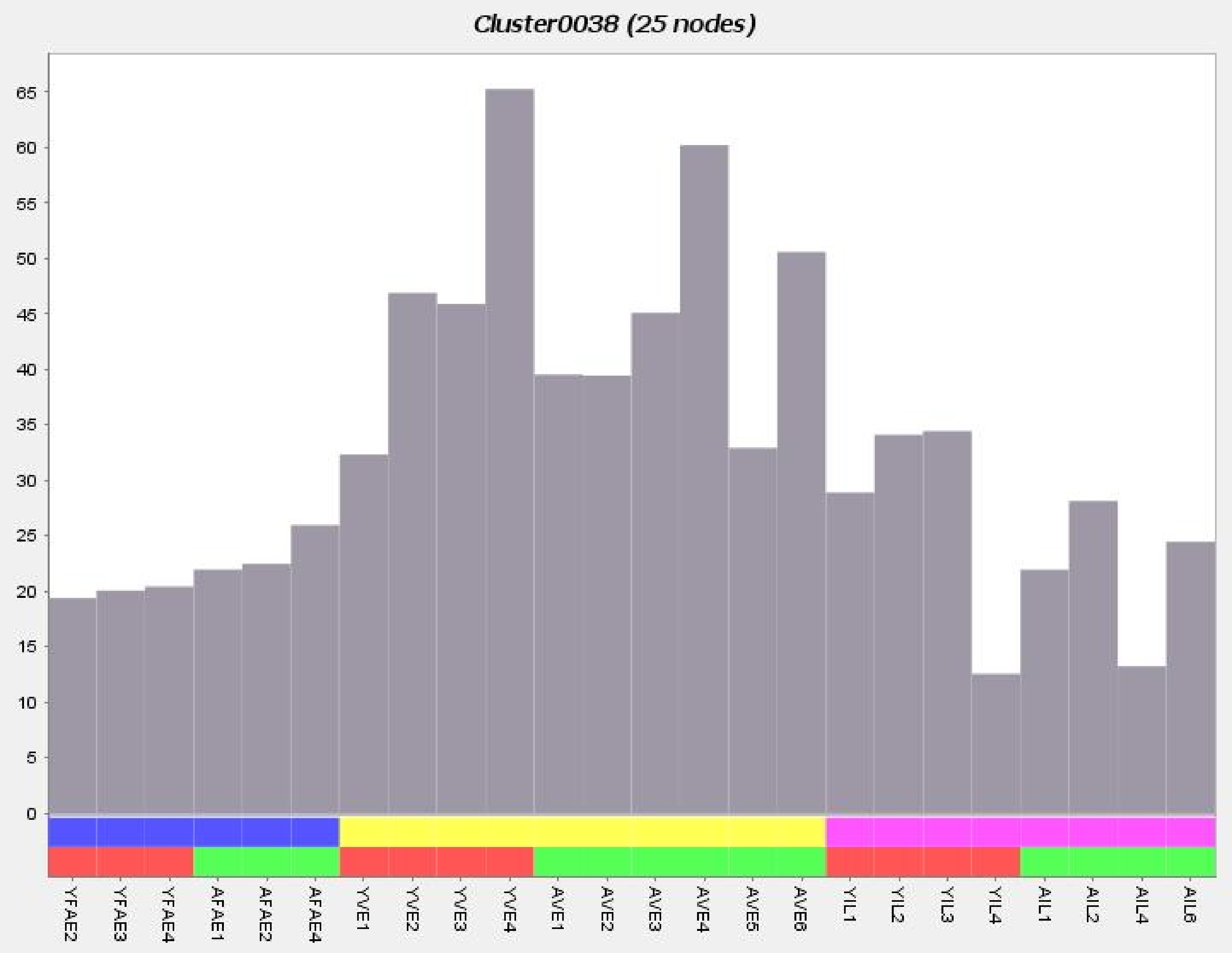

Cluster0038 (25 nodes)
65
60
55
50
45
40
35
30
25
20
15
10
5
0
YFAE2
YFAE3
YFAE4
AFAE1
AFAE2
AFAE4
YVE1
YVE2
YVE3
YVE4
AVE1
AVE2
AVE3
AVE4
AVE5
AVE6
YIL1
YIL2
YIL3
YIL4
AIL1
AIL2
AIL4
AIL6

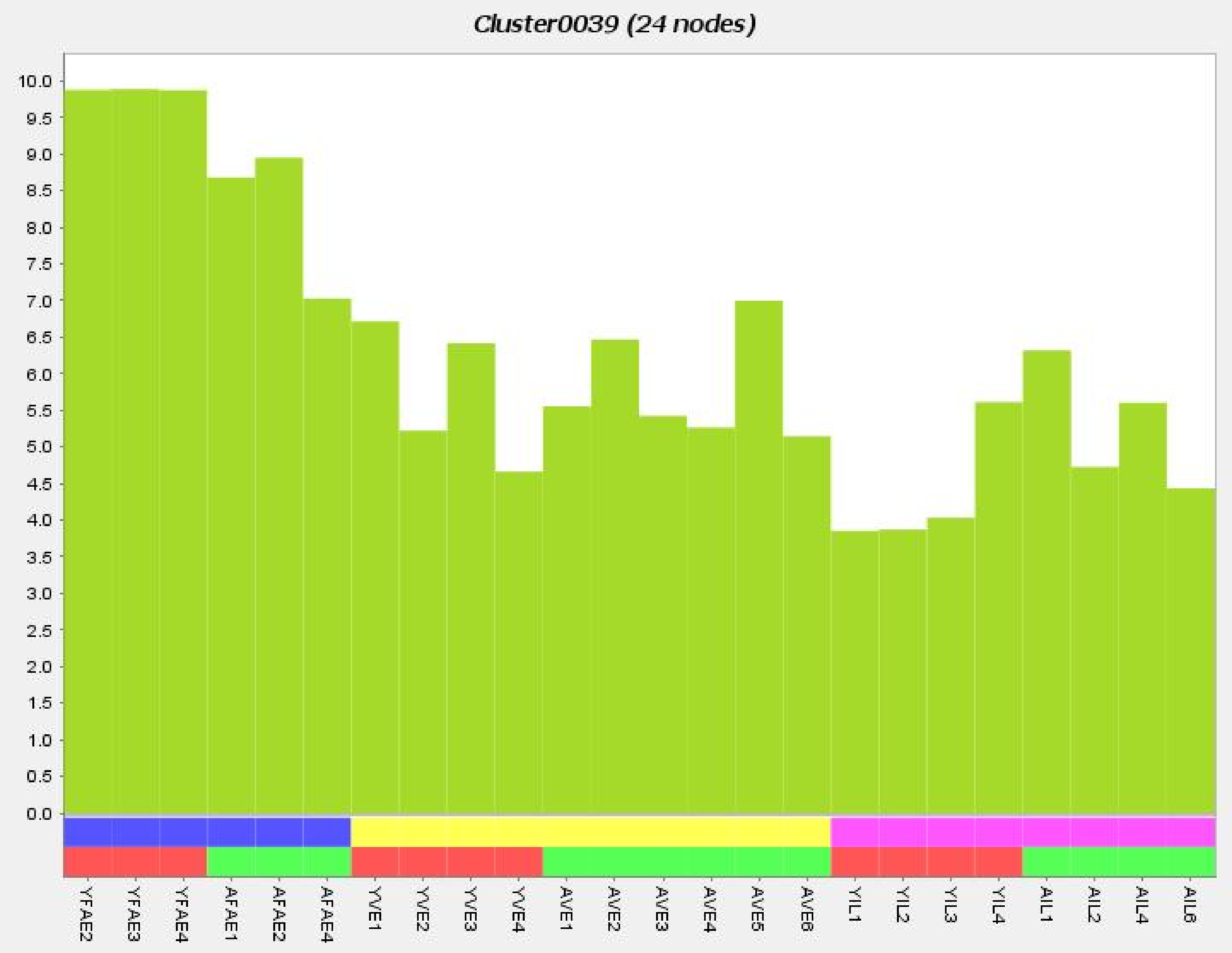

Cluster0039 (24 nodes)
10.0
9.5
9.0
8.5
8.0
7.5
7.0
6.5
6.0
5.5
5.0
4.5
4.0
3.5
3.0
2.5
2.0
1.5
1.0
0.5
0.0
YFAE2
YFAE3
YFAE4
AFAE1
AFAE2
AFAE4
YVE1
YVE2
YVE3
YVE4
AVE1
AVE2
AVE3
AVE4
AVE5
AVE6
YIL1
YIL2
YIL3
YIL4
AIL1
AIL2
AIL4
AIL6

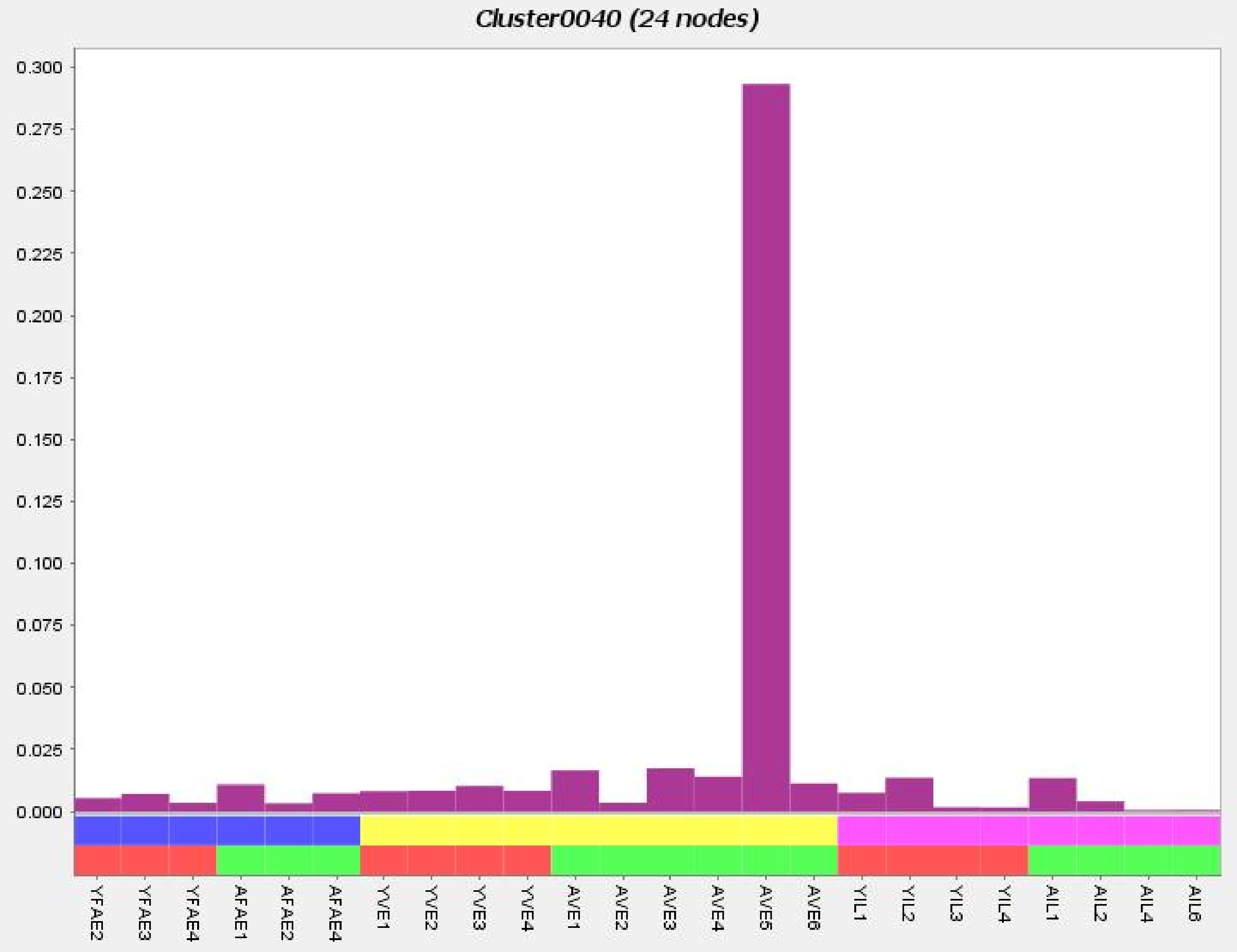
Cluster0040 (24 nodes)
0.300
0.275
0.250
0.225
0.200
0.175
0.150
0.125
0.100
0.075
0.050
0.025
0.000
YFAE2
YFAE3
YFAE4
AFAE1
AFAE2
AFAE4
YVE1
YVE2
YVE3
YVE4
AVE1
AVE2
AVE3
AVE4
AVE5
AVE6
YIL1
YIL2
YIL3
YIL4
AIL1
AIL2
AIL4
AIL6

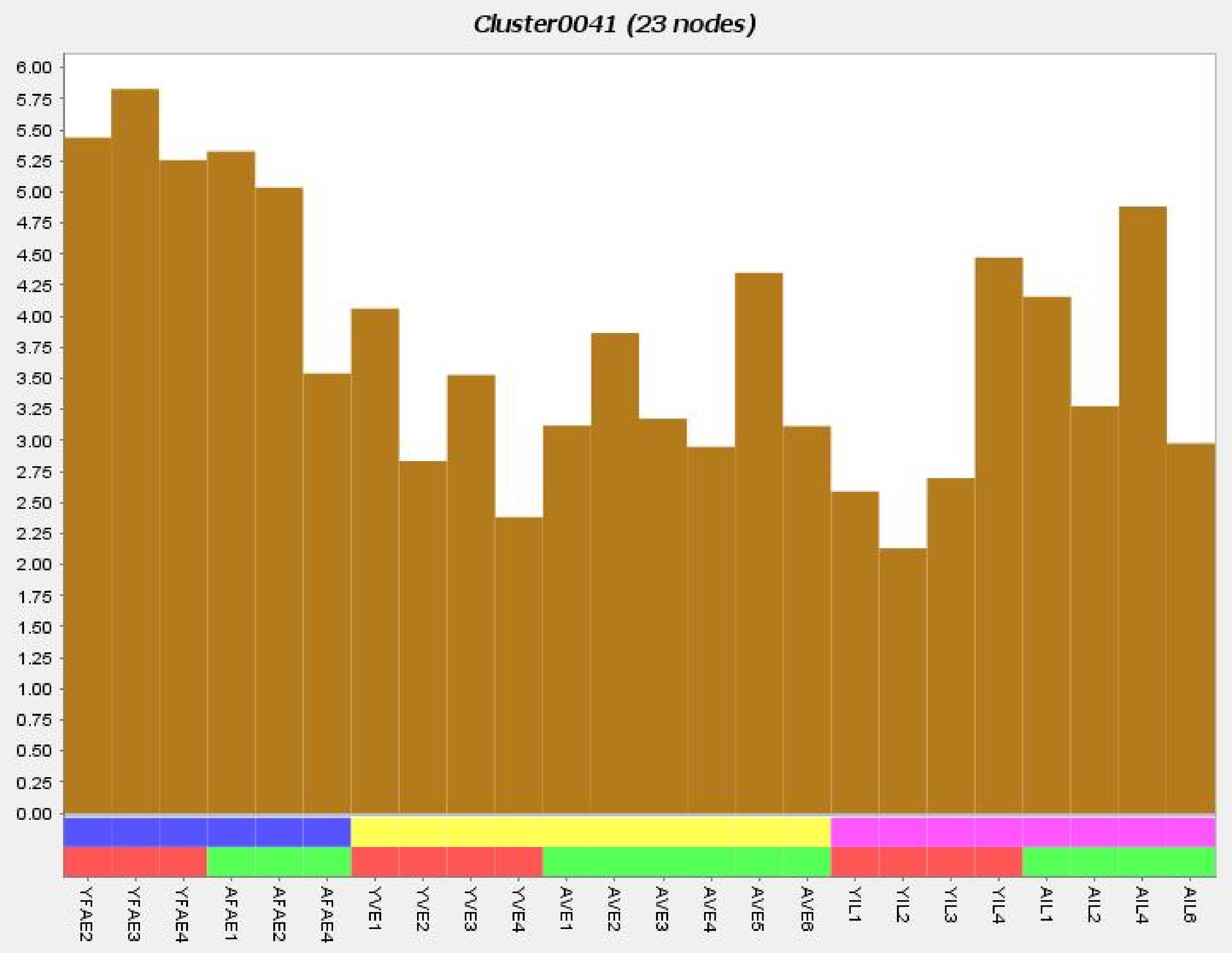
Cluster0041 (23 nodes)
6.00
5.75
5.50
5.25
5.00
4.75
4.50
4.25
4.00
3.75
3.50
3.25
3.00
2.75
2.50
2.25
2.00
1.75
1.50
1.25
1.00
0.75
0.50
0.25
0.00
YFAE2
YFAE3
YFAE4
AFAE1
AFAE2
AFAE4
YVE1
YVE2
YVE3
YVE4
AVE1
AVE2
AVE3
AVE4
AVE5
AVE6
YIL1
YIL2
YIL3
YIL4
AIL1
AIL2
AIL4
AIL6

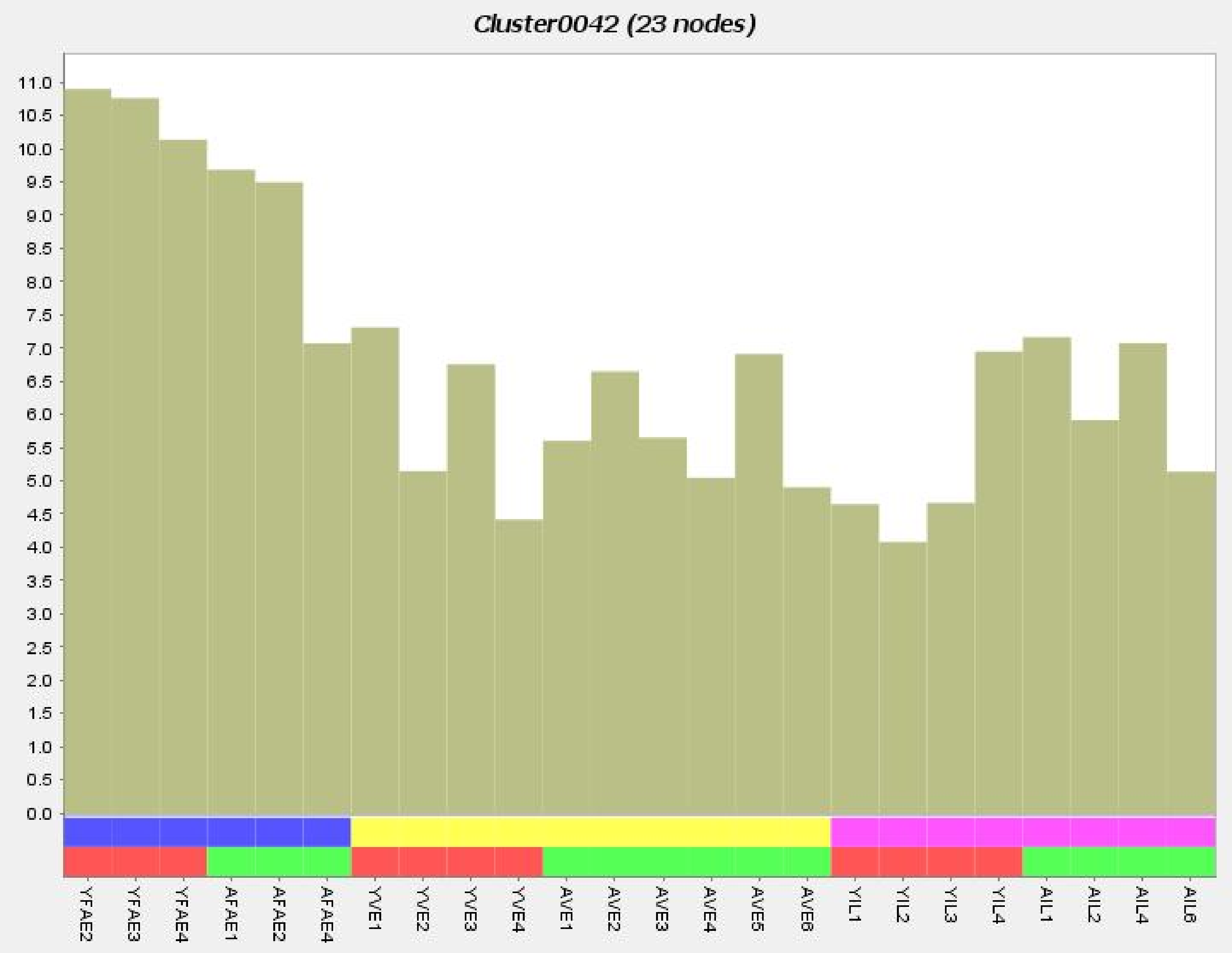
Cluster0042 (23 nodes)
11.0
10.5
10.0
9.5
9.0
8.5
8.0
7.5
7.0
6.5
6.0
5.5
5.0
4.5
4.0
3.5
3.0
2.5
2.0
1.5
1.0
0.5
0.0
YFAE2
YFAE3
YFAE4
AFAE1
AFAE2
AFAE4
YVE1
YVE2
YVE3
YVE4
AVE1
AVE2
AVE3
AVE4
AVE5
AVE6
YIL1
YIL2
YIL3
YIL4
AIL1
AIL2
AIL4
AIL6

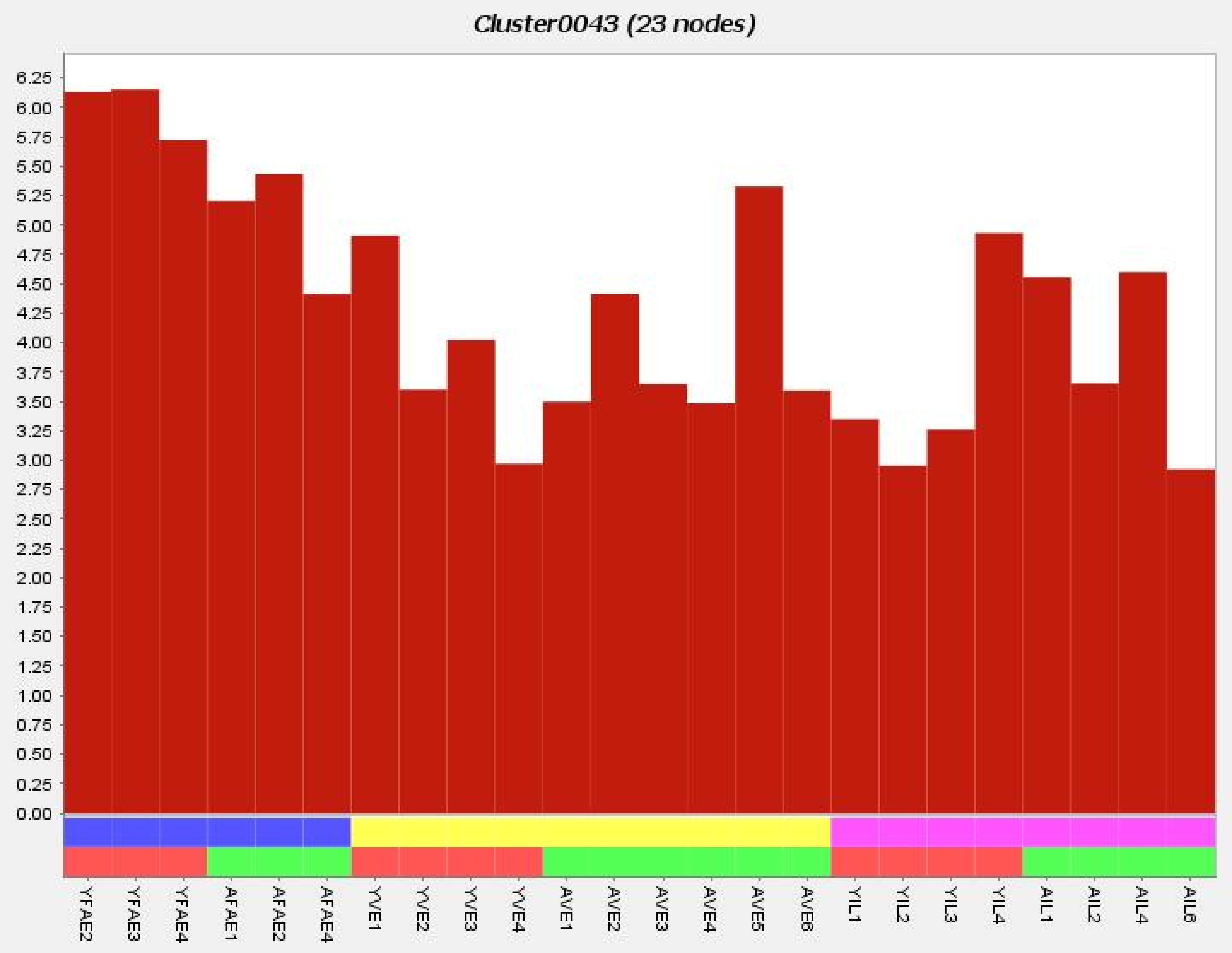
Cluster0043 (23 nodes)
6.25
6.00
5.75
5.50
5.25
5.00
4.75
4.50
4.25
4.00
3.75
3.50
3.25
3.00
2.75
2.50
2.25
2.00
1.75
1.50
1.25
1.00
0.75
0.50
0.25
0.00
YFAE2
YFAE3
YFAE4
AFAE1
AFAE2
AFAE4
YVE1
YVE2
YVE3
YVE4
AVE1
AVE2
AVE3
AVE4
AVE5
AVE6
YIL1
YIL2
YIL3
YIL4
AIL1
AIL2
AIL4
AIL6

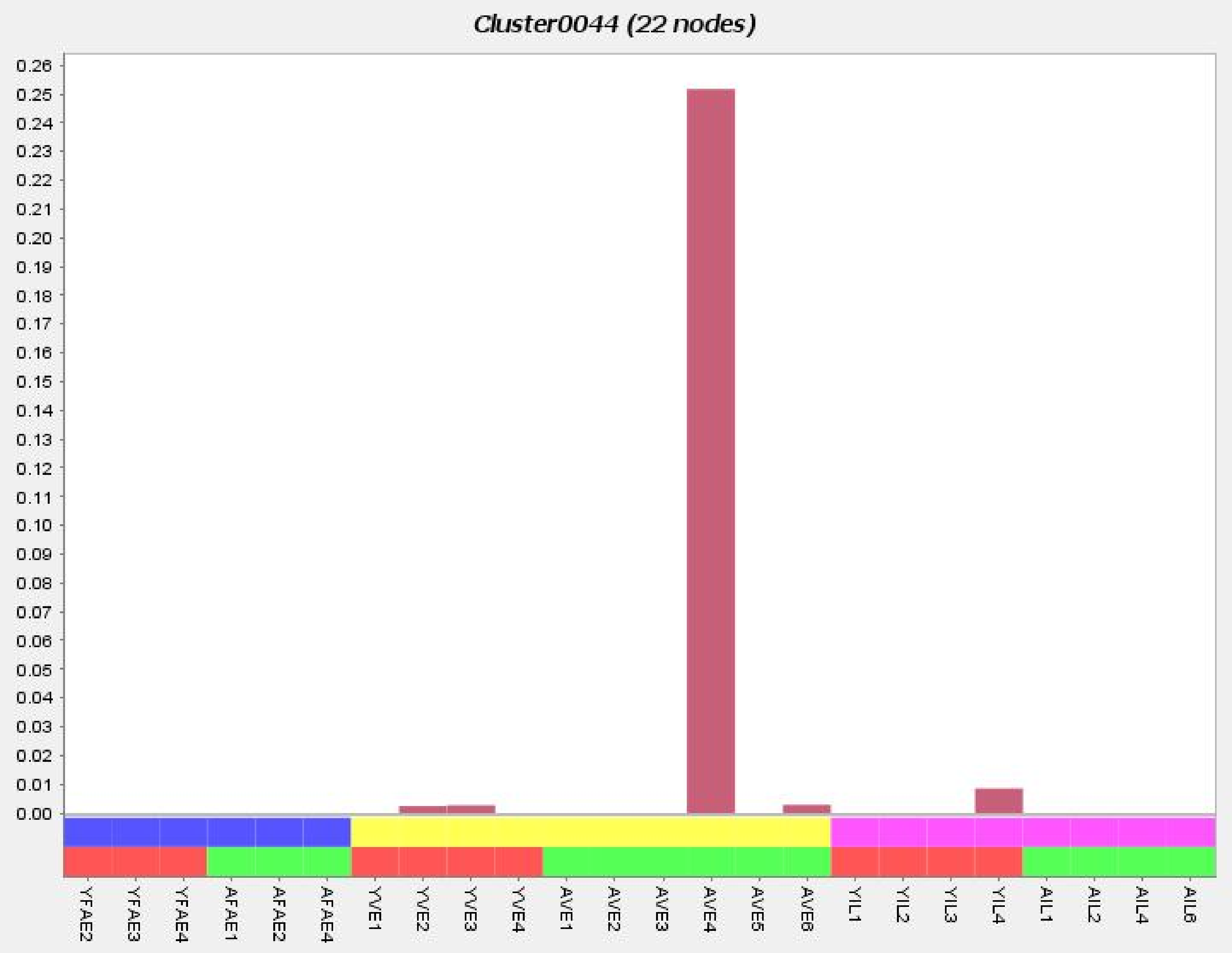

Cluster0044 (22 nodes)
0.26
0.25
0.24
0.23
0.22
0.21
0.20
0.19
0.18
0.17
0.16
0.15
0.14
0.13
0.12
0.11
0.10
0.09
0.08
0.07
0.06
0.05
0.04
0.03
0.02
0.01
0.00
YFAE2
YFAE3
YFAE4
AFAE1
AFAE2
AFAE4
YVE1
YVE2
YVE3
YVE4
AVE1
AVE2
AVE3
AVE4
AVE5
AVE6
YIL1
YIL2
YIL3
YIL4
AIL1
AIL2
AIL4
AIL6

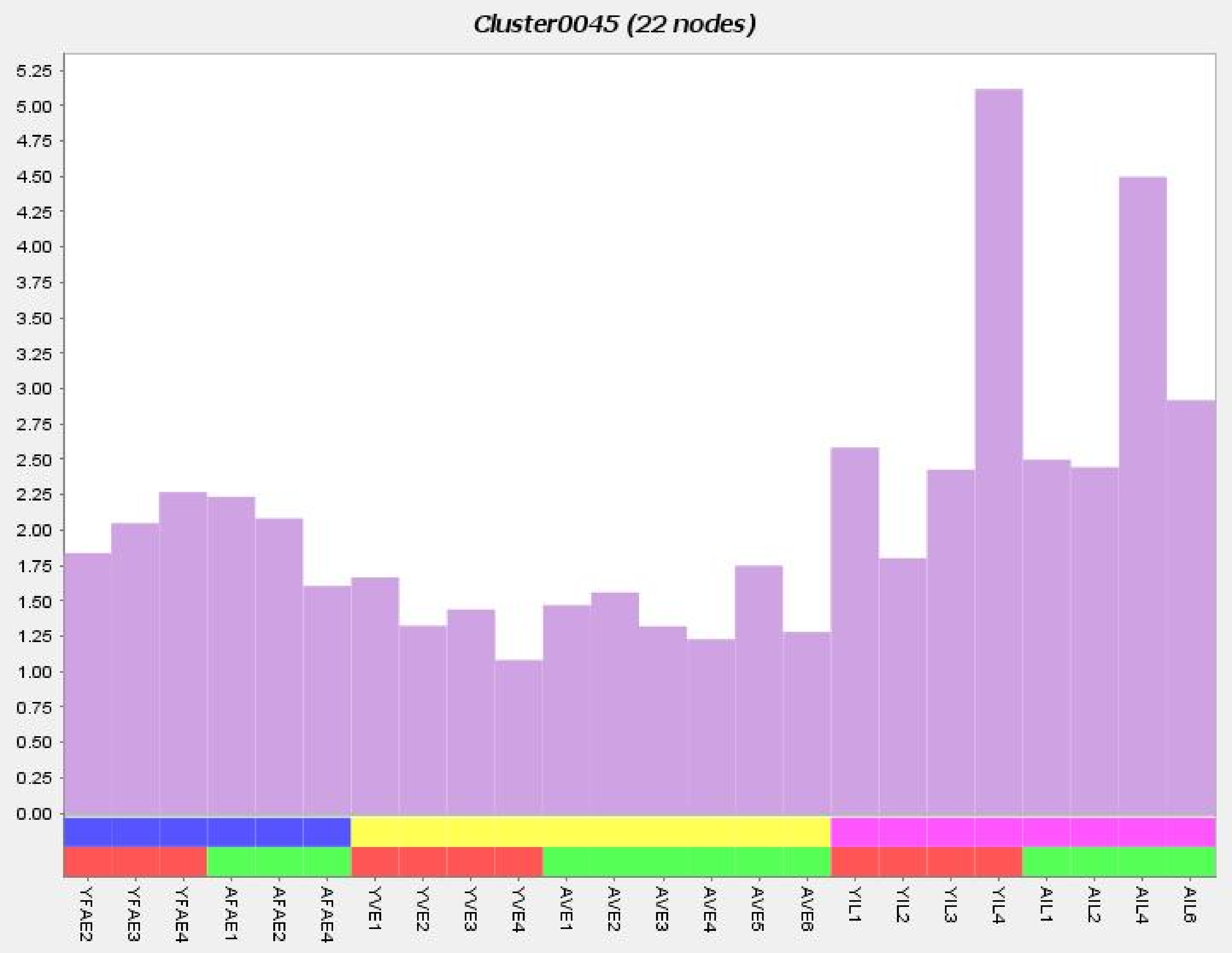

Cluster0045 (22 nodes)
5.25
5.00
4.75
4.50
4.25
4.00
3.75
3.50
3.25
3.00
2.75
2.50
2.25
2.00
1.75
1.50
1.25
1.00
0.75
0.50
0.25
0.00
YFAE2
YFAE3
YFAE4
AFAE1
AFAE2
AFAE4
YVE1
YVE2
YVE3
YVE4
AVE1
AVE2
AVE3
AVE4
AVE5
AVE6
YIL1
YIL2
YIL3
YIL4
AIL1
AIL2
AIL4
AIL6

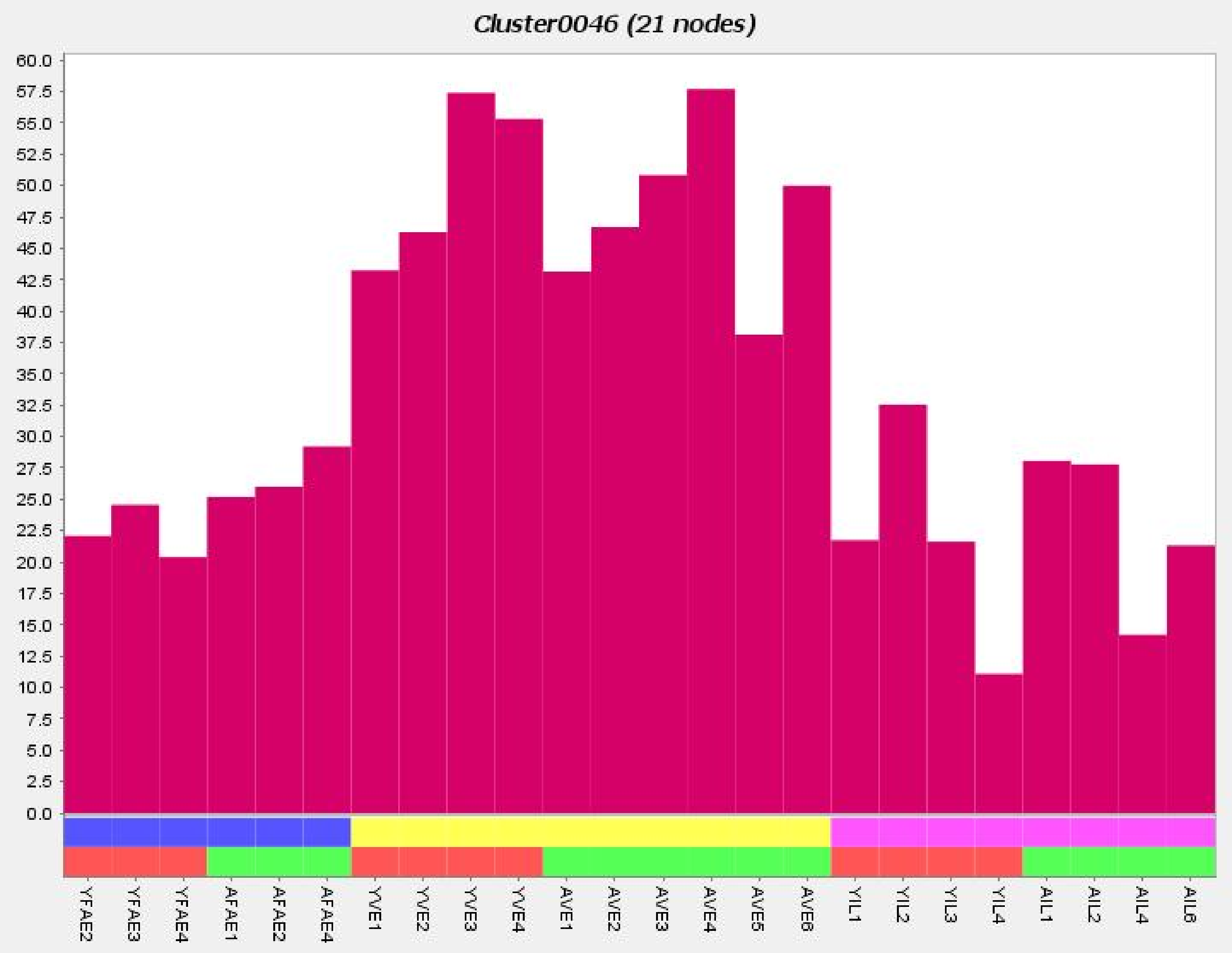
Cluster0046 (21 nodes)
60.0
57.5
55.0
52.5
50.0
47.5
45.0
42.5
40.0
37.5
35.0
32.5
30.0
27.5
25.0
22.5
20.0
17.5
15.0
12.5
10.0
7.5
5.0
2.5
0.0
YFAE2
YFAE3
YFAE4
AFAE1
AFAE2
AFAE4
YVE1
YVE2
YVE3
YVE4
AVE1
AVE2
AVE3
AVE4
AVE5
AVE6
YIL1
YIL2
YIL3
YIL4
AIL1
AIL2
AIL4
AIL6

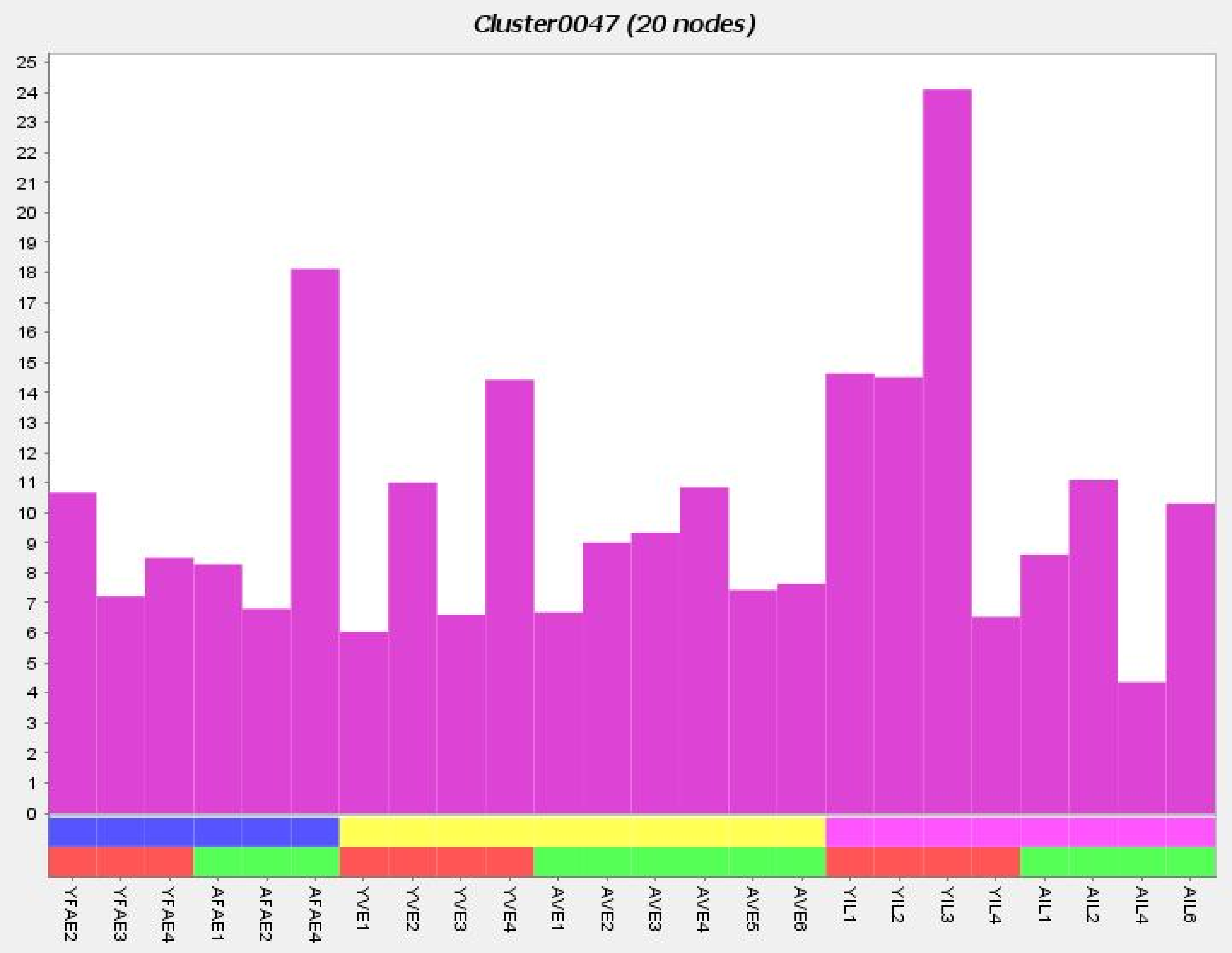

Cluster0047 (20 nodes)
25
24
23
22
21
20
19
18
17
16
15
14
13
12
11
10
9
8
7
6
5
4
3
2
1
0
YFAE2
YFAE3
YFAE4
AFAE1
AFAE2
AFAE4
YVE1
YVE2
YVE3
YVE4
AVE1
AVE2
AVE3
AVE4
AVE5
AVE6
YIL1
YIL2
YIL3
YIL4
AIL1
AIL2
AIL4
AIL6

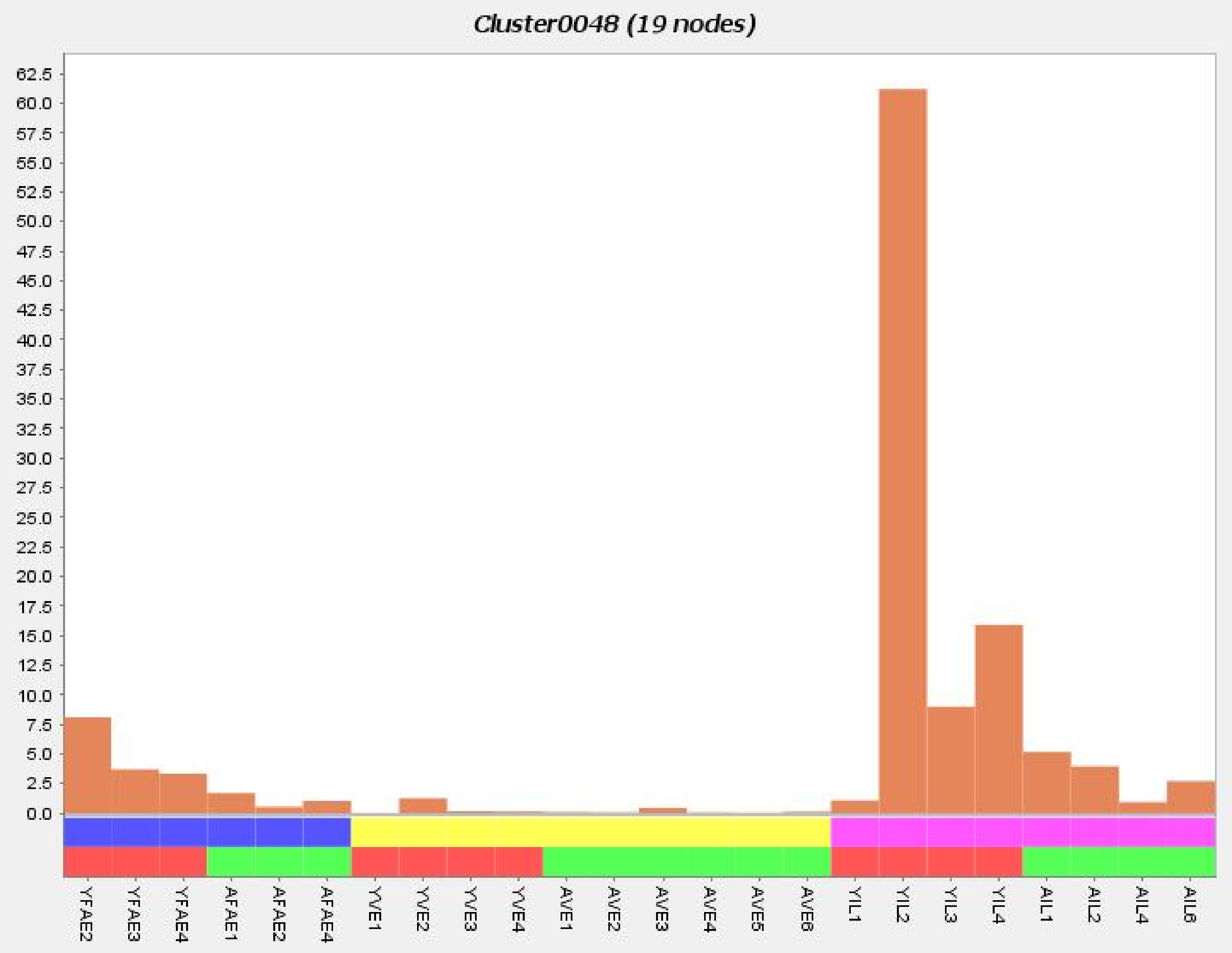
Cluster0048 (19 nodes)
62.5
60.0
57.5
55.0
52.5
50.0
47.5
45.0
42.5
40.0
37.5
35.0
32.5
30.0
27.5
25.0
22.5
20.0
17.5
15.0
12.5
10.0
7.5
5.0
2.5
0.0
YFAE2
YFAE3
YFAE4
AFAE1
AFAE2
AFAE4
YVE1
YVE2
YVE3
YVE4
AVE1
AVE2
AVE3
AVE4
AVE5
AVE6
YIL1
YIL2
YIL3
YIL4
AIL1
AIL2
AIL4
AIL6

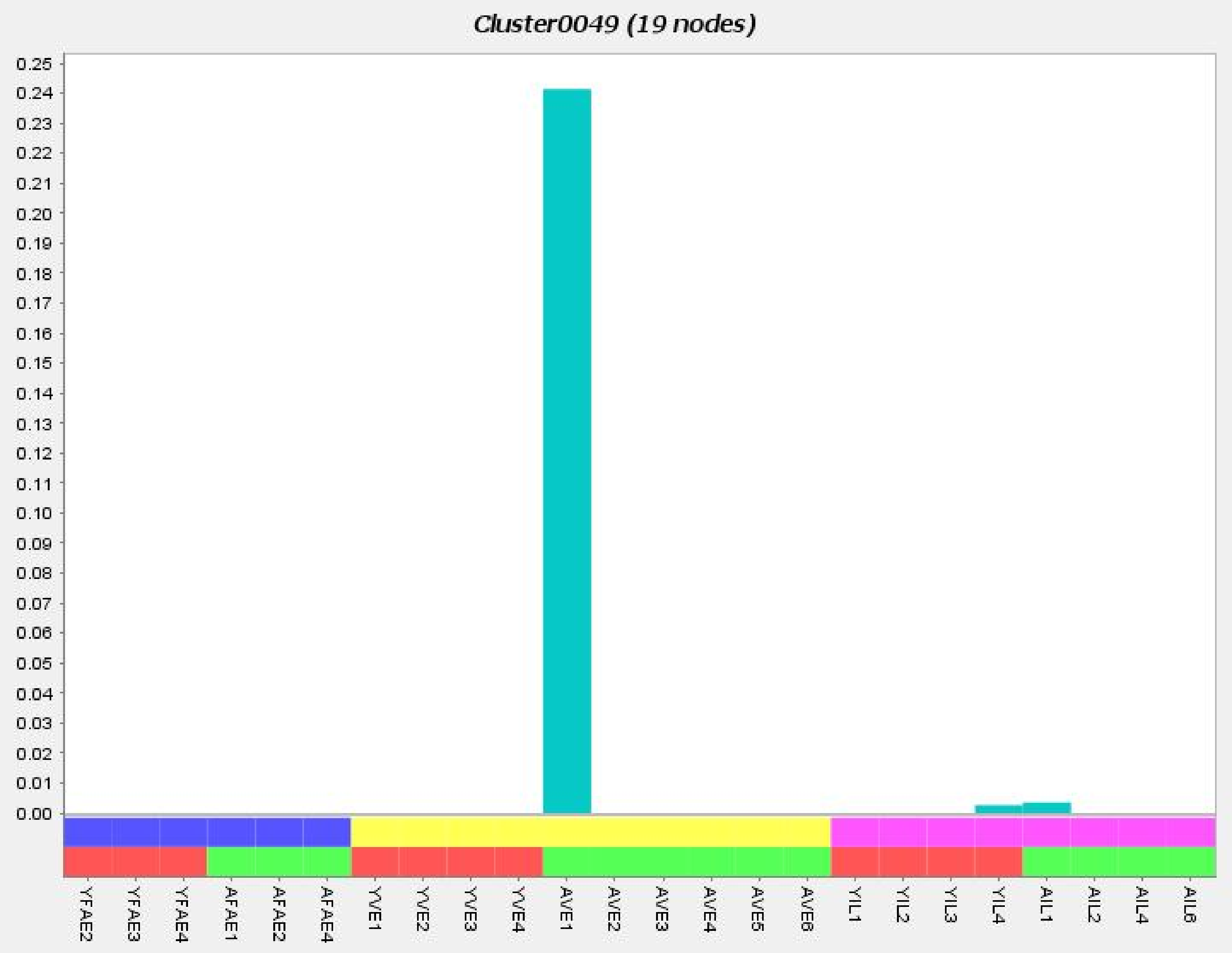
Cluster0049 (19 nodes)
0.25
0.24
0.23
0.22
0.21
0.20
0.19
0.18
0.17
0.16
0.15
0.14
0.13
0.12
0.11
0.10
0.09
0.08
0.07
0.06
0.05
0.04
0.03
0.02
0.01
0.00
YFAE2
YFAE3
YFAE4
AFAE1
AFAE2
AFAE4
YVE1
YVE2
YVE3
YVE4
AVE1
AVE2
AVE3
AVE4
AVE5
AVE6
YIL1
YIL2
YIL3
YIL4
AIL1
AIL2
AIL4
AIL6

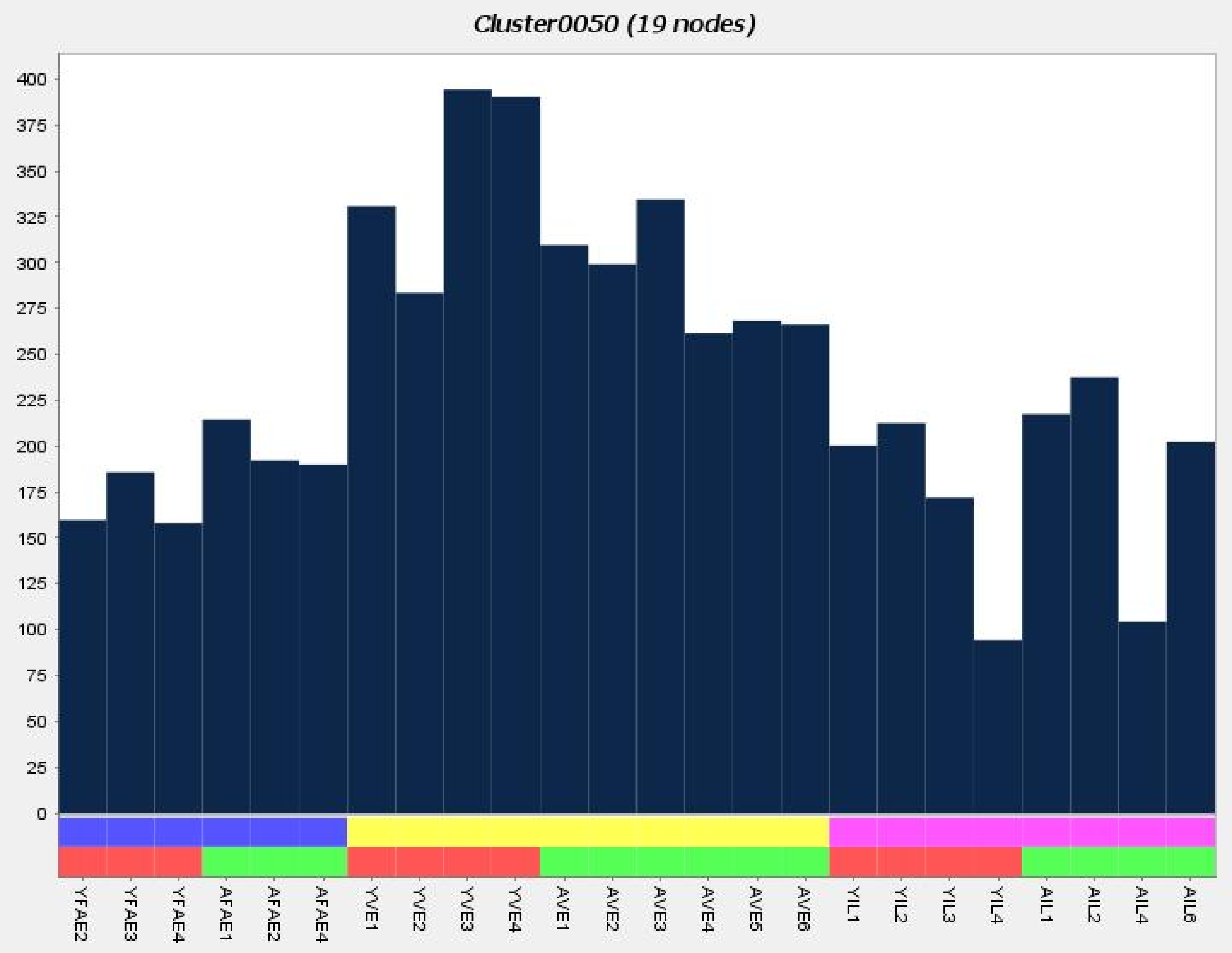
Cluster0050 (19 nodes)
400
375
350
325
300
275
250
225
200
175
150
125
100
75
50
25
0
YFAE2
YFAE3
YFAE4
AFAE1
AFAE2
AFAE4
YVE1
YVE2
YVE3
YVE4
AVE1
AVE2
AVE3
AVE4
AVE5
AVE6
YIL1
YIL2
YIL3
YIL4
AIL1
AIL2
AIL4
AIL6
